# Distribution of the genus *Platycerus* Geoffroy (Coleoptera, Lucanidae) in Italy

**DOI:** 10.3897/BDJ.12.e127088

**Published:** 2024-06-28

**Authors:** Davide Scaccini, Luca Bartolozzi, Michele Zilioli, Andrea Di Giulio, Enrico Ruzzier

**Affiliations:** 1 Department of Agronomy, Food, Natural Resources, Animals and Environment – University of Padua, Legnaro, Padua, Italy Department of Agronomy, Food, Natural Resources, Animals and Environment – University of Padua Legnaro, Padua Italy; 2 Museo Zoologico "La Specola", Florence, Italy Museo Zoologico "La Specola" Florence Italy; 3 Natural History Museum, Corso Venezia, Milan, Italy Natural History Museum, Corso Venezia Milan Italy; 4 Department of Science, Roma Tre University, viale G. Marconi 446, Rome, Italy Department of Science, Roma Tre University, viale G. Marconi 446 Rome Italy; 5 NBFC, National Biodiversity Future Center, Palermo, Italy NBFC, National Biodiversity Future Center Palermo Italy

**Keywords:** biodiversity, conservation, faunistic, forest management, stag beetles, *Platyceruscaprea*, *Platyceruscaraboides*, saproxylic

## Abstract

**Background:**

Stag beetles are saproxylic species that are associated with deadwood in the larval stage and play an important role in forest ecosystem dynamics. In Italy, *Platyceruscaprea* and *Platyceruscaraboides* are two small, elusive stag beetle species, whose known distribution is often limited or referring to old records.

**New information:**

The present contribution increases the knowledge on the Italian distribution of *P.caprea* and *P.caraboides*, adding 70% more sites when compared to previously published records. Ecological traits, especially related to the altitude-elevation distribution in Italy, are also described for these saproxylic beetles.

## Introduction

Stag beetles (Coleoptera, Lucanidae) are widely distributed throughout the world, with almost 1,600 known species in four subfamilies, namely Aesalinae MacLeay, 1819, Syndesinae MacLeay, 1819, Lampriminae MacLeay, 1819 and Lucaninae Latreille, 1804 (Maes 1992, Smith 2006, Fujita 2010, Bouchard et al. 2011). In the subfamily Lucaninae, the genus *Platycerus* Geoffroy, 1762, belonging to the monophyletic tribe Platycerini Mulsant, 1842 (Kim and Farrell 2015), includes more than 50 species associated with deciduous forests of the Northern Hemisphere (e.g. Franciscolo 1997, Klausnitzer and Sprecher-Uebersax 2008, Paulsen and Caterino 2009, Fujita 2010, Imura 2010).

Of the seven *Platycerus* species occurring in the Mediterranean Basin (Bartolozzi et al. 2016), four are recorded in Europe: *Platyceruscaprea* (De Geer, 1774), *Platyceruscaraboidescaraboides* (Linnaeus, 1758), *Platyceruspseudocaprea* Paulus, 1970 and *Platycerusspinifer* Schaufuss, 1862 (Vit and Bartolozzi 2013, Bartolozzi et al. 2016b). Despite *P.caprea* and *P.caraboides* being widely distributed in Europe, they are generally uncommon and even locally rare (e.g. Vit and Bartolozzi 2013, Bartolozzi et al. 2016a, Bartolozzi et al. 2016b, Alekseev 2018, Bartolozzi and Cianfanelli 2018, Bekchiev et al. 2018), possibly due to the short flight period of the adults and their cryptic habits (Franciscolo 1997, Holuša et al. 2006). In Italy, larvae of these two species live in deadwood, especially in hill and mountain forests across the Peninsula (e.g. Franciscolo 1997, Bartolozzi and Maggini 2007). In the European and Italian IUCN European Red List of species, *P.caprea* and *P.caraboides* are of “Least Concern” (Audisio et al. 2014, Carpaneto et al. 2015). Italian laws, such as the Regional Law 56/2000 of Tuscany, report these species amongst the taxa that require special attention for habitat conservation (Bartolozzi and Sforzi 2001, Ballerio 2003) and they are included in the saproxylic fauna of relevant interest in forests of protected areas (e.g. PSR 2012, PAER 2013, Regione Emilia Romagna 2018, PIP 2022).

Stag beetles play a vital role in forest ecosystem dynamics, especially where deadwood is abundant. Indeed, most species are saproxylic and, thus, depend on dead or decaying wood for their survival: their larvae feed on the woody material colonised by fungi and other microorganisms (e.g. Tanahashi et al. 2009, Tanahashi et al. 2017). *Platyceruscaprea* and *P.caraboides* develop in cool, temperate, broadleaf forests and specifically in humid deadwood (Klausnitzer and Sprecher-Uebersax 2008, Lachat et al. 2012, Scaccini 2016, Leidinger et al. 2021, Scaccini 2022). In these habitats, *Platycerus* species may be exploited by predators, parasites and parasitoids that represent another important element of the saproxylic community (Franciscolo 1997, Klausnitzer and Sprecher-Uebersax 2008, Scaccini 2018b, Scaccini 2020). Both *P.caprea* and *P.caraboides* develop on deadwood in a slightly advanced stage of decay, especially in logs small in diameter (< 50 cm) as occurs for *P.caraboides*, in particular (Scaccini 2016, Scaccini 2022). While *P.caraboides* seems less dependent on the available deadwood amount in the forest, *P.caprea* prefers places with a greater amount of deadwood, especially in cooler sites (Lachat et al. 2012). As reported at least for Asian species, *Platycerus* symbionts prefer cool areas for their development (Tanahashi et al. 2017, Zhu et al. 2022), possibly restricting the habitat range of their hosts that may cover cool environments like mountains in the Italian territory.

Italian occurrences of *P.caprea* and *P.caraboides* are often scattered and dated despite these species having been investigated since the 19^th^ century (e.g. Contarini 1843, Gedler 1863, Ghiliani 1887, Baudi 1889, Stolz 1915, Luigioni 1929, Porta 1932, Müller 1938, von Peez and Kahlen 1977). In these faunistic inventories, distribution data were seldom reported with the indication of precise localities, often requiring further confirmation; this might be the case of *P.caraboides* in Sardinia, reported by Luigioni (1929) and then quoted by Porta (1932) without a critical review. A detailed faunistic analysis of both species at a national scale was first provided by Dellacasa (1966) and, later, by Franciscolo (1997) and Bartolozzi and Maggini (2007), which included data collected up to 2001; a review of the distributions of the two Italian *Platycerus* for the last two decades is currently lacking.

This contribution aims to update *Platycerus* spp. distribution for Italy by revising the existing literature, summarising the present knowledge and adding new records. This overview greatly improves our understanding of *P.caprea* and *P.caraboides* occurrence in Italy and will be an important starting point for future analyses and actions aimed at the conservation of these two species.

## Materials and methods

### Geographic coverage

Data collection covered all the 20 administrative regions of Italy, with a latitudinal range from 35.490° to 47.093° N and a longitudinal range from 6.626° to 18.519° E. Since no *Platycerus* is present on Italian islands, based on the updated literature, the research was mainly focused on the mainland (18 administrative regions), which covers an area of almost 250,000 km^2^, with the southernmost point at 37.916° N.

## Data resources

**Figure 1.**
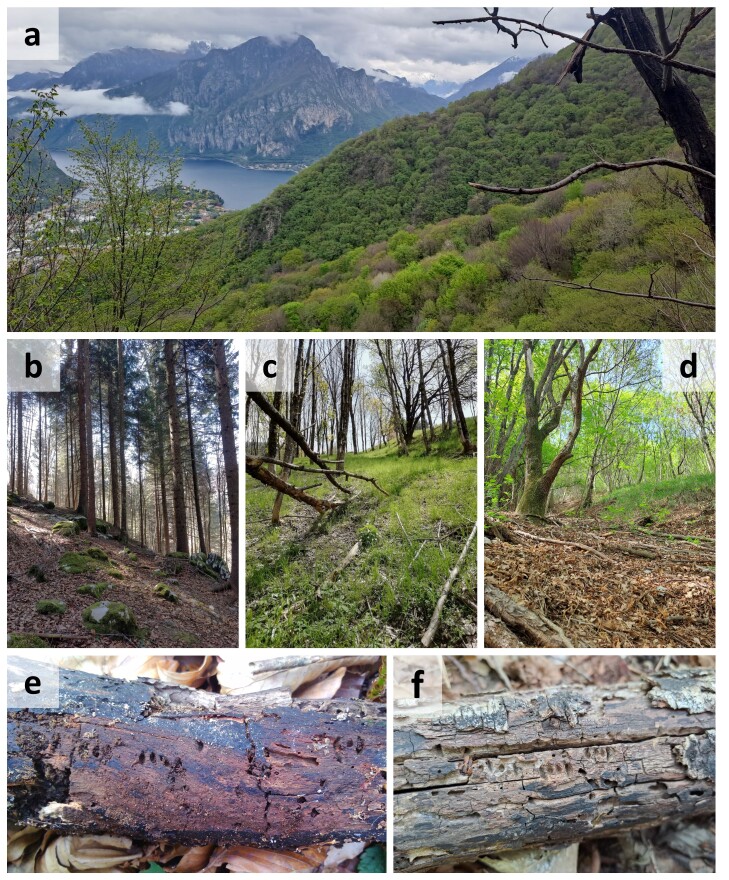
Habitats of *Platycerus* in Italy. **a** Panoramic view of biotopes of *Platyceruscaraboides* in April, Lecco Province; **b** Habitat of *Platyceruscaprea* in March, 1100 m a.s.l., Vicenza Province; **c** Habitat of *P.caraboides* in May, 1000 m a.s.l., Bergamo Province; **d** Habitat of *P.caraboides* in April, 450 m a.s.l., Treviso Province; **e, f** *Platycerus* oviposition scars on deadwood. Photo credit: D. Scaccini.

To produce the most updated set of faunistic records for both *P.caprea* and *P.caraboides* in Italy, we performed extensive data-mining on online databases and social networks, a careful revision of private and public collections (see Acknowledgements section), a review of the literature and multiple field surveys. Field surveys were conducted in various forest areas in Italy, after selecting each site by considering the available information on habitat requirements and distribution of these two species, as well as thanks to the experience on the subject of one of the authors (D. Scaccini, Fig. 1a-d). Forests with a considerable amount of deadwood were preferred during site selection. Once on site, surveys were generally conducted by searching for *Platycerus* in deadwood material like logs, stumps and snags, carefully opening them by hand, sometimes with the help of a knife or chisel. The presence of oviposition scars on the deadwood surface helped while performing this research (Scaccini 2016, Scaccini and Uliana 2017, Scaccini 2018a, Scaccini 2022, Fig. 1e,f). Data-mining was conducted on online resources such as “Forum Entomologi Italiani” and “Natura Mediterraneo”, as well as in GBIF (2023a) and GBIF (2023b). Material from all the available literature was used, especially that in Italian, English and German. Information present on specimen labels is reported *verbatim*. The data presented in this study were collected up to the 31 December 2023.

### Species identification

Stag beetles collected during field surveys were identified both at larval and adult stages with the help of a magnification lens. Adults collected in the field and those from entomological collections were identified to the specific level by inspecting their genitalia whenever it was possible, for both males and females. For males, we considered, in particular, the margins of the paramera, while for females, we inspected the morphological characters on their gonostyli (e.g. Dellacasa 1966, Franciscolo 1997, Ballerio 2003, Brustel and Van Meer 2020, Figs 2, 3). For the material retrieved online, the identification was performed on external diagnostic features of the adults as reported in the abovementioned identification keys and on their genitalia features when available. Data of insects with doubtful location and/or identification were not considered in the present work. For immature stages found during field surveys, species identification was performed on third-instar larvae using external characters following Hůrka (1975) and Franciscolo (1997), considering the observations reported by Scaccini (2015). Pupae were reared to the adult stage to validate species identification.

### Data analysis

The distribution maps of *P.caprea* and *P.caraboides* were built with QGIS (v. 3.4.2-Madeira) using a raster layer file of Italian regions retrieved from Geoportale Nazionale (2023), projected in WGS84. Points were classified as follows, according to their year of collection: (i) before 1923 (i.e. more than 100 years ago), (ii) from 1923 to 1973 (51 to 100 years ago) and (iii) from 1974 to 2023 (in the last 50 years). When information on the year was not available, we indicated a putative period (reported as "abt.") that was based on the year of publication in case of published material and on the period of activity of the collector in case of field collection data. In a few cases, when this information could not be retrieved, it was left as "n.a.". For each stag beetle species, differences in the three periods were represented by region of Italy with descriptive statistics. Elevation data for the sampled points were also taken into account, including those provided on the label and those obtained from sites with specific coordinates. When the elevation on the label was indicated as a range, in the elaboration, we considered the mean value. The occurrence of *P.caprea* and *P.caraboides* at different elevations on a latitudinal gradient was tested with linear regressions plotting seven 1°-width latitude classes (i.e. x < 41, 41 ≤ x < 42, 42 ≤ x < 43, 43 ≤ x < 44, 44 ≤ x < 45, 45 ≤ x < 46 and x ≥ 46° N) as the independent variable and the elevation as the dependent variable. Regression lines were run through an F test (α = 0.05), performed in R version 4.2.1 (R Core Team 2012) by using RStudio (RStudio Team 2016) with "stargazer" (Hlavac 2022). In this analysis, linear and quadratic models were tested for each species and comparisons between the models were made to select the one with the best fit using analysis of variance (ANOVA) and ranking the models, based on the Akaike Information Criterion (AIC).

## Taxon treatments

### Platycerus caprea

(De Geer, 1774)

2D6932CF-8948-5D52-A20B-CB39981D62CB

#### Materials

- **Type status:** Other material. **Occurrence:** recordedBy: leg. P. Cerretti; individualCount: 1; lifeStage: adult; occurrenceID: F1506AAF-20CB-5AC9-8085-3BE19CD0E70F; **Taxon:** scientificName: Platyceruscaprea (De Geer, 1774); order: Coleoptera; family: Lucanidae; **Location:** country: ITALY; countryCode: IT; stateProvince: L'Aquila; locality: Monte Ruella; verbatimElevation: 900; **Event:** year: 1996; month: 5; day: 17; **Record Level:** institutionCode: Coll. Museo Civico di Zoologia di Roma
- **Type status:** Other material. **Occurrence:** recordedBy: leg. De Vitis; individualCount: 1; sex: male; lifeStage: adult; occurrenceID: B9A08926-31A2-5FD4-9C86-94AB4F178185; **Taxon:** scientificName: Platyceruscaprea (De Geer, 1774); order: Coleoptera; family: Lucanidae; **Location:** country: ITALY; countryCode: IT; stateProvince: Teramo; municipality: Ceppo; locality: Rocca Santa Maria; verbatimElevation: 1340; decimalLatitude: 42.671389; decimalLongitude: 13.465; geodeticDatum: WGS84; **Event:** year: 2019; month: 6; day: 4; **Record Level:** institutionCode: Coll. F. Tomasi
- **Type status:** Other material. **Occurrence:** recordedBy: leg. Nappini; individualCount: 7; lifeStage: adult; occurrenceID: E4D50666-F176-5F6B-90F6-A343276EE681; **Taxon:** scientificName: Platyceruscaprea (De Geer, 1774); order: Coleoptera; family: Lucanidae; **Location:** country: ITALY; countryCode: IT; stateProvince: Potenza; municipality: Abriola; verbatimElevation: 1350; **Event:** year: 2022; month: 4; day: 25
- **Type status:** Other material. **Occurrence:** recordedBy: leg. D. Patacchiola; individualCount: 5; sex: 2 males, 3 females; lifeStage: adult; occurrenceID: FFC6AAD4-673E-557F-8894-3BD7A47337AF; **Taxon:** scientificName: Platyceruscaprea (De Geer, 1774); order: Coleoptera; family: Lucanidae; **Location:** country: ITALY; countryCode: IT; locality: Monte Sila, Botte Donato, Strada delle Vette; verbatimElevation: 1400; **Event:** year: 2022; month: 5; day: 26
- **Type status:** Other material. **Occurrence:** individualCount: 1; lifeStage: adult; occurrenceID: FCCD2C37-0825-5EC6-9AB1-2B62AD57C495; **Taxon:** scientificName: Platyceruscaprea (De Geer, 1774); order: Coleoptera; family: Lucanidae; **Location:** country: ITALY; countryCode: IT; stateProvince: Salerno; locality: Monte Cervati; verbatimElevation: 1800; **Event:** year: 2018; month: 5; day: 26; **Record Level:** institutionCode: Coll. A. Carlin
- **Type status:** Other material. **Occurrence:** individualCount: 1; sex: male; lifeStage: adult; occurrenceID: 2DB8F49F-481C-5084-AABA-D035E6E36F8F; **Taxon:** scientificName: Platyceruscaprea (De Geer, 1774); order: Coleoptera; family: Lucanidae; **Location:** country: ITALY; countryCode: IT; stateProvince: Forlì-Cesena; locality: Campigna; **Event:** year: 1948; month: 6; day: 6; **Record Level:** institutionCode: Coll. Museo Civico di Storia Naturale di Milano
- **Type status:** Other material. **Occurrence:** recordedBy: leg. Callegari; individualCount: 1; sex: male; lifeStage: adult; occurrenceID: 5F7B6774-7557-5EF6-A9EF-990236D208FB; **Taxon:** scientificName: Platyceruscaprea (De Geer, 1774); order: Coleoptera; family: Lucanidae; **Location:** country: ITALY; countryCode: IT; stateProvince: Forlì-Cesena; locality: Foresta di Campigna; **Event:** year: 2001; month: 6; day: 17; **Record Level:** institutionCode: Coll. Museo di Storia Naturale di Venezia (ex coll. F. Callegari)
- **Type status:** Other material. **Occurrence:** recordedBy: leg. F. Bin; individualCount: 1; sex: male; lifeStage: adult; occurrenceID: 07FD33BB-E46D-5887-A96B-0B61A9111415; **Taxon:** scientificName: Platyceruscaprea (De Geer, 1774); order: Coleoptera; family: Lucanidae; **Location:** country: ITALY; countryCode: IT; stateProvince: Parma; municipality: Corniglio; locality: Lago Santo; **Event:** year: 1968; month: 6; day: 23; **Record Level:** institutionCode: Coll. Museo Civico di Storia Naturale di Milano
- **Type status:** Other material. **Occurrence:** recordedBy: leg. F. Bin; individualCount: 1; sex: male; lifeStage: adult; occurrenceID: E763F51C-50DD-5139-8750-0729E79219F7; **Taxon:** scientificName: Platyceruscaprea (De Geer, 1774); order: Coleoptera; family: Lucanidae; **Location:** country: ITALY; countryCode: IT; stateProvince: Piacenza; municipality: Ferriere; locality: Groppo delle Ali; verbatimElevation: 1600; **Event:** year: 1967; month: 5; day: 20; **Record Level:** institutionCode: Coll. Museo Civico di Storia Naturale di Milano
- **Type status:** Other material. **Occurrence:** individualCount: 1; sex: female; lifeStage: adult; occurrenceID: E4072746-1453-5050-8E17-48C445B59E19; **Taxon:** scientificName: Platyceruscaprea (De Geer, 1774); order: Coleoptera; family: Lucanidae; **Location:** country: ITALY; countryCode: IT; stateProvince: Pordenone; locality: Bosco del Cansiglio; **Event:** year: 1956; month: 6; day: 8; **Record Level:** institutionCode: Coll. Museo di Storia Naturale di Venezia (ex coll. G. Agazzi)
- **Type status:** Other material. **Occurrence:** recordedBy: leg. C. Alzona; individualCount: 1; sex: male; lifeStage: adult; occurrenceID: 3088962A-3674-580B-B3D6-AF42A2E5880C; **Taxon:** scientificName: Platyceruscaprea (De Geer, 1774); order: Coleoptera; family: Lucanidae; **Location:** country: ITALY; countryCode: IT; stateProvince: Udine; municipality: Riofreddo; locality: Alpi Giulie; **Event:** year: 1951; month: 7; **Record Level:** institutionCode: Coll. Museo Civico di Storia Naturale di Milano
- **Type status:** Other material. **Occurrence:** recordedBy: leg. L. Deganutti; sex: several m. and f.; lifeStage: adult; occurrenceID: 8D2EABA2-CE37-5872-9BBB-2AC7B9D3B694; **Taxon:** scientificName: Platyceruscaprea (De Geer, 1774); order: Coleoptera; family: Lucanidae; **Location:** country: ITALY; countryCode: IT; stateProvince: Udine; municipality: Tarvisio; locality: Cernkerla; verbatimElevation: 957; decimalLatitude: 46.48592; decimalLongitude: 13.6332; geodeticDatum: WGS84; **Event:** year: 2023; month: May/June
- **Type status:** Other material. **Occurrence:** recordedBy: leg. L. Deganutti; individualCount: 4; sex: males; lifeStage: adult; occurrenceID: A77E7033-FD3A-5A0C-904F-351D74EFA29E; **Taxon:** scientificName: Platyceruscaprea (De Geer, 1774); order: Coleoptera; family: Lucanidae; **Location:** country: ITALY; countryCode: IT; stateProvince: Udine; municipality: Tarvisio; locality: Coccau; verbatimElevation: 935; decimalLatitude: 46.53189; decimalLongitude: 13.62325; geodeticDatum: WGS84; **Event:** year: 2023; month: May/June
- **Type status:** Other material. **Occurrence:** recordedBy: leg. L. Deganutti; sex: several m. and f.; lifeStage: adult; occurrenceID: 52F4FFFC-0D64-540E-85C3-A4070797014C; **Taxon:** scientificName: Platyceruscaprea (De Geer, 1774); order: Coleoptera; family: Lucanidae; **Location:** country: ITALY; countryCode: IT; stateProvince: Udine; municipality: Tarvisio; locality: Lago superiore di Fusine; verbatimElevation: 944; decimalLatitude: 46.47157; decimalLongitude: 13.67272; geodeticDatum: WGS84; **Event:** year: 2023; month: May/June
- **Type status:** Other material. **Occurrence:** recordedBy: leg. L. Deganutti; sex: several m. and f.; lifeStage: adult; occurrenceID: 11B02D26-7E3F-5001-B835-463640F3188D; **Taxon:** scientificName: Platyceruscaprea (De Geer, 1774); order: Coleoptera; family: Lucanidae; **Location:** country: ITALY; countryCode: IT; stateProvince: Udine; municipality: Tarvisio; locality: Schönek; verbatimElevation: 1075; decimalLatitude: 46.48204; decimalLongitude: 13.63282; geodeticDatum: WGS84; **Event:** year: 2023; month: May/June
- **Type status:** Other material. **Occurrence:** recordedBy: leg. L. Deganutti; sex: several m. and f.; lifeStage: adult; occurrenceID: 377E64B7-BF82-51A5-AB96-618558929270; **Taxon:** scientificName: Platyceruscaprea (De Geer, 1774); order: Coleoptera; family: Lucanidae; **Location:** country: ITALY; countryCode: IT; stateProvince: Udine; municipality: Tarvisio; locality: Tamer (Alpe del Lago); verbatimElevation: 1013; decimalLatitude: 46.45873; decimalLongitude: 13.66645; geodeticDatum: WGS84; **Event:** year: 2023; month: May/June
- **Type status:** Other material. **Occurrence:** recordedBy: leg. E. Bernabò; individualCount: 1; sex: male; lifeStage: adult; occurrenceID: 90101768-E02E-5291-BF7B-DA004D1EA0CA; **Taxon:** scientificName: Platyceruscaprea (De Geer, 1774); order: Coleoptera; family: Lucanidae; **Location:** country: ITALY; countryCode: IT; stateProvince: Udine; municipality: Valbruna; **Event:** year: 1980; month: 7; day: 13; **Record Level:** institutionCode: Coll. Museo Civico di Storia Naturale di Carmagnola
- **Type status:** Other material. **Occurrence:** recordedBy: leg. C. Menozzi; individualCount: 2; sex: 1 male, 1 female; lifeStage: adult; occurrenceID: 36445C6D-D93F-5F9D-B8EB-FACB7FAD8CC8; **Taxon:** scientificName: Platyceruscaprea (De Geer, 1774); order: Coleoptera; family: Lucanidae; **Location:** country: ITALY; countryCode: IT; stateProvince: Genova; locality: Monte Penna; **Event:** year: 1937; month: 9; day: 6; **Record Level:** institutionCode: Coll. Museo Civico di Storia Naturale di Milano
- **Type status:** Other material. **Occurrence:** recordedBy: leg. E. Bernabò; individualCount: 2; sex: females; lifeStage: adult; occurrenceID: 008D4565-8F6E-5E45-942B-755489795094; **Taxon:** scientificName: Platyceruscaprea (De Geer, 1774); order: Coleoptera; family: Lucanidae; **Location:** country: ITALY; countryCode: IT; stateProvince: Genova; municipality: Monte Montarlone; locality: Val Trebbia; **Event:** year: 1977; month: 4; day: 24; **Record Level:** institutionCode: Coll. Museo Civico di Storia Naturale di Carmagnola
- **Type status:** Other material. **Occurrence:** recordedBy: leg. Callegari; individualCount: 1; sex: female; lifeStage: adult; occurrenceID: 53037B16-9649-5E78-9291-35C68B38EACB; **Taxon:** scientificName: Platyceruscaprea (De Geer, 1774); order: Coleoptera; family: Lucanidae; **Location:** country: ITALY; countryCode: IT; stateProvince: Imola; locality: Val Tanarello; verbatimElevation: 1000; **Event:** year: 1994; month: 6; day: 7-15 (mean 11); **Record Level:** institutionCode: Coll. Museo di Storia Naturale di Venezia (ex coll. F. Callegari)
- **Type status:** Other material. **Occurrence:** individualCount: 2; sex: males; lifeStage: adult; occurrenceID: 56081283-9996-5E28-BB8E-FADE74C9C55F; **Taxon:** scientificName: Platyceruscaprea (De Geer, 1774); order: Coleoptera; family: Lucanidae; **Location:** country: ITALY; countryCode: IT; locality: Manderiolo (?); **Event:** year: 1952; month: 5; **Record Level:** institutionCode: Coll. Museo Civico di Storia Naturale di Milano
- **Type status:** Other material. **Occurrence:** recordedBy: leg. L. Galbiati; individualCount: 1; sex: male; lifeStage: adult; occurrenceID: 4CCEF73A-79BB-5001-A65F-BDFF2E01792B; **Taxon:** scientificName: Platyceruscaprea (De Geer, 1774); order: Coleoptera; family: Lucanidae; **Location:** country: ITALY; countryCode: IT; stateProvince: Bergamo; locality: Monte Venturosa; **Event:** year: 1988; month: 6; day: 6; **Record Level:** institutionCode: Coll. Museo Civico di Storia Naturale di Milano
- **Type status:** Other material. **Occurrence:** recordedBy: leg. D. Pedersoli; individualCount: 1; sex: female; lifeStage: adult; occurrenceID: E9D6A031-0662-53D6-9DE3-CA720AF23D0B; **Taxon:** scientificName: Platyceruscaprea (De Geer, 1774); order: Coleoptera; family: Lucanidae; **Location:** country: ITALY; countryCode: IT; stateProvince: Bergamo; municipality: Brumano; locality: rifugio Azzoni; verbatimElevation: 1850; decimalLatitude: 45.858196; decimalLongitude: 9.470646; geodeticDatum: WGS84; **Event:** year: 2018; month: 6; day: 18; **Record Level:** institutionCode: Coll. D. Pedersoli
- **Type status:** Other material. **Occurrence:** recordedBy: leg. W. Zucchelli; individualCount: 1; lifeStage: adult; occurrenceID: D4C0DAD8-0C75-520C-9064-D51DD351E80E; **Taxon:** scientificName: Platyceruscaprea (De Geer, 1774); order: Coleoptera; family: Lucanidae; **Location:** country: ITALY; countryCode: IT; stateProvince: Bergamo; municipality: Camerata Cornello; locality: trail to passo Grialeggio; verbatimElevation: 1450; **Event:** year: 2009; month: 4; day: 30; **Record Level:** institutionCode: Coll. Museo Civico di Scienze Naturali Enrico Caffi di Bergamo
- **Type status:** Other material. **Occurrence:** recordedBy: leg. D. Scaccini; individualCount: 1; lifeStage: larva; occurrenceID: E8989560-48C8-554A-8707-0D7109249EDF; **Taxon:** scientificName: Platyceruscaprea (De Geer, 1774); order: Coleoptera; family: Lucanidae; **Location:** country: ITALY; countryCode: IT; stateProvince: Bergamo; municipality: Carona; verbatimElevation: 1220; decimalLatitude: 46.020467; decimalLongitude: 9.791219; geodeticDatum: WGS84; **Event:** year: 2015; month: 12; day: 6
- **Type status:** Other material. **Occurrence:** recordedBy: leg. D. Scaccini; individualCount: 1; lifeStage: larva; occurrenceID: B1CB5799-D703-516A-B10E-5D474B2E68F0; **Taxon:** scientificName: Platyceruscaprea (De Geer, 1774); order: Coleoptera; family: Lucanidae; **Location:** country: ITALY; countryCode: IT; stateProvince: Bergamo; municipality: Carona; verbatimElevation: 1202; decimalLatitude: 46.021864; decimalLongitude: 9.793178; geodeticDatum: WGS84; **Event:** year: 2015; month: 12; day: 6
- **Type status:** Other material. **Occurrence:** recordedBy: leg. F. Tomasi; individualCount: 1; lifeStage: larva; occurrenceID: 7D6C8D0A-D635-53FD-AE82-953ED5543B0D; **Taxon:** scientificName: Platyceruscaprea (De Geer, 1774); order: Coleoptera; family: Lucanidae; **Location:** country: ITALY; countryCode: IT; stateProvince: Bergamo; municipality: Cassiglio; verbatimElevation: 618; decimalLatitude: 45.965013; decimalLongitude: 9.609443; geodeticDatum: WGS84; **Event:** year: 2018; month: 8; day: 17; **Record Level:** institutionCode: Coll. F. Tomasi
- **Type status:** Other material. **Occurrence:** recordedBy: leg. D. Pedersoli; individualCount: 1; lifeStage: adult; occurrenceID: B5E79655-BC73-5101-A909-225D0CC7BF16; **Taxon:** scientificName: Platyceruscaprea (De Geer, 1774); order: Coleoptera; family: Lucanidae; **Location:** country: ITALY; countryCode: IT; stateProvince: Bergamo; municipality: Castione della Presolana; locality: close to Baita Cassinelli; verbatimElevation: 1600; decimalLatitude: 45.942887; decimalLongitude: 10.077021; geodeticDatum: WGS84; **Event:** year: 2006; month: 6; day: 25; **Record Level:** institutionCode: Coll. D. Pedersoli
- **Type status:** Other material. **Occurrence:** recordedBy: leg. P. Pantini; individualCount: 1; lifeStage: adult; occurrenceID: 2A7D56C3-30F6-59AE-A12C-6BDD9BFB2DA3; **Taxon:** scientificName: Platyceruscaprea (De Geer, 1774); order: Coleoptera; family: Lucanidae; **Location:** country: ITALY; countryCode: IT; stateProvince: Bergamo; municipality: Mezzoldo; verbatimElevation: 1800; **Event:** year: 2005; month: 6; day: 5; **Record Level:** institutionCode: Coll. Museo Civico di Scienze Naturali Enrico Caffi di Bergamo
- **Type status:** Other material. **Occurrence:** recordedBy: leg. D. Pedersoli; individualCount: 3; sex: 2 males, 1 female; lifeStage: adult; occurrenceID: 6A47AA0A-BAC0-5074-B6AE-00CB48670873; **Taxon:** scientificName: Platyceruscaprea (De Geer, 1774); order: Coleoptera; family: Lucanidae; **Location:** country: ITALY; countryCode: IT; stateProvince: Bergamo; municipality: Oltre il Colle; locality: close to "rifugio Cà d'Arera"; verbatimElevation: 1560; decimalLatitude: 45.916607; decimalLongitude: 9.794777; geodeticDatum: WGS84; **Event:** year: 2015; month: 6; day: 20; **Record Level:** institutionCode: Coll. D. Pedersoli
- **Type status:** Other material. **Occurrence:** recordedBy: leg. D. Pedersoli; individualCount: 3; sex: 2 males, 1 female; lifeStage: adult; occurrenceID: 89E098C2-B647-599C-90C5-4B733BB9AC09; **Taxon:** scientificName: Platyceruscaprea (De Geer, 1774); order: Coleoptera; family: Lucanidae; **Location:** country: ITALY; countryCode: IT; stateProvince: Bergamo; municipality: Plassa; verbatimElevation: 1583; decimalLatitude: 45.917163; decimalLongitude: 9.795349; geodeticDatum: WGS84; **Event:** year: 2015; month: 6; day: 20; **Record Level:** institutionCode: Coll. D. Pedersoli
- **Type status:** Other material. **Occurrence:** recordedBy: leg. D. Scaccini; individualCount: 1; sex: female; lifeStage: adult; occurrenceID: 85555403-3738-52E2-B327-2BB622575DA4; **Taxon:** scientificName: Platyceruscaprea (De Geer, 1774); order: Coleoptera; family: Lucanidae; **Location:** country: ITALY; countryCode: IT; stateProvince: Bergamo; municipality: Roncobello; verbatimElevation: 1116; decimalLatitude: 45.952194; decimalLongitude: 9.748489; geodeticDatum: WGS84; **Event:** year: 2018; month: 12; day: 30
- **Type status:** Other material. **Occurrence:** recordedBy: leg. D. Pedersoli; individualCount: 1; lifeStage: adult; occurrenceID: E34149C0-ECDF-584E-BE7B-B8416DFCF7CF; **Taxon:** scientificName: Platyceruscaprea (De Geer, 1774); order: Coleoptera; family: Lucanidae; **Location:** country: ITALY; countryCode: IT; stateProvince: Bergamo; municipality: Schilpario; locality: Val dei Gatti; verbatimElevation: 1200; decimalLatitude: 46.011724; decimalLongitude: 10.168437; geodeticDatum: WGS84; **Event:** year: 2020; month: 6; day: 18; **Record Level:** institutionCode: Coll. D. Pedersoli
- **Type status:** Other material. **Occurrence:** individualCount: 22; sex: males; lifeStage: adult; occurrenceID: 51B6E41A-A0AB-5541-8711-26BD4BB80AAB; **Taxon:** scientificName: Platyceruscaprea (De Geer, 1774); order: Coleoptera; family: Lucanidae; **Location:** country: ITALY; countryCode: IT; stateProvince: Bergamo; municipality: Serina; locality: Col di Zambla; verbatimElevation: 1200; **Event:** year: 1969; month: 10; day: 19; **Record Level:** institutionCode: Coll. Museo Civico di Storia Naturale di Milano
- **Type status:** Other material. **Occurrence:** recordedBy: leg. D. Pedersoli; individualCount: 1; sex: female; lifeStage: adult; occurrenceID: 3689D754-FE13-5E62-B7D6-DE176F92E5AD; **Taxon:** scientificName: Platyceruscaprea (De Geer, 1774); order: Coleoptera; family: Lucanidae; **Location:** country: ITALY; countryCode: IT; stateProvince: Bergamo; municipality: Sovere; locality: close to Monte Palandone; verbatimElevation: 1300; decimalLatitude: 45.821676; decimalLongitude: 9.982213; geodeticDatum: WGS84; **Event:** year: 2017; month: 6; day: 2; **Record Level:** institutionCode: Coll. D. Pedersoli
- **Type status:** Other material. **Occurrence:** recordedBy: leg. L. Galbiati; individualCount: 1; sex: male; lifeStage: adult; occurrenceID: B13866F7-7DEF-5C45-AF09-7FBFD3081953; **Taxon:** scientificName: Platyceruscaprea (De Geer, 1774); order: Coleoptera; family: Lucanidae; **Location:** country: ITALY; countryCode: IT; stateProvince: Bergamo; municipality: Zambla Alta; **Event:** year: 1981; month: 10; day: 11; **Record Level:** institutionCode: Coll. Museo Civico di Storia Naturale di Milano
- **Type status:** Other material. **Occurrence:** recordedBy: leg. Petruzziello; individualCount: 2; lifeStage: adult; occurrenceID: 9E1745EA-AA56-53BD-B0AD-994861F7266E; **Taxon:** scientificName: Platyceruscaprea (De Geer, 1774); order: Coleoptera; family: Lucanidae; **Location:** country: ITALY; countryCode: IT; stateProvince: Brescia; locality: Passo Baremone; verbatimElevation: 1500; **Event:** year: 1998; month: 6; day: 2; **Record Level:** institutionCode: Coll. P. Leo
- **Type status:** Other material. **Occurrence:** recordedBy: leg. D. Scaccini & D. Pedersoli; individualCount: 3; sex: 2 males, 1 female; lifeStage: adult; occurrenceID: C989BF60-CD9A-5A56-84C2-CC748B117806; **Taxon:** scientificName: Platyceruscaprea (De Geer, 1774); order: Coleoptera; family: Lucanidae; **Location:** country: ITALY; countryCode: IT; stateProvince: Brescia; municipality: Artogne; locality: Prato Secondino; verbatimElevation: 1420; decimalLatitude: 45.836235; decimalLongitude: 10.217265; geodeticDatum: WGS84; **Event:** year: 2015; month: 5; day: 31; **Record Level:** institutionCode: Coll. D. Scaccini
- **Type status:** Other material. **Occurrence:** recordedBy: leg. D. Pedersoli; individualCount: 2; lifeStage: adult; occurrenceID: D7E41DED-AE24-5FAD-89A4-311D93717858; **Taxon:** scientificName: Platyceruscaprea (De Geer, 1774); order: Coleoptera; family: Lucanidae; **Location:** country: ITALY; countryCode: IT; stateProvince: Brescia; municipality: Corteno Golgi; locality: close to Malga Casazza; verbatimElevation: 1500; decimalLatitude: 46.116537; decimalLongitude: 10.229236; geodeticDatum: WGS84; **Event:** year: 2021; month: 6; day: 5; **Record Level:** institutionCode: Coll. D. Pedersoli
- **Type status:** Other material. **Occurrence:** recordedBy: leg. D. Pedersoli; individualCount: 1; lifeStage: adult; occurrenceID: 0DF70D91-8844-595E-9D8A-7F092166B4EE; **Taxon:** scientificName: Platyceruscaprea (De Geer, 1774); order: Coleoptera; family: Lucanidae; **Location:** country: ITALY; countryCode: IT; stateProvince: Brescia; municipality: Pisogne; locality: torrente Palot; verbatimElevation: 1140; decimalLatitude: 45.806375; decimalLongitude: 10.147784; geodeticDatum: WGS84; **Event:** year: 2012; month: 5; day: 23; **Record Level:** institutionCode: Coll. D. Pedersoli
- **Type status:** Other material. **Occurrence:** recordedBy: leg. D. Pedersoli; individualCount: 1; lifeStage: adult; occurrenceID: A76B0A27-B8F2-58A7-B43D-3C94765CF0A2; **Taxon:** scientificName: Platyceruscaprea (De Geer, 1774); order: Coleoptera; family: Lucanidae; **Location:** country: ITALY; countryCode: IT; stateProvince: Brescia; municipality: Saviore dell'Adamello; locality: Scale dell'Adamè; verbatimElevation: 1700; decimalLatitude: 46.082971; decimalLongitude: 10.490184; geodeticDatum: WGS84; **Event:** year: 2022; month: 5; day: 22; **Record Level:** institutionCode: Coll. D. Pedersoli
- **Type status:** Other material. **Occurrence:** recordedBy: leg. E. Scarso; individualCount: 2; lifeStage: larva; occurrenceID: 724F7E4C-9A4C-54EB-A422-2FC452A2073E; **Taxon:** scientificName: Platyceruscaprea (De Geer, 1774); order: Coleoptera; family: Lucanidae; **Location:** country: ITALY; countryCode: IT; stateProvince: Brescia; municipality: Vedeseta; verbatimElevation: 1025; decimalLatitude: 45.898611; decimalLongitude: 9.548889; geodeticDatum: WGS84; **Event:** year: 2019; month: 3; day: 22; **Record Level:** institutionCode: Coll. F. Tomasi
- **Type status:** Other material. **Occurrence:** recordedBy: leg. D. Pedersoli; individualCount: 1; lifeStage: adult; occurrenceID: DBCA2131-F53C-54FA-AB17-F35E60F05B48; **Taxon:** scientificName: Platyceruscaprea (De Geer, 1774); order: Coleoptera; family: Lucanidae; **Location:** country: ITALY; countryCode: IT; stateProvince: Brescia; municipality: Vione; locality: Predassino; verbatimElevation: 1300; decimalLatitude: 46.234978; decimalLongitude: 10.441556; geodeticDatum: WGS84; **Event:** year: 2022; month: 5; day: 28; **Record Level:** institutionCode: Coll. D. Pedersoli
- **Type status:** Other material. **Occurrence:** recordedBy: leg. Gentili; individualCount: 3; sex: 2 males, 1 female; lifeStage: adult; occurrenceID: 91DA3A7C-23A5-5398-A571-3A122A3A3940; **Taxon:** scientificName: Platyceruscaprea (De Geer, 1774); order: Coleoptera; family: Lucanidae; **Location:** country: ITALY; countryCode: IT; stateProvince: Como; locality: Parco Monte S. Primo; **Event:** year: 1957; month: 9; **Record Level:** institutionCode: Coll. Museo Civico di Storia Naturale di Milano
- **Type status:** Other material. **Occurrence:** recordedBy: leg. Gentili; individualCount: 3; sex: 2 males, 1 female; lifeStage: adult; occurrenceID: A36A80EF-54B8-5F57-99F7-24ACD01D7DD6; **Taxon:** scientificName: Platyceruscaprea (De Geer, 1774); order: Coleoptera; family: Lucanidae; **Location:** country: ITALY; countryCode: IT; stateProvince: Como; municipality: Colmine; **Event:** year: 1965; month: 3; **Record Level:** institutionCode: Coll. Museo Civico di Storia Naturale di Milano
- **Type status:** Other material. **Occurrence:** recordedBy: leg. G. Pozzi; individualCount: 1; sex: female; lifeStage: adult; occurrenceID: 0EF7EA7B-97DD-5DAB-9826-5727BC26C779; **Taxon:** scientificName: Platyceruscaprea (De Geer, 1774); order: Coleoptera; family: Lucanidae; **Location:** country: ITALY; countryCode: IT; stateProvince: Como; municipality: Orimento; verbatimElevation: 1350; **Event:** year: 1960; month: 5; day: 25; **Record Level:** institutionCode: Coll. Museo Civico di Storia Naturale di Milano
- **Type status:** Other material. **Occurrence:** recordedBy: leg. C. Brivio; individualCount: 1; sex: male; lifeStage: adult; occurrenceID: 560DFA1E-F864-5E20-8EAD-92EA632A29C7; **Taxon:** scientificName: Platyceruscaprea (De Geer, 1774); order: Coleoptera; family: Lucanidae; **Location:** country: ITALY; countryCode: IT; stateProvince: Lecco; locality: Monte Resegone; **Event:** year: 1944; month: 6; **Record Level:** institutionCode: Coll. Museo Civico di Storia Naturale di Milano
- **Type status:** Other material. **Occurrence:** recordedBy: leg. C. Brivio; individualCount: 1; sex: male; lifeStage: adult; occurrenceID: 68ADEFF3-59B3-5F38-833E-55CE08E0875C; **Taxon:** scientificName: Platyceruscaprea (De Geer, 1774); order: Coleoptera; family: Lucanidae; **Location:** country: ITALY; countryCode: IT; stateProvince: Lecco; municipality: Culmine di San Piero; **Event:** year: 2003; month: 5; day: 28; **Record Level:** institutionCode: Coll. Museo Civico di Storia Naturale di Milano
- **Type status:** Other material. **Occurrence:** recordedBy: leg. D. Pedersoli; individualCount: 1; lifeStage: adult; occurrenceID: 171EF461-A5F4-5FC1-B56C-F87A8E34352B; **Taxon:** scientificName: Platyceruscaprea (De Geer, 1774); order: Coleoptera; family: Lucanidae; **Location:** country: ITALY; countryCode: IT; stateProvince: Sondrio; municipality: Bema; locality: Alpe Vesenda bassa; verbatimElevation: 1460; decimalLatitude: 46.114167; decimalLongitude: 9.597788; geodeticDatum: WGS84; **Event:** year: 2007; month: 6; day: 14; **Record Level:** institutionCode: Coll. D. Pedersoli
- **Type status:** Other material. **Occurrence:** recordedBy: leg. R. Rossi; individualCount: 1; sex: female; lifeStage: adult; occurrenceID: 5BA9F66F-2809-5C5A-A841-697E04497E69; **Taxon:** scientificName: Platyceruscaprea (De Geer, 1774); order: Coleoptera; family: Lucanidae; **Location:** country: ITALY; countryCode: IT; stateProvince: Sondrio; municipality: Chiareggio; **Event:** year: 1959; month: 6; day: 27; **Record Level:** institutionCode: Coll. Museo Civico di Storia Naturale di Milano
- **Type status:** Other material. **Occurrence:** recordedBy: leg. D. Pedersoli; individualCount: 1; lifeStage: adult; occurrenceID: 2CB2F7E9-6ECD-55E7-A2BF-C101CAC4A9AB; **Taxon:** scientificName: Platyceruscaprea (De Geer, 1774); order: Coleoptera; family: Lucanidae; **Location:** country: ITALY; countryCode: IT; stateProvince: Sondrio; municipality: Villa di Tirano; locality: torbiera Pian del Gembro occ.; verbatimElevation: 1350; decimalLatitude: 46.164895; decimalLongitude: 10.160593; geodeticDatum: WGS84; **Event:** year: 2012; month: 5; day: 12; **Record Level:** institutionCode: Coll. D. Pedersoli
- **Type status:** Other material. **Occurrence:** individualCount: 1; sex: female; lifeStage: adult; occurrenceID: B28B593D-43A6-5D72-AF7A-6571FE007694; **Taxon:** scientificName: Platyceruscaprea (De Geer, 1774); order: Coleoptera; family: Lucanidae; **Location:** country: ITALY; countryCode: IT; stateProvince: Varese; municipality: Bedero Valcuvia; **Event:** year: 1971; month: 5; day: 9; **Record Level:** institutionCode: Coll. Museo Civico di Storia Naturale di Milano
- **Type status:** Other material. **Occurrence:** recordedBy: leg. G. Curletti; individualCount: 1; lifeStage: adult; occurrenceID: 576441F1-BF50-585D-80B2-DB405928D9EF; **Taxon:** scientificName: Platyceruscaprea (De Geer, 1774); order: Coleoptera; family: Lucanidae; **Location:** country: ITALY; countryCode: IT; stateProvince: Biella; locality: close to (cf.) Bocchetto; **Event:** year: 1977; month: 6; day: 24
- **Type status:** Other material. **Occurrence:** recordedBy: leg. Pescarolo; individualCount: 1; sex: male; lifeStage: adult; occurrenceID: A43E6BB1-9688-580D-81B2-3BDAB444407C; **Taxon:** scientificName: Platyceruscaprea (De Geer, 1774); order: Coleoptera; family: Lucanidae; **Location:** country: ITALY; countryCode: IT; stateProvince: Biella; locality: close to Bocchette SS., Val Sessera; **Event:** year: 1977; month: 6; day: 24; **Record Level:** institutionCode: Coll. Museo Civico di Storia Naturale di Carmagnola
- **Type status:** Other material. **Occurrence:** recordedBy: leg. G. Mariani; individualCount: 1; sex: female; lifeStage: adult; occurrenceID: E6F43177-F79B-530D-A9FD-2C12726B6CB4; **Taxon:** scientificName: Platyceruscaprea (De Geer, 1774); order: Coleoptera; family: Lucanidae; **Location:** country: ITALY; countryCode: IT; stateProvince: Biella; municipality: Biella; locality: Santuario di Oropa; verbatimElevation: 1200; **Event:** year: 1964; month: 5; day: 17; **Record Level:** institutionCode: Coll. Museo Civico di Storia Naturale di Milano
- **Type status:** Other material. **Occurrence:** recordedBy: leg. Callegari; individualCount: 1; sex: male; lifeStage: adult; occurrenceID: A4813B7C-38D5-52F1-A8FE-59DF06450E1D; **Taxon:** scientificName: Platyceruscaprea (De Geer, 1774); order: Coleoptera; family: Lucanidae; **Location:** country: ITALY; countryCode: IT; stateProvince: Biella; municipality: Bocchetto Sessera; verbatimElevation: 1200–1600; **Event:** year: 1993; month: 6; **Record Level:** institutionCode: Coll. Museo di Storia Naturale di Venezia (ex coll. F. Callegari)
- **Type status:** Other material. **Occurrence:** recordedBy: leg. Callegari; individualCount: 1; sex: male; lifeStage: adult; occurrenceID: 840E9588-1DAD-564F-B235-E9D8D8242E03; **Taxon:** scientificName: Platyceruscaprea (De Geer, 1774); order: Coleoptera; family: Lucanidae; **Location:** country: ITALY; countryCode: IT; stateProvince: Cuneo; municipality: Upega; verbatimElevation: 1300; **Event:** year: 1994; month: 6; day: 3-15 (mean 9); **Record Level:** institutionCode: Coll. Museo di Storia Naturale di Venezia (ex coll. F. Callegari)
- **Type status:** Other material. **Occurrence:** individualCount: 4; sex: 2 males, 2 females; lifeStage: adult; occurrenceID: 85F231AF-015D-56CB-84A2-173C066DFE04; **Taxon:** scientificName: Platyceruscaprea (De Geer, 1774); order: Coleoptera; family: Lucanidae; **Location:** country: ITALY; countryCode: IT; stateProvince: Torino; municipality: Andrate; locality: S. Giacomo; **Event:** year: 1972; **Record Level:** institutionCode: Coll. G. Taroni
- **Type status:** Other material. **Occurrence:** recordedBy: leg. Rochat; individualCount: 1; sex: male; lifeStage: adult; occurrenceID: 898CB0EF-5B43-5495-B6CF-7374EDF7A1CE; **Taxon:** scientificName: Platyceruscaprea (De Geer, 1774); order: Coleoptera; family: Lucanidae; **Location:** country: ITALY; countryCode: IT; stateProvince: Torino; municipality: Prali; locality: Val Germanasca; **Event:** year: 1974; month: 7; **Record Level:** institutionCode: Coll. Museo Civico di Storia Naturale di Milano
- **Type status:** Other material. **Occurrence:** recordedBy: leg. G. Curletti; individualCount: 1; lifeStage: adult; occurrenceID: 805A341F-60D5-5508-8495-4575116B1229; **Taxon:** scientificName: Platyceruscaprea (De Geer, 1774); order: Coleoptera; family: Lucanidae; **Location:** country: ITALY; countryCode: IT; stateProvince: Vercelli; municipality: -; locality: Val Cavaione; **Event:** year: 1985; month: 6; day: 10
- **Type status:** Other material. **Occurrence:** individualCount: 1; sex: male; lifeStage: adult; occurrenceID: 2BE97A52-48F2-5E60-8341-518DA6AB8824; **Taxon:** scientificName: Platyceruscaprea (De Geer, 1774); order: Coleoptera; family: Lucanidae; **Location:** country: ITALY; countryCode: IT; stateProvince: Vercelli; municipality: Alagna Valsesia; verbatimElevation: 1300; **Event:** year: 2012; month: 6; day: 16; **Record Level:** institutionCode: Coll. M. Zilioli
- **Type status:** Other material. **Occurrence:** recordedBy: leg. Pescarolo; individualCount: 1; sex: male; lifeStage: adult; occurrenceID: F371D58E-3997-53CC-A8C8-B2E7B5AAA7DA; **Taxon:** scientificName: Platyceruscaprea (De Geer, 1774); order: Coleoptera; family: Lucanidae; **Location:** country: ITALY; countryCode: IT; stateProvince: Vercelli; municipality: Boccioleto; locality: Val Sesia, Val Cavione; **Event:** year: 1985; month: 6; **Record Level:** institutionCode: Coll. Museo Civico di Storia Naturale di Carmagnola
- **Type status:** Other material. **Occurrence:** individualCount: 1; sex: male; lifeStage: adult; occurrenceID: 7BB9666B-D598-57CC-9460-855CF99F2132; **Taxon:** scientificName: Platyceruscaprea (De Geer, 1774); order: Coleoptera; family: Lucanidae; **Location:** country: ITALY; countryCode: IT; stateProvince: Vercelli; municipality: Carcoforo; locality: Val Sesia; verbatimElevation: 1300; **Event:** year: 2008; month: 6; day: 1; **Record Level:** institutionCode: Coll. M. Zilioli
- **Type status:** Other material. **Occurrence:** recordedBy: leg. G. Curletti; individualCount: 1; lifeStage: adult; occurrenceID: 060DB0A5-6166-5C25-B960-FC9217B4387E; **Taxon:** scientificName: Platyceruscaprea (De Geer, 1774); order: Coleoptera; family: Lucanidae; **Location:** country: ITALY; countryCode: IT; stateProvince: Vercelli; municipality: Fobello; locality: Val Sesia; **Event:** year: 1987; month: 6; day: 6
- **Type status:** Other material. **Occurrence:** recordedBy: leg. Pescarolo; individualCount: 1; sex: male; lifeStage: adult; occurrenceID: 8AE27F82-448C-5161-A8B6-6018DCFAAB3D; **Taxon:** scientificName: Platyceruscaprea (De Geer, 1774); order: Coleoptera; family: Lucanidae; **Location:** country: ITALY; countryCode: IT; stateProvince: Vercelli; municipality: Fobello; locality: Val Sesia, Fobello-Roi; **Event:** year: 1987; month: 6; day: 6; **Record Level:** institutionCode: Coll. Museo Civico di Storia Naturale di Carmagnola
- **Type status:** Other material. **Occurrence:** recordedBy: leg. Pescarolo; individualCount: 1; sex: female; lifeStage: adult; occurrenceID: 46871B75-484C-554B-AD96-CA189894E70C; **Taxon:** scientificName: Platyceruscaprea (De Geer, 1774); order: Coleoptera; family: Lucanidae; **Location:** country: ITALY; countryCode: IT; stateProvince: Vercelli; municipality: Pila; locality: Val Sesia; **Event:** year: 1984; month: 6; day: 10; **Record Level:** institutionCode: Coll. Museo Civico di Storia Naturale di Carmagnola
- **Type status:** Other material. **Occurrence:** recordedBy: leg. Pescarolo; individualCount: 2; sex: 1 male, 1 female; lifeStage: adult; occurrenceID: 335FFA8C-9E50-5958-B81F-E643AA74D751; **Taxon:** scientificName: Platyceruscaprea (De Geer, 1774); order: Coleoptera; family: Lucanidae; **Location:** country: ITALY; countryCode: IT; stateProvince: Vercelli; municipality: Pila; locality: Val Sesia, Lagone; **Event:** year: 1985; month: 6; day: 18; **Record Level:** institutionCode: Coll. Museo Civico di Storia Naturale di Carmagnola
- **Type status:** Other material. **Occurrence:** recordedBy: leg. G. Curletti; individualCount: 2; lifeStage: adult; occurrenceID: B8AE5BA1-56E6-563F-8078-8F352544A3AC; **Taxon:** scientificName: Platyceruscaprea (De Geer, 1774); order: Coleoptera; family: Lucanidae; **Location:** country: ITALY; countryCode: IT; stateProvince: Vercelli; municipality: Pila; locality: Val Sesia, Pila, Lagone; **Event:** year: 1985; month: 6; day: 18
- **Type status:** Other material. **Occurrence:** recordedBy: leg. G. Curletti; individualCount: 2; lifeStage: adult; occurrenceID: B050D978-D683-579C-AC76-E15578910BA9; **Taxon:** scientificName: Platyceruscaprea (De Geer, 1774); order: Coleoptera; family: Lucanidae; **Location:** country: ITALY; countryCode: IT; stateProvince: Vercelli; municipality: Pila; locality: Vallée Sessera; **Event:** year: 1989; month: 6; day: 11
- **Type status:** Other material. **Occurrence:** recordedBy: leg. G. Curletti; individualCount: 1; lifeStage: adult; occurrenceID: 58BE32E2-0F26-596D-A891-11A0AD38E76A; **Taxon:** scientificName: Platyceruscaprea (De Geer, 1774); order: Coleoptera; family: Lucanidae; **Location:** country: ITALY; countryCode: IT; stateProvince: Vercelli; municipality: Pila; **Event:** year: 1984; month: 6; day: 10
- **Type status:** Other material. **Occurrence:** individualCount: 1; lifeStage: adult; occurrenceID: 7B7D1FB4-5625-535C-AB30-CBF8DA8EAD54; **Taxon:** scientificName: Platyceruscaprea (De Geer, 1774); order: Coleoptera; family: Lucanidae; **Location:** country: ITALY; countryCode: IT; stateProvince: Vercelli; municipality: Riva Valdobbia; **Event:** year: 1990; month: 9; day: 8; **Record Level:** institutionCode: Coll. P. Leo
- **Type status:** Other material. **Occurrence:** recordedBy: leg. M. Zilioli; individualCount: 3; sex: males; lifeStage: adult; occurrenceID: 51CCE555-3948-5947-8AEA-A19FEFEE9B1F; **Taxon:** scientificName: Platyceruscaprea (De Geer, 1774); order: Coleoptera; family: Lucanidae; **Location:** country: ITALY; countryCode: IT; stateProvince: Vercelli; municipality: Scopello; locality: Alpe di Mera; verbatimElevation: 1350; **Event:** year: 2000; month: 5; day: 28; **Record Level:** institutionCode: Coll. M. Zilioli
- **Type status:** Other material. **Occurrence:** recordedBy: leg. Colombini; individualCount: 3; sex: males; lifeStage: adult; occurrenceID: 8638EAC4-75BE-5C68-A73F-D3DD4FDDD38D; **Taxon:** scientificName: Platyceruscaprea (De Geer, 1774); order: Coleoptera; family: Lucanidae; **Location:** country: ITALY; countryCode: IT; stateProvince: Vercelli; municipality: Scopello; locality: Alpe di Mera, Val Sesia; verbatimElevation: 1500; **Event:** year: 1995; month: 5; day: 28; **Record Level:** institutionCode: Coll. Museo Civico di Storia Naturale di Milano
- **Type status:** Other material. **Occurrence:** recordedBy: leg. Pescarolo; individualCount: 1; sex: female; lifeStage: adult; occurrenceID: D0869E56-B112-5B87-A0D9-CBE26C412774; **Taxon:** scientificName: Platyceruscaprea (De Geer, 1774); order: Coleoptera; family: Lucanidae; **Location:** country: ITALY; countryCode: IT; stateProvince: Vercelli; municipality: Scopello; locality: trail to Mera; verbatimElevation: 1300; **Event:** year: 1974; month: 6; day: 2; **Record Level:** institutionCode: Coll. Museo Civico di Storia Naturale di Carmagnola
- **Type status:** Other material. **Occurrence:** recordedBy: leg. G. Curletti; individualCount: 1; lifeStage: adult; occurrenceID: 39C8E4C7-BD42-50A9-9550-E0740E0C5C74; **Taxon:** scientificName: Platyceruscaprea (De Geer, 1774); order: Coleoptera; family: Lucanidae; **Location:** country: ITALY; countryCode: IT; stateProvince: Vercelli; municipality: Scopello; **Event:** year: 1984; month: 6; day: 2
- **Type status:** Other material. **Occurrence:** recordedBy: leg. Pescarolo; individualCount: 1; sex: male; lifeStage: adult; occurrenceID: 52DE6835-DA41-508C-B683-C5A974383D56; **Taxon:** scientificName: Platyceruscaprea (De Geer, 1774); order: Coleoptera; family: Lucanidae; **Location:** country: ITALY; countryCode: IT; stateProvince: Vercelli; municipality: Scopello; **Event:** year: 1994; month: 5; day: 15; **Record Level:** institutionCode: Coll. Museo Civico di Storia Naturale di Carmagnola
- **Type status:** Other material. **Occurrence:** recordedBy: leg. Nappini; individualCount: 1; lifeStage: adult; occurrenceID: 7C3CF0B1-DC4C-5CE7-9914-D307D1DC0B8F; **Taxon:** scientificName: Platyceruscaprea (De Geer, 1774); order: Coleoptera; family: Lucanidae; **Location:** country: ITALY; countryCode: IT; stateProvince: Bolzano; municipality: Campo di Trens; locality: close to Mules; verbatimElevation: 882; **Event:** year: 2017; month: 10; day: 28
- **Type status:** Other material. **Occurrence:** recordedBy: leg. Callegari; individualCount: 1; sex: female; lifeStage: adult; occurrenceID: 3FB09D57-0137-5268-8385-F70C90F0C310; **Taxon:** scientificName: Platyceruscaprea (De Geer, 1774); order: Coleoptera; family: Lucanidae; **Location:** country: ITALY; countryCode: IT; stateProvince: Bolzano; municipality: Dobbiaco; locality: Carbonin; verbatimElevation: 1400; **Event:** year: 1962; month: 8; **Record Level:** institutionCode: Coll. Museo di Storia Naturale di Venezia (ex coll. F. Callegari)
- **Type status:** Other material. **Occurrence:** recordedBy: leg. C. Alzona; individualCount: 2; sex: females; lifeStage: adult; occurrenceID: 367C28DD-08DB-5908-A327-460EDFD85D68; **Taxon:** scientificName: Platyceruscaprea (De Geer, 1774); order: Coleoptera; family: Lucanidae; **Location:** country: ITALY; countryCode: IT; stateProvince: Bolzano; municipality: Ortisei; locality: Val Gardena; **Event:** year: 1939; month: 7-8; **Record Level:** institutionCode: Coll. Museo Civico di Storia Naturale di Milano
- **Type status:** Other material. **Occurrence:** recordedBy: leg. Nappini; individualCount: 1; lifeStage: adult; occurrenceID: 2C57966E-7A53-567C-A245-45BB6F8011F8; **Taxon:** scientificName: Platyceruscaprea (De Geer, 1774); order: Coleoptera; family: Lucanidae; **Location:** country: ITALY; countryCode: IT; stateProvince: Bolzano; municipality: Val di Vizze; verbatimElevation: 1039; **Event:** year: 2018; month: 11; day: 18
- **Type status:** Other material. **Occurrence:** recordedBy: leg. M. Burlini; individualCount: 2; sex: 1 male, 1 female; lifeStage: adult; occurrenceID: 0F9956DA-D22D-57E0-BB51-D15D0FE02FA4; **Taxon:** scientificName: Platyceruscaprea (De Geer, 1774); order: Coleoptera; family: Lucanidae; **Location:** country: ITALY; countryCode: IT; stateProvince: Trento; locality: Monte Baldo; **Event:** year: 1929; month: 5; **Record Level:** institutionCode: Coll. Museo Civico di Storia Naturale di Milano
- **Type status:** Other material. **Occurrence:** recordedBy: leg. S. Guareschi; individualCount: 1; sex: female; lifeStage: adult; occurrenceID: 4110D446-2F9A-586C-9A00-E2FCC37D8909; **Taxon:** scientificName: Platyceruscaprea (De Geer, 1774); order: Coleoptera; family: Lucanidae; **Location:** country: ITALY; countryCode: IT; stateProvince: Trento; locality: Val Genova; **Event:** year: 1949; month: 6; day: 1; **Record Level:** institutionCode: Coll. Museo Civico di Storia Naturale di Milano
- **Type status:** Other material. **Occurrence:** recordedBy: leg. Callegari; individualCount: 3; sex: males; lifeStage: adult; occurrenceID: 4B62C90E-853D-5531-9D12-6AE0FD89A948; **Taxon:** scientificName: Platyceruscaprea (De Geer, 1774); order: Coleoptera; family: Lucanidae; **Location:** country: ITALY; countryCode: IT; stateProvince: Trento; locality: Val Genova; verbatimElevation: 1300; **Event:** year: 1963; month: 6; day: 13; **Record Level:** institutionCode: Coll. Museo di Storia Naturale di Venezia (ex coll. F. Callegari)
- **Type status:** Other material. **Occurrence:** individualCount: 1; sex: female; lifeStage: adult; occurrenceID: CADF380C-9B00-54B8-BB67-BA6C077F85E7; **Taxon:** scientificName: Platyceruscaprea (De Geer, 1774); order: Coleoptera; family: Lucanidae; **Location:** country: ITALY; countryCode: IT; stateProvince: Trento; municipality: Campiglio; locality: Val di Piera; **Event:** year: 1957; **Record Level:** institutionCode: Coll. G. Taroni
- **Type status:** Other material. **Occurrence:** recordedBy: leg. D. Pedersoli; individualCount: 1; lifeStage: adult; occurrenceID: 7A252C7A-EF0D-5346-80D9-B71439897994; **Taxon:** scientificName: Platyceruscaprea (De Geer, 1774); order: Coleoptera; family: Lucanidae; **Location:** country: ITALY; countryCode: IT; stateProvince: Trento; municipality: Ledro - Tiarno di Sopra; locality: trail from Lago d'Ampola to passo Tremalzo; verbatimElevation: 1150; decimalLatitude: 45.864382; decimalLongitude: 10.659762; geodeticDatum: WGS84; **Event:** year: 2008; month: 5; day: 17; **Record Level:** institutionCode: Coll. D. Pedersoli
- **Type status:** Other material. **Occurrence:** individualCount: 1; sex: female; lifeStage: adult; occurrenceID: A1131A03-C094-5FCC-8B8D-7CE1FBE071F0; **Taxon:** scientificName: Platyceruscaprea (De Geer, 1774); order: Coleoptera; family: Lucanidae; **Location:** country: ITALY; countryCode: IT; stateProvince: Trento; municipality: Sagron; **Event:** year: 1929; month: 7; **Record Level:** institutionCode: Coll. Museo di Storia Naturale di Venezia (coll. G. Bosa, Seminario Patriarcale di Venezia)
- **Type status:** Other material. **Occurrence:** recordedBy: leg. Chatel; individualCount: 1; sex: female; lifeStage: adult; occurrenceID: 8BE732E6-E483-5CDA-807C-252437005EB4; **Taxon:** scientificName: Platyceruscaprea (De Geer, 1774); order: Coleoptera; family: Lucanidae; **Location:** country: ITALY; countryCode: IT; stateProvince: Trento; municipality: Sella Giudicarie; locality: Lardaro; **Event:** year: 1959; month: 8; **Record Level:** institutionCode: Coll. Museo Civico di Storia Naturale di Milano
- **Type status:** Other material. **Occurrence:** recordedBy: leg. Petruzziello; individualCount: 1; lifeStage: adult; occurrenceID: 792509B1-FF55-5445-827F-AD9E603F06B6; **Taxon:** scientificName: Platyceruscaprea (De Geer, 1774); order: Coleoptera; family: Lucanidae; **Location:** country: ITALY; countryCode: IT; stateProvince: Trento; municipality: Tenno; locality: Lago di Tenno; verbatimElevation: 600; **Event:** year: 2005; month: 6; day: 10; **Record Level:** institutionCode: Coll. P. Leo
- **Type status:** Other material. **Occurrence:** recordedBy: leg. M. Burlini; individualCount: 1; sex: male; lifeStage: adult; occurrenceID: F481005A-E928-5B81-BFBA-060918D3BF66; **Taxon:** scientificName: Platyceruscaprea (De Geer, 1774); order: Coleoptera; family: Lucanidae; **Location:** country: ITALY; countryCode: IT; stateProvince: Trento; municipality: Trento; **Record Level:** institutionCode: Coll. Museo Civico di Storia Naturale di Milano
- **Type status:** Other material. **Occurrence:** individualCount: 1; sex: male; lifeStage: adult; occurrenceID: D7911914-22BF-51C8-9254-08FEF0E97AE7; **Taxon:** scientificName: Platyceruscaprea (De Geer, 1774); order: Coleoptera; family: Lucanidae; **Location:** country: ITALY; countryCode: IT; stateProvince: Lucca; municipality: Minucciano; locality: Alpi Apuane; **Event:** year: 1920; month: 6; day: 15; **Record Level:** institutionCode: Coll. Museo Civico di Storia Naturale di Milano
- **Type status:** Other material. **Occurrence:** recordedBy: leg. G. Cadamuro; individualCount: 1; sex: male; lifeStage: adult; occurrenceID: E8B8C7CE-0C58-571E-AD54-D19641B3C29E; **Taxon:** scientificName: Platyceruscaprea (De Geer, 1774); order: Coleoptera; family: Lucanidae; **Location:** country: ITALY; countryCode: IT; stateProvince: Belluno; locality: "alta Val Serais"; **Event:** year: 1955; month: 7; **Record Level:** institutionCode: Coll. Museo di Storia Naturale di Venezia
- **Type status:** Other material. **Occurrence:** individualCount: 1; sex: female; lifeStage: adult; occurrenceID: 096E9F50-09CB-58B8-866D-E951A9A8A09A; **Taxon:** scientificName: Platyceruscaprea (De Geer, 1774); order: Coleoptera; family: Lucanidae; **Location:** country: ITALY; countryCode: IT; stateProvince: Belluno; locality: California; **Event:** year: 1931; month: 6; day: 18; **Record Level:** institutionCode: Coll. Museo di Storia Naturale di Venezia (coll. G. Bosa, Seminario Patriarcale di Venezia)
- **Type status:** Other material. **Occurrence:** individualCount: 2; sex: males; lifeStage: adult; occurrenceID: 2CB9F89C-8B38-5639-98BD-D49D94EA66EC; **Taxon:** scientificName: Platyceruscaprea (De Geer, 1774); order: Coleoptera; family: Lucanidae; **Location:** country: ITALY; countryCode: IT; stateProvince: Belluno; locality: Cansiglio; **Event:** year: 1957; month: 7; day: 1; **Record Level:** institutionCode: Coll. Museo Civico di Storia Naturale di Milano
- **Type status:** Other material. **Occurrence:** recordedBy: leg. G. Cadamuro; individualCount: 1; sex: female; lifeStage: adult; occurrenceID: BD4D16C8-E607-5460-AFB6-0CC867983329; **Taxon:** scientificName: Platyceruscaprea (De Geer, 1774); order: Coleoptera; family: Lucanidae; **Location:** country: ITALY; countryCode: IT; stateProvince: Belluno; locality: Cansiglio; **Event:** year: 1955; month: 7; **Record Level:** institutionCode: Coll. Museo di Storia Naturale di Venezia
- **Type status:** Other material. **Occurrence:** recordedBy: leg. G. Agazzi; individualCount: 1; sex: male; lifeStage: adult; occurrenceID: 37CEEDD4-0046-564D-9C31-89761C1D9A49; **Taxon:** scientificName: Platyceruscaprea (De Geer, 1774); order: Coleoptera; family: Lucanidae; **Location:** country: ITALY; countryCode: IT; stateProvince: Belluno; locality: Val Resia; **Event:** year: 1956; month: 5; day: 3; **Record Level:** institutionCode: Coll. Museo di Storia Naturale di Venezia (ex coll. G. Agazzi)
- **Type status:** Other material. **Occurrence:** recordedBy: leg. F. Giannone; individualCount: 1; sex: female; lifeStage: adult; occurrenceID: 9475762D-2A42-59B7-9301-7F653E580F41; **Taxon:** scientificName: Platyceruscaprea (De Geer, 1774); order: Coleoptera; family: Lucanidae; **Location:** country: ITALY; countryCode: IT; stateProvince: Belluno; municipality: Canale d'Agordo; locality: Canale d'Agordo, Val Gares; decimalLatitude: 46.357721; decimalLongitude: 11.927711; geodeticDatum: WGS84; **Event:** year: 1984; month: 7; day: 23; **Record Level:** institutionCode: Coll. D. Scaccini
- **Type status:** Other material. **Occurrence:** recordedBy: leg. F. Giannone; individualCount: 1; sex: female; lifeStage: adult; occurrenceID: 6DCBAB7E-FD07-5D6E-820D-828DD07BB4D3; **Taxon:** scientificName: Platyceruscaprea (De Geer, 1774); order: Coleoptera; family: Lucanidae; **Location:** country: ITALY; countryCode: IT; stateProvince: Belluno; municipality: Canale d'Agordo; locality: Canale d'Agordo, Val Gares; decimalLatitude: 46.357616; decimalLongitude: 11.924225; geodeticDatum: WGS84; **Event:** year: 1994; month: 6; day: 19; **Record Level:** institutionCode: Coll. D. Scaccini
- **Type status:** Other material. **Occurrence:** recordedBy: leg. L. Bonometto; individualCount: 1; sex: female; lifeStage: adult; occurrenceID: AD2B4438-113C-5A98-9769-398ECA3B824C; **Taxon:** scientificName: Platyceruscaprea (De Geer, 1774); order: Coleoptera; family: Lucanidae; **Location:** country: ITALY; countryCode: IT; stateProvince: Belluno; municipality: Lorenzago; locality: Val dell'Orso; **Event:** year: 1968; month: 7; **Record Level:** institutionCode: Coll. Museo di Storia Naturale di Venezia
- **Type status:** Other material. **Occurrence:** recordedBy: leg. P. Fontolan; individualCount: 1; sex: female; lifeStage: adult; occurrenceID: 25F2CBCF-C16C-5385-9B17-C920BFF7E7A3; **Taxon:** scientificName: Platyceruscaprea (De Geer, 1774); order: Coleoptera; family: Lucanidae; **Location:** country: ITALY; countryCode: IT; stateProvince: Belluno; municipality: San Vito di Cadore; **Event:** year: 1955; month: 7; day: 4-10 (mean 7); **Record Level:** institutionCode: Coll. Museo di Storia Naturale di Venezia (ex coll. G. Agazzi)
- **Type status:** Other material. **Occurrence:** recordedBy: leg. D. Scaccini; individualCount: 1; lifeStage: adult; occurrenceID: 6F8B5538-48EF-58A5-BBC8-6694B3E108E5; **Taxon:** scientificName: Platyceruscaprea (De Geer, 1774); order: Coleoptera; family: Lucanidae; **Location:** country: ITALY; countryCode: IT; stateProvince: Vicenza; municipality: Asiago; locality: close to Forte Interrotto; verbatimElevation: 1369; decimalLatitude: 45.977543; decimalLongitude: 11.177685; geodeticDatum: WGS84; **Event:** year: 2023; month: 1; day: 15
- **Type status:** Other material. **Occurrence:** recordedBy: leg. D. Scaccini; individualCount: 1; lifeStage: adult; occurrenceID: 658ADECC-DB46-5C0E-A031-B8A96F5FB856; **Taxon:** scientificName: Platyceruscaprea (De Geer, 1774); order: Coleoptera; family: Lucanidae; **Location:** country: ITALY; countryCode: IT; stateProvince: Vicenza; municipality: Asiago; locality: trail to Forte Interrotto; verbatimElevation: 1259; decimalLatitude: 45.893206; decimalLongitude: 11.482112; geodeticDatum: WGS84; **Event:** year: 2023; month: 1; day: 15
- **Type status:** Other material. **Occurrence:** recordedBy: leg. D. Scaccini; individualCount: 2; sex: 1 male, 1 female; lifeStage: adult; occurrenceID: A870B1B4-A4A1-592D-B26C-B3C4FD00CE49; **Taxon:** scientificName: Platyceruscaprea (De Geer, 1774); order: Coleoptera; family: Lucanidae; **Location:** country: ITALY; countryCode: IT; stateProvince: Vicenza; municipality: Tresché Conca (Asiago); verbatimElevation: 1104; decimalLatitude: 45.828672; decimalLongitude: 11.441061; geodeticDatum: WGS84; **Event:** year: 2019; month: 3; day: 30; **Record Level:** institutionCode: Coll. D. Scaccini
- **Type status:** Other material. **Occurrence:** recordedBy: leg. G. Nardi & M. Bardiani; individualCount: 2; lifeStage: adult; occurrenceID: 04554D5C-D885-57B4-A675-61B0110A0819; **Taxon:** scientificName: Platyceruscaprea (De Geer, 1774); order: Coleoptera; family: Lucanidae; **Location:** country: ITALY; countryCode: IT; stateProvince: Parma; county: Corniglio; municipality: Lagdei, Lago di Guadine, Riserva Guadine Pradaccio; locality: Monte Ruella; verbatimElevation: 1362; decimalLatitude: 44.397441; decimalLongitude: 10.018822; geodeticDatum: WGS84; **Event:** year: 2008; month: 5; day: 23
- **Type status:** Other material. **Occurrence:** recordedBy: leg. G. Nardi & M. Bardiani; individualCount: 1; lifeStage: adult; occurrenceID: 07C789E6-0DFB-5064-9A7E-E715824B37C3; **Taxon:** scientificName: Platyceruscaprea (De Geer, 1774); order: Coleoptera; family: Lucanidae; **Location:** country: ITALY; countryCode: IT; stateProvince: Parma; county: Corniglio; municipality: Lagdei, torbiera Roccabianca, Riserva Guadine Pradaccio; locality: Rocca Santa Maria; verbatimElevation: 1440; decimalLatitude: 44.393245; decimalLongitude: 10.021901; geodeticDatum: WGS84; **Event:** year: 2008; month: 5; day: 23
- **Type status:** Other material. **Occurrence:** recordedBy: leg. G.E. Feltrin; individualCount: 1; lifeStage: adult; occurrenceID: 58FDAC88-912E-5CF5-88F9-953E351A801A; **Taxon:** scientificName: Platyceruscaprea (De Geer, 1774); order: Coleoptera; family: Lucanidae; **Location:** country: ITALY; countryCode: IT; stateProvince: Belluno; county: Lorenzago di Cadore; municipality: Valdescura; verbatimElevation: 1149; decimalLatitude: 46.471389; decimalLongitude: 12.472222; geodeticDatum: WGS84; **Event:** year: 2013; month: 5-6; day: 30 (5) - 15 (6)
- **Type status:** Other material. **Occurrence:** recordedBy: leg. F. Bona; individualCount: 3; lifeStage: adult; occurrenceID: 8DC2714F-D38E-587E-BA6F-208A3F336620; **Taxon:** scientificName: Platyceruscaprea (De Geer, 1774); order: Coleoptera; family: Lucanidae; **Location:** country: ITALY; countryCode: IT; stateProvince: Treviso; county: Fregona; municipality: Bosco Cansiglio; locality: Monte Sila, Botte Donato, Strada delle Vette; verbatimElevation: 1340; decimalLatitude: 46.041767; decimalLongitude: 12.375483; geodeticDatum: WGS84; **Event:** year: 2013; month: 6; day: 11-24
- **Type status:** Other material. **Occurrence:** recordedBy: leg. D. Scaccini; individualCount: 3; lifeStage: larva; occurrenceID: A8C98AE5-F5B1-58FD-8AA1-8FF63475793E; **Taxon:** scientificName: Platyceruscaprea (De Geer, 1774); order: Coleoptera; family: Lucanidae; **Location:** country: ITALY; countryCode: IT; stateProvince: Bergamo; county: Carona; municipality: Site 3; locality: Monte Cervati; verbatimElevation: 1194; decimalLatitude: 46.024506; decimalLongitude: 9.794025; geodeticDatum: WGS84; **Event:** year: 2024; month: 12; day: 29
- **Type status:** Other material. **Occurrence:** recordedBy: leg. F. Cussigh; individualCount: 1; sex: female; lifeStage: adult; occurrenceID: 5E8E53C8-8068-504E-A3EF-317D5E0E20A7; **Taxon:** scientificName: Platyceruscaprea (De Geer, 1774); order: Coleoptera; family: Lucanidae; **Location:** country: ITALY; countryCode: IT; stateProvince: Vicenza; municipality: Sentiero Monte Meatta (Asiago); locality: Campigna; **Event:** year: 1982; month: 6; day: 27; **Record Level:** institutionCode: Coll. Museo Naturalistico Archeologico, Vicenza (ex. Coll. F. Cussigh); collectionCode: Collection code: mnav-ent.fc-09711
- **Type status:** Other material. **Occurrence:** recordedBy: leg. F. Cussigh; individualCount: 1; sex: male; lifeStage: adult; occurrenceID: 643C0BA6-C582-52D5-87F6-BDA849EAC3C8; **Taxon:** scientificName: Platyceruscaprea (De Geer, 1774); order: Coleoptera; family: Lucanidae; **Location:** country: ITALY; countryCode: IT; stateProvince: Vicenza; county: Cogollo del Cengio; municipality: zona Monte Cengio (Asiago); locality: Foresta di Campigna; **Event:** year: 1979; month: 6; day: 10; **Record Level:** institutionCode: Coll. Museo Naturalistico Archeologico, Vicenza (ex. Coll. F. Cussigh); collectionCode: Collection code: mnav-ent.fc-09709
- **Type status:** Other material. **Occurrence:** recordedBy: leg. F. Cussigh; individualCount: 1; sex: male; lifeStage: adult; occurrenceID: CDC9B83B-076E-565E-98A4-47F0B369A7B7; **Taxon:** scientificName: Platyceruscaprea (De Geer, 1774); order: Coleoptera; family: Lucanidae; **Location:** country: ITALY; countryCode: IT; stateProvince: Vicenza; county: Recoaro; municipality: Fonti Lora; locality: Lago Santo; **Event:** year: 1986; month: 5; day: 24; **Record Level:** institutionCode: Coll. Museo Naturalistico Archeologico, Vicenza (ex. Coll. F. Cussigh); collectionCode: Collection code: mnav-ent.fc-09706
- **Type status:** Other material. **Occurrence:** recordedBy: leg. F. Cussigh; individualCount: 2; sex: 1 male, 1 female; lifeStage: adult; occurrenceID: B18E7C64-715B-5753-8320-B2CED048E825; **Taxon:** scientificName: Platyceruscaprea (De Geer, 1774); order: Coleoptera; family: Lucanidae; **Location:** country: ITALY; countryCode: IT; stateProvince: Vicenza; county: Recoaro; municipality: Recoaro 1000 - Gabiola; locality: Groppo delle Ali; **Event:** year: 1989; month: 6; day: 18; **Record Level:** institutionCode: Coll. Museo Naturalistico Archeologico, Vicenza (ex. Coll. F. Cussigh); collectionCode: Collection codes: mnav-ent.fc-09708, mnav-ent.fc-09712
- **Type status:** Other material. **Occurrence:** recordedBy: leg. F. Cussigh; individualCount: 1; sex: male; lifeStage: adult; occurrenceID: F48CB4B2-B619-5CF1-B588-EBE46F519BE1; **Taxon:** scientificName: Platyceruscaprea (De Geer, 1774); order: Coleoptera; family: Lucanidae; **Location:** country: ITALY; countryCode: IT; stateProvince: Vicenza; county: Recoaro Terme; municipality: Campogrosso (Asiago); locality: Bosco del Cansiglio; **Event:** year: 1984; month: 6; day: 16; **Record Level:** institutionCode: Coll. Museo Naturalistico Archeologico, Vicenza (ex. Coll. F. Cussigh); collectionCode: Collection code: mnav-ent.fc-09710
- **Type status:** Other material. **Occurrence:** recordedBy: leg. F. Cussigh; individualCount: 2; sex: 1 male, 1 female; lifeStage: adult; occurrenceID: DFF25C98-13CA-5B93-A019-45EAD884BFA1; **Taxon:** scientificName: Platyceruscaprea (De Geer, 1774); order: Coleoptera; family: Lucanidae; **Location:** country: ITALY; countryCode: IT; stateProvince: Vicenza; county: Roana; municipality: Val d'Assa; locality: Alpi Giulie; verbatimElevation: 1150-1200; **Event:** year: 1985; month: 6; day: 30; **Record Level:** institutionCode: Coll. Museo Naturalistico Archeologico, Vicenza (ex. Coll. F. Cussigh); collectionCode: Collection codes: mnav-ent.fc-09707, mnav-ent.fc-09713
- **Type status:** Other material. **Occurrence:** recordedBy: leg. S. Biondi; individualCount: 1; sex: female; lifeStage: adult; occurrenceID: 5CC7B82F-0B2A-5697-A54B-77538AE04CA8; **Taxon:** scientificName: Platyceruscaprea (De Geer, 1774); order: Coleoptera; family: Lucanidae; **Location:** country: ITALY; countryCode: IT; stateProvince: Vicenza; county: Roana; municipality: Mezzaselva Roana (Asiago, 7 Comuni); locality: Cernkerla; **Event:** year: 2011; month: 6; day: 22; **Record Level:** institutionCode: Coll. S. Biondi

#### Distribution

**Figure 4.**
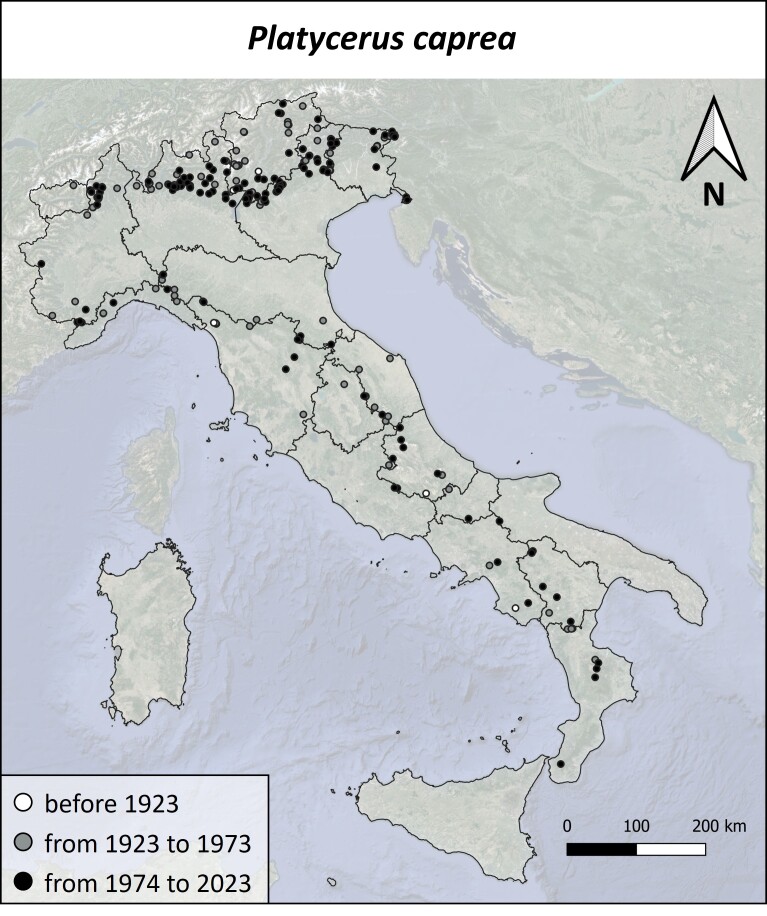
Italian distribution of *Platyceruscaprea*, featuring findings from different collection periods. Updated to 31 December 2023.

The examination of the available material yielded 114 new distributional records, representing a surge of over 70% compared to the existing literature. *Platyceruscaprea* is, thus, recorded for 271 Italian localities, but it is absent from Sicily and Sardinia (Fig. 4). This species has been mostly observed in the Alps, while its records in peninsular Italy are gradually diminishing towards the south. Particularly, it can be seen that occurrences are located almost exclusively in correspondence with foothill and mountainous areas, for both historical and original data. The temporal distribution of *P.caprea* occurrences is as follows: 11 observations (4.1%) were collected prior to 1923, 101 (37.3%) were collected between 1923 and 1973 and 159 (58.7%) from 1973 to present (Suppl. material 1).

#### Ecology

**Figure 5.**
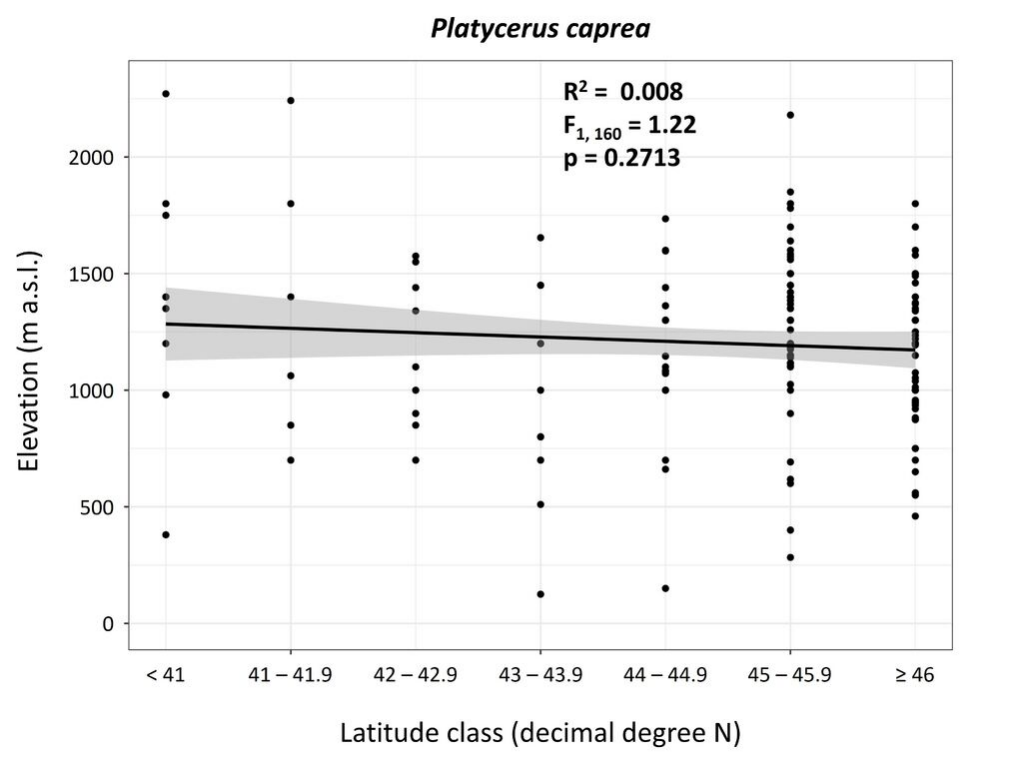
Linear regression on *Platyceruscaprea* finding locations for the relationship between latitude and elevation in Italy. F statistic, R^2^ and p values are reported in the graph.

In sites where this information is known (n = 162), the mean (± st. dev.) elevation was 1202.38 ± 370.30 m a.s.l. and it ranged from 125 to 2271 m a.s.l. Despite having a general negative trend, the statistical analysis run on the latitude-elevation relationship did not show significant results for the linear (Fig. 5) nor the quadratic regression (F_2, 159_ = 0.982, p = 0.3768). The activity period of *P.caprea* adults appears to predominantly span the months of May, June and July, but details on these aspects are often scattered or untraceable.

### Platycerus caraboides caraboides

(Linnaeus, 1758)

B0FCC9D8-146E-5177-B578-F595180673C5

#### Materials

- **Type status:** Other material. **Occurrence:** recordedBy: leg. G. Giovagnoli; individualCount: 1; sex: male; lifeStage: adult; occurrenceID: F3D1971F-A280-5394-9BDE-7A69C4624B04; **Taxon:** scientificName: Platyceruscaraboides (Linnaeus, 1758); order: Coleoptera; family: Lucanidae; **Location:** country: Italy; countryCode: IT; stateProvince: L'Aquila; county: Pescosancostanzo; locality: Bosco di Sant'Antonio; **Event:** year: 2015; month: 5; day: 30; **Record Level:** collectionID: Coll. G. Giovagnoli
- **Type status:** Other material. **Occurrence:** recordedBy: leg. F. Presutto; individualCount: 1; sex: male; lifeStage: adult; occurrenceID: 1FA94261-0822-5A2B-BABA-7AF08B102625; **Taxon:** scientificName: Platyceruscaraboides (Linnaeus, 1758); order: Coleoptera; family: Lucanidae; **Location:** country: Italy; countryCode: IT; stateProvince: Pescara; county: Salle; locality: Montagne Morrone; verbatimElevation: 496; **Event:** year: 1998; month: 6; day: 21; **Record Level:** collectionID: Coll. L. Bartolozzi
- **Type status:** Other material. **Occurrence:** recordedBy: leg. F. Presutto; individualCount: 1; sex: male; lifeStage: adult; occurrenceID: A5A5A3CB-44F2-53B7-965C-8E4D8328DEA1; **Taxon:** scientificName: Platyceruscaraboides (Linnaeus, 1758); order: Coleoptera; family: Lucanidae; **Location:** country: Italy; countryCode: IT; stateProvince: Pescara; county: Salle; locality: Montagne Morrone; verbatimElevation: 1036; **Event:** year: 1998; month: 7; day: 19; **Record Level:** collectionID: Coll. L. Bartolozzi
- **Type status:** Other material. **Occurrence:** recordedBy: leg. F. Presutto; individualCount: 2; sex: 1 male, 1 female; lifeStage: adult; occurrenceID: 3E756CD8-BFC5-505D-9FC3-7360E2B8C806; **Taxon:** scientificName: Platyceruscaraboides (Linnaeus, 1758); order: Coleoptera; family: Lucanidae; **Location:** country: Italy; countryCode: IT; stateProvince: Pescara; county: Tocco da Casauria; locality: Montagne Morrone; verbatimElevation: 1270; **Event:** year: 1998; month: 8; day: 11; **Record Level:** collectionID: Coll. L. Bartolozzi
- **Type status:** Other material. **Occurrence:** recordedBy: leg. Fam. Penna; individualCount: 1; lifeStage: adult; occurrenceID: 6E5A4E19-8704-5078-9D31-3C2D688DD1A3; **Taxon:** scientificName: Platyceruscaraboides (Linnaeus, 1758); order: Coleoptera; family: Lucanidae; **Location:** country: Italy; countryCode: IT; stateProvince: Aosta; county: Courmayeur; locality: Parco giochi Courmayeur; verbatimElevation: 1250; **Event:** year: 2009; month: 6; day: 21; **Record Level:** collectionID: Coll. Ugo Bosia
- **Type status:** Other material. **Occurrence:** recordedBy: leg. Schatzmayr-Koch; individualCount: 2; sex: female; lifeStage: adult; occurrenceID: 237DDBFD-82F9-50F2-BE8C-B36B43D341B3; **Taxon:** scientificName: Platyceruscaraboides (Linnaeus, 1758); order: Coleoptera; family: Lucanidae; **Location:** country: Italy; countryCode: IT; stateProvince: Cosenza; county: Acri; locality: Duglia; **Event:** year: 1933; month: 7; day: 17; **Record Level:** collectionID: Coll. Museo Civico di Storia Naturale di Milano
- **Type status:** Other material. **Occurrence:** recordedBy: leg. M. Valle; individualCount: 1; lifeStage: adult; occurrenceID: 12E97F03-AB79-53E2-BA0F-818803F2A84F; **Taxon:** scientificName: Platyceruscaraboides (Linnaeus, 1758); order: Coleoptera; family: Lucanidae; **Location:** country: Italy; countryCode: IT; stateProvince: Cosenza; county: Fagnano Castello; locality: lago Trifoglietti; verbatimElevation: 1068; decimalLatitude: 39.5489; decimalLongitude: 16.0229; geodeticDatum: WGS84; **Event:** year: 2019; month: 5; day: 20; **Record Level:** collectionID: Coll. Museo "E. Caffi", Bergamo
- **Type status:** Other material. **Occurrence:** individualCount: 1; lifeStage: adult; occurrenceID: 66268F73-5182-5310-AB3E-21D20DB1409C; **Taxon:** scientificName: Platyceruscaraboides (Linnaeus, 1758); order: Coleoptera; family: Lucanidae; **Location:** country: Italy; countryCode: IT; stateProvince: Salerno; county: Monte Cervati; verbatimElevation: 1800; **Event:** year: 2018; month: 5; day: 26; **Record Level:** collectionID: Coll. A. Carlin
- **Type status:** Other material. **Occurrence:** recordedBy: leg. S. Nappini; individualCount: 7; lifeStage: adult; occurrenceID: AA944A20-7F3B-5186-90FE-E43AAF01CE45; **Taxon:** scientificName: Platyceruscaraboides (Linnaeus, 1758); order: Coleoptera; family: Lucanidae; **Location:** country: Italy; countryCode: IT; stateProvince: Bologna; county: Granaglione; locality: Monte Cavallo; verbatimElevation: 1195; **Event:** year: 2013; month: 4; day: 17
- **Type status:** Other material. **Occurrence:** recordedBy: leg. F. Faggi; individualCount: 1; lifeStage: adult; occurrenceID: 36CE9087-90F0-50C5-B1F0-8DD76DBD07CF; **Taxon:** scientificName: Platyceruscaraboides (Linnaeus, 1758); order: Coleoptera; family: Lucanidae; **Location:** country: Italy; countryCode: IT; stateProvince: Bologna; county: Lizzano in Belvedere; locality: Corno alle Scale; **Event:** year: 2005; month: 5; day: 29
- **Type status:** Other material. **Occurrence:** recordedBy: photo by S. Magagnoli; individualCount: 1; sex: male; lifeStage: adult; occurrenceID: 1DCE9B02-57B1-5E5B-9511-D847E044B2EA; **Taxon:** scientificName: Platyceruscaraboides (Linnaeus, 1758); order: Coleoptera; family: Lucanidae; **Location:** country: Italy; countryCode: IT; stateProvince: Forlì-Cesena; county: Campigna; **Event:** year: 2018; month: 4; day: 30
- **Type status:** Other material. **Occurrence:** recordedBy: leg. M. Tomassetti; individualCount: 1; sex: female; lifeStage: adult; occurrenceID: 401EC097-9A51-5B78-BC49-D8E0CEE9018E; **Taxon:** scientificName: Platyceruscaraboides (Linnaeus, 1758); order: Coleoptera; family: Lucanidae; **Location:** country: Italy; countryCode: IT; stateProvince: Forlì-Cesena; county: Campigna; **Event:** year: 1950; month: 7; day: 25; **Record Level:** collectionID: Coll. Museo Civico di Storia Naturale di Milano
- **Type status:** Other material. **Occurrence:** recordedBy: leg. A. Bastianini & S. Nappini; individualCount: 3; lifeStage: adult; occurrenceID: 7538E305-2EEB-5AE0-AF5C-51A145ABBD8A; **Taxon:** scientificName: Platyceruscaraboides (Linnaeus, 1758); order: Coleoptera; family: Lucanidae; **Location:** country: Italy; countryCode: IT; stateProvince: Forlì-Cesena; county: Premilcuore; locality: Foreste Casentinesi; verbatimElevation: 1369; **Event:** year: 2013; month: 4; day: 26
- **Type status:** Other material. **Occurrence:** recordedBy: leg. Callegari; individualCount: 1; sex: male; lifeStage: adult; occurrenceID: AC67C652-0AE5-553E-A4A5-FD2D1D2C929A; **Taxon:** scientificName: Platyceruscaraboides (Linnaeus, 1758); order: Coleoptera; family: Lucanidae; **Location:** country: Italy; countryCode: IT; stateProvince: Forlì-Cesena; county: San Benedetto in Alpe; **Event:** year: 1986; month: 5; day: 1; **Record Level:** collectionID: Coll. Museo di Storia Naturale di Venezia
- **Type status:** Other material. **Occurrence:** recordedBy: leg. Callegari; individualCount: 1; sex: male; lifeStage: adult; occurrenceID: 3770EEAC-4EE1-565A-9A03-5C63824D5719; **Taxon:** scientificName: Platyceruscaraboides (Linnaeus, 1758); order: Coleoptera; family: Lucanidae; **Location:** country: Italy; countryCode: IT; stateProvince: Forlì-Cesena; county: Santa Sofia; locality: close to Campigna (below); **Event:** year: 1988; month: 5; day: 24; **Record Level:** collectionID: Coll. Museo di Storia Naturale di Venezia
- **Type status:** Other material. **Occurrence:** recordedBy: leg. Callegari; individualCount: 1; sex: male; lifeStage: adult; occurrenceID: 52350A72-A7C6-5BFE-AB9F-1D97FAAC8A1A; **Taxon:** scientificName: Platyceruscaraboides (Linnaeus, 1758); order: Coleoptera; family: Lucanidae; **Location:** country: Italy; countryCode: IT; stateProvince: Forlì-Cesena; county: Sarsina; locality: Sarsina Montirolo; **Event:** year: 1986; month: 6; day: 10; **Record Level:** collectionID: Coll. Museo di Storia Naturale di Venezia
- **Type status:** Other material. **Occurrence:** recordedBy: leg. S. Nappini; individualCount: 4; lifeStage: adult; occurrenceID: 9B87AC27-7E4E-5E36-A745-83D0D8AAD870; **Taxon:** scientificName: Platyceruscaraboides (Linnaeus, 1758); order: Coleoptera; family: Lucanidae; **Location:** country: Italy; countryCode: IT; stateProvince: Modena; county: Sestola; locality: Monte Cimone; verbatimElevation: 1325; **Event:** year: 2015; month: 5; day: 6
- **Type status:** Other material. **Occurrence:** recordedBy: leg. Baldini; individualCount: 1; sex: male; lifeStage: adult; occurrenceID: C22D2954-9C4D-51C6-A6CD-F1684487AD68; **Taxon:** scientificName: Platyceruscaraboides (Linnaeus, 1758); order: Coleoptera; family: Lucanidae; **Location:** country: Italy; countryCode: IT; stateProvince: Piacenza; county: Bobbio; locality: Monte Penice; **Event:** year: 1959; month: 5; day: 28; **Record Level:** collectionID: Coll. Museo Civico di Storia Naturale di Milano
- **Type status:** Other material. **Occurrence:** recordedBy: leg. Ragozzino; individualCount: 1; sex: female; lifeStage: adult; occurrenceID: 5581F6A5-829B-56B0-BDF7-ACC9393946AC; **Taxon:** scientificName: Platyceruscaraboides (Linnaeus, 1758); order: Coleoptera; family: Lucanidae; **Location:** country: Italy; countryCode: IT; stateProvince: Piacenza; county: Ferriere; locality: Ferriere (nearby); **Event:** year: 1969; month: 8; day: 8; **Record Level:** collectionID: Coll. Museo Civico di Storia Naturale di Milano
- **Type status:** Other material. **Occurrence:** recordedBy: leg. Callegari; individualCount: 1; sex: male; lifeStage: adult; occurrenceID: 4F26B8ED-8371-59E6-80E6-FA93927D7AD5; **Taxon:** scientificName: Platyceruscaraboides (Linnaeus, 1758); order: Coleoptera; family: Lucanidae; **Location:** country: Italy; countryCode: IT; stateProvince: Pordenone; county: -; locality: Monte Chiarandeit; verbatimElevation: 600–800; **Event:** year: 1995; month: 5; day: 21–25; **Record Level:** collectionID: Coll. Museo di Storia Naturale di Venezia
- **Type status:** Other material. **Occurrence:** recordedBy: leg. C. Baseotto; individualCount: 1; lifeStage: adult; occurrenceID: EFDAC666-9A59-5169-8BC2-CAC9D583AAB0; **Taxon:** scientificName: Platyceruscaraboides (Linnaeus, 1758); order: Coleoptera; family: Lucanidae; **Location:** country: Italy; countryCode: IT; stateProvince: Pordenone; county: Barcis; locality: Foresta del Prescudin; verbatimElevation: 506; **Event:** year: 2004; month: 5; day: 19; **Record Level:** collectionID: Coll. G. Stefani
- **Type status:** Other material. **Occurrence:** recordedBy: leg. G. Stefani; individualCount: 1; lifeStage: adult; occurrenceID: C00F3A14-B722-5B38-98D1-52FCFAC19944; **Taxon:** scientificName: Platyceruscaraboides (Linnaeus, 1758); order: Coleoptera; family: Lucanidae; **Location:** country: Italy; countryCode: IT; stateProvince: Pordenone; county: Frisanco; locality: Casasola; verbatimElevation: 450; **Event:** year: 2023; month: 5; day: 1; **Record Level:** collectionID: Coll. G. Stefani
- **Type status:** Other material. **Occurrence:** recordedBy: leg. Callegari; individualCount: 1; sex: male; lifeStage: adult; occurrenceID: 83C66907-2E97-53BF-B13F-41B744B9D59E; **Taxon:** scientificName: Platyceruscaraboides (Linnaeus, 1758); order: Coleoptera; family: Lucanidae; **Location:** country: Italy; countryCode: IT; stateProvince: Pordenone; county: Poffabro; verbatimElevation: 400–800; **Event:** year: 1995; month: 5; day: 8–26; **Record Level:** collectionID: Coll. Museo di Storia Naturale di Venezia
- **Type status:** Other material. **Occurrence:** recordedBy: leg. G. Delirio; individualCount: 1; lifeStage: adult; occurrenceID: 869E8F5D-A6DF-588D-815A-08B97F0A36EA; **Taxon:** scientificName: Platyceruscaraboides (Linnaeus, 1758); order: Coleoptera; family: Lucanidae; **Location:** country: Italy; countryCode: IT; stateProvince: Udine; county: Alta Val Torre; locality: Lusevera; **Event:** year: 1994; month: 6; day: 14; **Record Level:** collectionID: Coll. D. Sechi
- **Type status:** Other material. **Occurrence:** individualCount: 1; lifeStage: adult; occurrenceID: 082FA6C6-DEBA-548F-A744-07E6400DFA39; **Taxon:** scientificName: Platyceruscaraboides (Linnaeus, 1758); order: Coleoptera; family: Lucanidae; **Location:** country: Italy; countryCode: IT; stateProvince: Udine; county: Passo Tanamea; locality: trail Monte Maggiore; verbatimElevation: 900; **Event:** year: 2000; month: 5; day: 28; **Record Level:** collectionID: Coll. A. Carlin
- **Type status:** Other material. **Occurrence:** recordedBy: leg. C. Baseotto; individualCount: 1; lifeStage: adult; occurrenceID: 735E2067-E79F-5B56-AFB5-4A74C19A0696; **Taxon:** scientificName: Platyceruscaraboides (Linnaeus, 1758); order: Coleoptera; family: Lucanidae; **Location:** country: Italy; countryCode: IT; stateProvince: Udine; county: Socchieve; locality: Tramonti di sopra, Forca Monte Rest; verbatimElevation: 1096; **Event:** year: 2002; month: 6; day: 2; **Record Level:** collectionID: Coll. G. Stefani
- **Type status:** Other material. **Occurrence:** recordedBy: leg. G. Stefani; individualCount: 1; lifeStage: adult; occurrenceID: 5305C89E-23BF-5A7C-A430-96DE5FBB137E; **Taxon:** scientificName: Platyceruscaraboides (Linnaeus, 1758); order: Coleoptera; family: Lucanidae; **Location:** country: Italy; countryCode: IT; stateProvince: Udine; county: Taipana; locality: Montemaggiore; verbatimElevation: 935; **Event:** year: 2002; month: 6; day: 4; **Record Level:** collectionID: Coll. G. Stefani
- **Type status:** Other material. **Occurrence:** recordedBy: leg. E. Busolini; individualCount: 2; sex: male; lifeStage: adult; occurrenceID: 2A198536-4421-5C72-9769-A576982446B0; **Taxon:** scientificName: Platyceruscaraboides (Linnaeus, 1758); order: Coleoptera; family: Lucanidae; **Location:** country: Italy; countryCode: IT; stateProvince: Udine; county: Tarcento; **Event:** year: 1948; month: 5; day: 10; **Record Level:** collectionID: Coll. Museo di Storia Naturale di Venezia
- **Type status:** Other material. **Occurrence:** recordedBy: leg. L. Deganutti; individualCount: 1; sex: male; lifeStage: adult; occurrenceID: ABB20B1F-A984-5844-A17A-FA55D8B5E542; **Taxon:** scientificName: Platyceruscaraboides (Linnaeus, 1758); order: Coleoptera; family: Lucanidae; **Location:** country: Italy; countryCode: IT; stateProvince: Udine; county: Tarvisio; locality: Coccau; verbatimElevation: 935; decimalLatitude: 46.53189; decimalLongitude: 13.62325; geodeticDatum: WGS84; **Event:** year: 2023; month: 5–6
- **Type status:** Other material. **Occurrence:** recordedBy: leg. E. Colonnelli; individualCount: 3; sex: 2 males, 1 female; lifeStage: adult; occurrenceID: 2DB8F1E0-C937-514F-8A27-C6D759069F9E; **Taxon:** scientificName: Platyceruscaraboides (Linnaeus, 1758); order: Coleoptera; family: Lucanidae; **Location:** country: Italy; countryCode: IT; stateProvince: Rieti; county: Amatrice; locality: cima Lepri, Cascate delle Barche; verbatimElevation: 1750; **Event:** year: 2005; month: 6; day: 18; **Record Level:** collectionID: Coll. L. Bartolozzi
- **Type status:** Other material. **Occurrence:** recordedBy: leg. F. Vitali; individualCount: 1; lifeStage: adult; occurrenceID: DD89C798-C50D-5C15-A346-DDECFBCAC9A6; **Taxon:** scientificName: Platyceruscaraboides (Linnaeus, 1758); order: Coleoptera; family: Lucanidae; **Location:** country: Italy; countryCode: IT; stateProvince: Genova; county: Casella; locality: Santuario di Ns. Signora dell'Acqua; **Event:** year: 2004; month: 4; day: 16; **Record Level:** collectionID: Coll. F. Vitali
- **Type status:** Other material. **Occurrence:** individualCount: 1; sex: male; lifeStage: adult; occurrenceID: 863E7A01-12C4-5877-B5FA-D9B924309D6D; **Taxon:** scientificName: Platyceruscaraboides (Linnaeus, 1758); order: Coleoptera; family: Lucanidae; **Location:** country: Italy; countryCode: IT; stateProvince: Genova; county: Genova; locality: Colle di Creto; **Event:** year: 1900; month: 5; **Record Level:** collectionID: Coll. Museo Civico di Storia Naturale di Milano
- **Type status:** Other material. **Occurrence:** recordedBy: leg. D. Scaccini & M. Bonelli; individualCount: 1; lifeStage: adult; occurrenceID: 6869E0D5-4A93-5312-BEA8-697DBB525ABE; **Taxon:** scientificName: Platyceruscaraboides (Linnaeus, 1758); order: Coleoptera; family: Lucanidae; **Location:** country: Italy; countryCode: IT; stateProvince: Imperia; county: Alto; locality: close to Alto; decimalLatitude: 44.108636; decimalLongitude: 7.97595; geodeticDatum: WGS84; **Event:** year: 2019; month: 1; day: 4; **Record Level:** collectionID: Coll. D. Scaccini
- **Type status:** Other material. **Occurrence:** recordedBy: leg. Bastianini & Nappini; individualCount: 1; lifeStage: adult; occurrenceID: 2E77B14A-D782-5936-9A3F-3802806216A4; **Taxon:** scientificName: Platyceruscaraboides (Linnaeus, 1758); order: Coleoptera; family: Lucanidae; **Location:** country: Italy; countryCode: IT; stateProvince: Savona; county: Bardineto; verbatimElevation: 681; **Event:** year: 2015; month: 4; day: 1–5
- **Type status:** Other material. **Occurrence:** recordedBy: leg. D. Scaccini & M. Bonelli; individualCount: 1; lifeStage: adult; occurrenceID: 729BEE42-BC90-59BB-A0AB-EECFB68B2BDA; **Taxon:** scientificName: Platyceruscaraboides (Linnaeus, 1758); order: Coleoptera; family: Lucanidae; **Location:** country: Italy; countryCode: IT; stateProvince: Savona; county: Bardineto; decimalLatitude: 44.193583; decimalLongitude: 8.117806; geodeticDatum: WGS84; **Event:** year: 2019; month: 1; day: 3; **Record Level:** collectionID: Coll. D. Scaccini
- **Type status:** Other material. **Occurrence:** recordedBy: leg. F. Vitali; individualCount: 1; lifeStage: adult; occurrenceID: B99FA7C4-AA89-57ED-AAF4-681238CA2F84; **Taxon:** scientificName: Platyceruscaraboides (Linnaeus, 1758); order: Coleoptera; family: Lucanidae; **Location:** country: Italy; countryCode: IT; stateProvince: Savona; county: Colle del Giovo; locality: bosco di castagni; **Event:** year: 1989; month: 5; day: 7; **Record Level:** collectionID: Coll. F. Vitali
- **Type status:** Other material. **Occurrence:** recordedBy: leg. D. Pedersoli; individualCount: 1; lifeStage: adult; occurrenceID: 890DB431-5A30-5CB9-9327-39D92079FEB7; **Taxon:** scientificName: Platyceruscaraboides (Linnaeus, 1758); order: Coleoptera; family: Lucanidae; **Location:** country: Italy; countryCode: IT; stateProvince: Bergamo; county: Albino; locality: peak of Monte Misma; verbatimElevation: 1140; decimalLatitude: 45.736299; decimalLongitude: 9.818304; geodeticDatum: WGS84; **Event:** year: 2015; month: 5; day: 9
- **Type status:** Other material. **Occurrence:** recordedBy: leg. D. Scaccini; individualCount: 1; lifeStage: larva; occurrenceID: A43BCD70-A87F-5896-9A29-9683CAF5C1D2; **Taxon:** scientificName: Platyceruscaraboides (Linnaeus, 1758); order: Coleoptera; family: Lucanidae; **Location:** country: Italy; countryCode: IT; stateProvince: Bergamo; county: Averara; verbatimElevation: 781; decimalLatitude: 45.988733; decimalLongitude: 9.637397; geodeticDatum: WGS84; **Event:** year: 2015; month: 4; day: 7; **Record Level:** collectionID: Coll. D. Scaccini
- **Type status:** Other material. **Occurrence:** recordedBy: leg. D. Scaccini; individualCount: 1; occurrenceID: A74CB2ED-01C6-5696-B3F1-FC24178A7611; **Taxon:** scientificName: Platyceruscaraboides (Linnaeus, 1758); order: Coleoptera; family: Lucanidae; **Location:** country: Italy; countryCode: IT; stateProvince: Bergamo; county: Camerata Cornello; locality: trail from Lenna to San Giovanni Bianco; verbatimElevation: 473; decimalLatitude: 45.905422; decimalLongitude: 9.659919; geodeticDatum: WGS84; **Identification:** identificationRemarks: oviposition scars; **Event:** year: 2022; month: 12; day: 28; **Record Level:** collectionID: field observations by D. Scaccini
- **Type status:** Other material. **Occurrence:** recordedBy: leg. F. Tomasi; individualCount: 3; lifeStage: larva; occurrenceID: AAC634D0-F222-5922-AEAC-A4FF1C084D93; **Taxon:** scientificName: Platyceruscaraboides (Linnaeus, 1758); order: Coleoptera; family: Lucanidae; **Location:** country: Italy; countryCode: IT; stateProvince: Bergamo; county: Cassiglio; locality: from Cassiglio, trail to baita Foyer; verbatimElevation: 900; decimalLatitude: 45.94734; decimalLongitude: 9.617527; geodeticDatum: WGS84; **Event:** year: 2018
- **Type status:** Other material. **Occurrence:** recordedBy: leg. D. Pedersoli; individualCount: 1; lifeStage: adult; occurrenceID: 45EA5718-AC9E-59AD-B480-9639656F5C73; **Taxon:** scientificName: Platyceruscaraboides (Linnaeus, 1758); order: Coleoptera; family: Lucanidae; **Location:** country: Italy; countryCode: IT; stateProvince: Bergamo; county: Cene; locality: località Fontanei; verbatimElevation: 500; decimalLatitude: 45.777447; decimalLongitude: 9.832614; geodeticDatum: WGS84; **Event:** year: 2013; month: 5; day: 1; **Record Level:** collectionID: Coll. D. Pedersoli
- **Type status:** Other material. **Occurrence:** recordedBy: leg. D. Scaccini; individualCount: 4; lifeStage: larva; occurrenceID: 9F231426-AE92-5C10-9CEF-59394734B869; **Taxon:** scientificName: Platyceruscaraboides (Linnaeus, 1758); order: Coleoptera; family: Lucanidae; **Location:** country: Italy; countryCode: IT; stateProvince: Bergamo; county: Dossena; locality: close to "miniere di Dossena"; verbatimElevation: 1060; decimalLatitude: 45.897961; decimalLongitude: 9.682781; geodeticDatum: WGS84; **Event:** year: 2017; month: 8; day: 13; **Record Level:** collectionID: Coll. D. Scaccini
- **Type status:** Other material. **Occurrence:** recordedBy: leg. D. Pedersoli; individualCount: 2; lifeStage: adult; occurrenceID: 2D43E1C0-9FF1-565C-9A0B-F04B8F3D9DF2; **Taxon:** scientificName: Platyceruscaraboides (Linnaeus, 1758); order: Coleoptera; family: Lucanidae; **Location:** country: Italy; countryCode: IT; stateProvince: Bergamo; county: Fonteno; locality: close to Monte Boario; verbatimElevation: 1200; decimalLatitude: 45.762524; decimalLongitude: 10.006687; geodeticDatum: WGS84; **Event:** year: 2019; month: 5; day: 25; **Record Level:** collectionID: Coll. D. Pedersoli
- **Type status:** Other material. **Occurrence:** recordedBy: leg. D. Scaccini; individualCount: 1; lifeStage: larva; occurrenceID: F9B841F7-B53B-5DE7-AF38-96511CFC983A; **Taxon:** scientificName: Platyceruscaraboides (Linnaeus, 1758); order: Coleoptera; family: Lucanidae; **Location:** country: Italy; countryCode: IT; stateProvince: Bergamo; county: Lenna; verbatimElevation: 632; decimalLatitude: 45.929667; decimalLongitude: 9.673164; geodeticDatum: WGS84; **Event:** year: 2016; month: 12; day: 27; **Record Level:** collectionID: Coll. D. Scaccini
- **Type status:** Other material. **Occurrence:** recordedBy: leg. D. Scaccini; individualCount: 1; occurrenceID: 31632A43-B366-5F21-82F4-DF4CBF0D95F7; **Taxon:** scientificName: Platyceruscaraboides (Linnaeus, 1758); order: Coleoptera; family: Lucanidae; **Location:** country: Italy; countryCode: IT; stateProvince: Bergamo; county: Lenna; verbatimElevation: 477; decimalLatitude: 45.929592; decimalLongitude: 9.669292; geodeticDatum: WGS84; **Identification:** identificationRemarks: oviposition scars; **Event:** year: 2017; month: 12; day: 29; **Record Level:** collectionID: field observations by D. Scaccini
- **Type status:** Other material. **Occurrence:** recordedBy: leg. D. Scaccini; individualCount: 1; occurrenceID: 1864DF06-898F-5187-99F6-27A5A0BE029E; **Taxon:** scientificName: Platyceruscaraboides (Linnaeus, 1758); order: Coleoptera; family: Lucanidae; **Location:** country: Italy; countryCode: IT; stateProvince: Bergamo; county: Olmo al Brembo; locality: trail from Olmo al Brembo to Averara; verbatimElevation: 590; decimalLatitude: 45.974275; decimalLongitude: 9.644183; geodeticDatum: WGS84; **Identification:** identificationRemarks: oviposition scars; **Event:** year: 2022; month: 12; day: 27; **Record Level:** collectionID: field observations by D. Scaccini
- **Type status:** Other material. **Occurrence:** recordedBy: leg. D. Scaccini; individualCount: 1; lifeStage: larva; occurrenceID: F4456747-1B4B-566E-A4CD-92F8803932FB; **Taxon:** scientificName: Platyceruscaraboides (Linnaeus, 1758); order: Coleoptera; family: Lucanidae; **Location:** country: Italy; countryCode: IT; stateProvince: Bergamo; county: Olmo al Brembo-Piazza Brembana; locality: trail (high) from Olmo al Brembo to Piazza Brembana; verbatimElevation: 876; decimalLatitude: 45.961089; decimalLongitude: 9.67081; geodeticDatum: WGS84; **Event:** year: 2022; month: 1; day: 2; **Record Level:** collectionID: Coll. D. Scaccini
- **Type status:** Other material. **Occurrence:** recordedBy: leg. D. Scaccini; individualCount: 1; occurrenceID: F1B2A114-52B5-5B21-A719-348B086160D4; **Taxon:** scientificName: Platyceruscaraboides (Linnaeus, 1758); order: Coleoptera; family: Lucanidae; **Location:** country: Italy; countryCode: IT; stateProvince: Bergamo; county: Piazza Brembana; locality: close to the trail from Piazza Brembana to Olmo; verbatimElevation: 603; decimalLatitude: 45.95338; decimalLongitude: 9.663204; geodeticDatum: WGS84; **Identification:** identificationRemarks: oviposition scars; **Event:** year: 2023; month: 1; day: 2; **Record Level:** collectionID: field observations by D. Scaccini
- **Type status:** Other material. **Occurrence:** recordedBy: leg. D. Scaccini; individualCount: 1; occurrenceID: 585CB263-7252-5AD3-A18F-0AECC383E7B1; **Taxon:** scientificName: Platyceruscaraboides (Linnaeus, 1758); order: Coleoptera; family: Lucanidae; **Location:** country: Italy; countryCode: IT; stateProvince: Bergamo; county: Piazza Brembana; locality: on the gallery opening to Piazza Brembana; verbatimElevation: 548; decimalLatitude: 45.948826; decimalLongitude: 9.665377; geodeticDatum: WGS84; **Identification:** identificationRemarks: oviposition scars; **Event:** year: 2023; month: 1; day: 2; **Record Level:** collectionID: field observations by D. Scaccini
- **Type status:** Other material. **Occurrence:** recordedBy: leg. D. Scaccini; individualCount: 1; lifeStage: larva; occurrenceID: 7A5F9922-AE33-55D8-8394-5FAD23BF5343; **Taxon:** scientificName: Platyceruscaraboides (Linnaeus, 1758); order: Coleoptera; family: Lucanidae; **Location:** country: Italy; countryCode: IT; stateProvince: Bergamo; county: Piazzatorre; locality: Località Forcella; verbatimElevation: 1003; decimalLatitude: 45.982258; decimalLongitude: 9.6808; geodeticDatum: WGS84; **Event:** year: 2016; month: 8; day: 19; **Record Level:** collectionID: Coll. D. Scaccini
- **Type status:** Other material. **Occurrence:** recordedBy: leg. D. Scaccini; individualCount: 1; lifeStage: larva; occurrenceID: 1B9C108B-B849-5921-91A1-D84D715B0F67; **Taxon:** scientificName: Platyceruscaraboides (Linnaeus, 1758); order: Coleoptera; family: Lucanidae; **Location:** country: Italy; countryCode: IT; stateProvince: Bergamo; county: Piazzolo; locality: -; verbatimElevation: 795; decimalLatitude: 45.977786; decimalLongitude: 9.674511; geodeticDatum: WGS84; **Event:** year: 2015; month: 1; day: 2; **Record Level:** collectionID: Coll. D. Scaccini
- **Type status:** Other material. **Occurrence:** recordedBy: leg. D. Scaccini; individualCount: 1; lifeStage: larva; occurrenceID: 89D6006F-C4BC-54ED-A6F3-E8EEA275A060; **Taxon:** scientificName: Platyceruscaraboides (Linnaeus, 1758); order: Coleoptera; family: Lucanidae; **Location:** country: Italy; countryCode: IT; stateProvince: Bergamo; county: Piazzolo; locality: close to Santuario S. Rita; verbatimElevation: 760; decimalLatitude: 45.976078; decimalLongitude: 9.665964; geodeticDatum: WGS84; **Event:** year: 2022; month: 4; day: 28; **Record Level:** collectionID: Coll. D. Scaccini
- **Type status:** Other material. **Occurrence:** recordedBy: leg. D. Scaccini; individualCount: 16; lifeStage: larva; occurrenceID: FE9E1895-FF85-56D6-9896-0063CC75823A; **Taxon:** scientificName: Platyceruscaraboides (Linnaeus, 1758); order: Coleoptera; family: Lucanidae; **Location:** country: Italy; countryCode: IT; stateProvince: Bergamo; county: Piazzolo; locality: close to the waterfall; verbatimElevation: 897; decimalLatitude: 45.972932; decimalLongitude: 9.67605; geodeticDatum: WGS84; **Event:** year: 2019; month: 6; day: 15; **Record Level:** collectionID: Coll. D. Scaccini
- **Type status:** Other material. **Occurrence:** recordedBy: leg. D. Scaccini; individualCount: 1; lifeStage: larva; occurrenceID: D03F4DDD-00A3-5A2D-BECB-EEE140CB7902; **Taxon:** scientificName: Platyceruscaraboides (Linnaeus, 1758); order: Coleoptera; family: Lucanidae; **Location:** country: Italy; countryCode: IT; stateProvince: Bergamo; county: Piazzolo; locality: forest, close to a creek; verbatimElevation: 749; decimalLatitude: 45.977269; decimalLongitude: 9.672689; geodeticDatum: WGS84; **Event:** year: 2016; month: 8; day: 19; **Record Level:** collectionID: Coll. D. Scaccini
- **Type status:** Other material. **Occurrence:** recordedBy: leg. D. Scaccini; individualCount: 1; lifeStage: larva; occurrenceID: D963E4A0-6CDF-5E91-8C4A-709B66AE33B2; **Taxon:** scientificName: Platyceruscaraboides (Linnaeus, 1758); order: Coleoptera; family: Lucanidae; **Location:** country: Italy; countryCode: IT; stateProvince: Bergamo; county: Piazzolo; locality: forest, close to houses; verbatimElevation: 750; decimalLatitude: 45.977525; decimalLongitude: 9.672742; geodeticDatum: WGS84; **Event:** year: 2017; month: 4; day: 16; **Record Level:** collectionID: Coll. D. Scaccini
- **Type status:** Other material. **Occurrence:** recordedBy: leg. D. Scaccini; individualCount: 1; sex: male; lifeStage: adult; occurrenceID: 475B87BF-EF13-53F1-8AE3-55B8DEE6C6ED; **Taxon:** scientificName: Platyceruscaraboides (Linnaeus, 1758); order: Coleoptera; family: Lucanidae; **Location:** country: Italy; countryCode: IT; stateProvince: Bergamo; county: Piazzolo; locality: Pra' di Strac; verbatimElevation: 843; decimalLatitude: 45.974639; decimalLongitude: 9.671742; geodeticDatum: WGS84; **Event:** year: 2022; month: 6; day: 4; **Record Level:** collectionID: Coll. D. Scaccini
- **Type status:** Other material. **Occurrence:** recordedBy: leg. D. Scaccini; individualCount: 1; sex: male; lifeStage: adult; occurrenceID: 66AA6F9E-07D8-5AA4-AAC2-9AE8C3B48554; **Taxon:** scientificName: Platyceruscaraboides (Linnaeus, 1758); order: Coleoptera; family: Lucanidae; **Location:** country: Italy; countryCode: IT; stateProvince: Bergamo; county: Piazzolo; locality: via Grumello; verbatimElevation: 753; decimalLatitude: 45.97811; decimalLongitude: 9.672394; geodeticDatum: WGS84; **Event:** year: 2011; month: 4; day: 26; **Record Level:** collectionID: Coll. D. Scaccini
- **Type status:** Other material. **Occurrence:** recordedBy: leg. D. Pedersoli; individualCount: 1; lifeStage: adult; occurrenceID: C99FF837-60ED-59C5-80C2-C7552469986B; **Taxon:** scientificName: Platyceruscaraboides (Linnaeus, 1758); order: Coleoptera; family: Lucanidae; **Location:** country: Italy; countryCode: IT; stateProvince: Brescia; county: Cividate Camuno; locality: Località Margole; verbatimElevation: 300; decimalLatitude: 45.935525; decimalLongitude: 10.268804; geodeticDatum: WGS84; **Event:** year: 2020; month: 4; day: 28; **Record Level:** collectionID: Coll. D. Pedersoli
- **Type status:** Other material. **Occurrence:** recordedBy: leg. D. Pedersoli; individualCount: 1; lifeStage: adult; occurrenceID: B416C8BB-89D7-524D-B56B-A3109C94A32F; **Taxon:** scientificName: Platyceruscaraboides (Linnaeus, 1758); order: Coleoptera; family: Lucanidae; **Location:** country: Italy; countryCode: IT; stateProvince: Brescia; county: Corteno Golgi; locality: close to Santicolo; verbatimElevation: 880; decimalLatitude: 46.168719; decimalLongitude: 10.278764; geodeticDatum: WGS84; **Event:** year: 2016; month: 5; day: 8; **Record Level:** collectionID: Coll. D. Pedersoli
- **Type status:** Other material. **Occurrence:** recordedBy: leg. D. Pedersoli; individualCount: 1; sex: female; lifeStage: adult; occurrenceID: 3A43BF7D-7DB4-5334-9760-19070FC990A5; **Taxon:** scientificName: Platyceruscaraboides (Linnaeus, 1758); order: Coleoptera; family: Lucanidae; **Location:** country: Italy; countryCode: IT; stateProvince: Brescia; county: Corteno Golgi; locality: segheria di Corteno; verbatimElevation: 1000; decimalLatitude: 46.166263; decimalLongitude: 10.22429; geodeticDatum: WGS84; **Event:** year: 2015; month: 5; day: 24; **Record Level:** collectionID: Coll. D. Pedersoli
- **Type status:** Other material. **Occurrence:** recordedBy: leg. D. Pedersoli; individualCount: 1; lifeStage: adult; occurrenceID: 75554726-6FC0-548B-8B81-D6E8BBF08FFD; **Taxon:** scientificName: Platyceruscaraboides (Linnaeus, 1758); order: Coleoptera; family: Lucanidae; **Location:** country: Italy; countryCode: IT; stateProvince: Brescia; county: Darfo Boario Terme; locality: close to Dosso della Forcella; verbatimElevation: 600; **Event:** year: 2009; month: 4; day: 25; **Record Level:** collectionID: Coll. D. Pedersoli
- **Type status:** Other material. **Occurrence:** recordedBy: leg. D. Pedersoli; individualCount: 1; lifeStage: adult; occurrenceID: 388B0784-681A-51A9-B724-AAF94258C394; **Taxon:** scientificName: Platyceruscaraboides (Linnaeus, 1758); order: Coleoptera; family: Lucanidae; **Location:** country: Italy; countryCode: IT; stateProvince: Brescia; county: Darfo Boario Terme; locality: close to Dosso della Forcella; verbatimElevation: 600; **Event:** year: 2010; month: 4; day: 22; **Record Level:** collectionID: Coll. D. Pedersoli
- **Type status:** Other material. **Occurrence:** recordedBy: leg. D. Pedersoli; individualCount: 1; sex: male; lifeStage: adult; occurrenceID: 3D87F41D-A7DE-51DF-9A28-290588369F02; **Taxon:** scientificName: Platyceruscaraboides (Linnaeus, 1758); order: Coleoptera; family: Lucanidae; **Location:** country: Italy; countryCode: IT; stateProvince: Brescia; county: Darfo Boario Terme; locality: Prat de Là di Angone; verbatimElevation: 675; decimalLatitude: 45.910729; decimalLongitude: 10.197758; geodeticDatum: WGS84; **Event:** year: 2015; month: 5; day: 5; **Record Level:** collectionID: Coll. D. Pedersoli
- **Type status:** Other material. **Occurrence:** recordedBy: leg. D. Pedersoli; individualCount: 1; lifeStage: adult; occurrenceID: 21B5D8B0-7C76-5715-AC97-71C3205EC921; **Taxon:** scientificName: Platyceruscaraboides (Linnaeus, 1758); order: Coleoptera; family: Lucanidae; **Location:** country: Italy; countryCode: IT; stateProvince: Brescia; county: Darfo Boario Terme; locality: trail Angone-San Valentino; verbatimElevation: 450; decimalLatitude: 45.906169; decimalLongitude: 10.190481; geodeticDatum: WGS84; **Event:** year: 2016; month: 5; day: 3; **Record Level:** collectionID: Coll. D. Pedersoli
- **Type status:** Other material. **Occurrence:** recordedBy: leg. D. Pedersoli; individualCount: 1; lifeStage: adult; occurrenceID: 542FDF42-1011-51F0-B98A-BE4597A33E1C; **Taxon:** scientificName: Platyceruscaraboides (Linnaeus, 1758); order: Coleoptera; family: Lucanidae; **Location:** country: Italy; countryCode: IT; stateProvince: Brescia; county: Darfo Boario Terme; locality: trail Erbanno-San Valentino; verbatimElevation: 550; decimalLatitude: 45.912001; decimalLongitude: 10.200603; geodeticDatum: WGS84; **Event:** year: 2010; month: 5; day: 6; **Record Level:** collectionID: Coll. D. Pedersoli
- **Type status:** Other material. **Occurrence:** recordedBy: leg. D. Scaccini; individualCount: 1; lifeStage: larva; occurrenceID: CDA1990B-1865-502B-90CB-D265C1DB011E; **Taxon:** scientificName: Platyceruscaraboides (Linnaeus, 1758); order: Coleoptera; family: Lucanidae; **Location:** country: Italy; countryCode: IT; stateProvince: Brescia; county: Droane; locality: close to the cave n. 499; verbatimElevation: 854; decimalLatitude: 45.753689; decimalLongitude: 10.626742; geodeticDatum: WGS84; **Event:** year: 2020; month: 1; day: 3; **Record Level:** collectionID: Coll. D. Scaccini
- **Type status:** Other material. **Occurrence:** recordedBy: leg. D. Pedersoli; individualCount: 1; lifeStage: adult; occurrenceID: 94E7B186-AEBC-509B-9FE0-0F2DE63D666F; **Taxon:** scientificName: Platyceruscaraboides (Linnaeus, 1758); order: Coleoptera; family: Lucanidae; **Location:** country: Italy; countryCode: IT; stateProvince: Brescia; county: Esine; locality: Monte Bardisone, facing S; verbatimElevation: 350; decimalLatitude: 45.898875; decimalLongitude: 10.228894; geodeticDatum: WGS84; **Event:** year: 2020; month: 4; day: 23; **Record Level:** collectionID: Coll. D. Pedersoli
- **Type status:** Other material. **Occurrence:** recordedBy: leg. D. Scaccini; individualCount: 1; lifeStage: larva; occurrenceID: 4A984C07-E0A0-5CE7-83B4-5B1F9DED5ADB; **Taxon:** scientificName: Platyceruscaraboides (Linnaeus, 1758); order: Coleoptera; family: Lucanidae; **Location:** country: Italy; countryCode: IT; stateProvince: Brescia; county: Gargnano; locality: trail to Droane; verbatimElevation: 520; decimalLatitude: 45.735878; decimalLongitude: 10.606853; geodeticDatum: WGS84; **Event:** year: 2020; month: 1; day: 3; **Record Level:** collectionID: field observations by D. Scaccini
- **Type status:** Other material. **Occurrence:** recordedBy: leg. D. Pedersoli; individualCount: 1; lifeStage: adult; occurrenceID: B50B17E8-3D9E-5184-A0B5-1E8F4A021717; **Taxon:** scientificName: Platyceruscaraboides (Linnaeus, 1758); order: Coleoptera; family: Lucanidae; **Location:** country: Italy; countryCode: IT; stateProvince: Brescia; county: Pisogne; locality: trail n. 212 to Corna Trentapassi; verbatimElevation: 1100; decimalLatitude: 45.772106; decimalLongitude: 10.088582; geodeticDatum: WGS84; **Event:** year: 2014; month: 5; day: 10; **Record Level:** collectionID: Coll. D. Pedersoli
- **Type status:** Other material. **Occurrence:** recordedBy: leg. D. Pedersoli; individualCount: 1; lifeStage: adult; occurrenceID: 1D005C4A-241D-514D-AA8E-3D48AC51AA4C; **Taxon:** scientificName: Platyceruscaraboides (Linnaeus, 1758); order: Coleoptera; family: Lucanidae; **Location:** country: Italy; countryCode: IT; stateProvince: Brescia; county: Sale Marasino; locality: Località Noase; verbatimElevation: 860; decimalLatitude: 45.713596; decimalLongitude: 10.142522; geodeticDatum: WGS84; **Event:** year: 2016; month: 5; day: 4; **Record Level:** collectionID: Coll. D. Pedersoli
- **Type status:** Other material. **Occurrence:** recordedBy: leg. D. Scaccini; individualCount: 1; lifeStage: adult; occurrenceID: DA86F38A-7C1A-5C87-89FB-B238A7C87D34; **Taxon:** scientificName: Platyceruscaraboides (Linnaeus, 1758); order: Coleoptera; family: Lucanidae; **Location:** country: Italy; countryCode: IT; stateProvince: Brescia; county: Valvestino; locality: Bollone, surroundings; verbatimElevation: 778; decimalLatitude: 45.729419; decimalLongitude: 10.613225; geodeticDatum: WGS84; **Event:** year: 2020; month: 1; day: 4; **Record Level:** collectionID: Coll. D. Scaccini
- **Type status:** Other material. **Occurrence:** recordedBy: leg. D. Scaccini; individualCount: 1; lifeStage: larva; occurrenceID: 05DB8667-FB5E-5C65-9EE8-70B3C429D867; **Taxon:** scientificName: Platyceruscaraboides (Linnaeus, 1758); order: Coleoptera; family: Lucanidae; **Location:** country: Italy; countryCode: IT; stateProvince: Brescia; county: Valvestino; locality: Bollone, surroundings; verbatimElevation: 618; decimalLatitude: 45.732572; decimalLongitude: 10.609; geodeticDatum: WGS84; **Event:** year: 2020; month: 1; day: 4; **Record Level:** collectionID: Coll. D. Scaccini
- **Type status:** Other material. **Occurrence:** recordedBy: leg. D. Scaccini; individualCount: 1; lifeStage: larva; occurrenceID: E1967E43-8468-594C-BD9D-8B354855805D; **Taxon:** scientificName: Platyceruscaraboides (Linnaeus, 1758); order: Coleoptera; family: Lucanidae; **Location:** country: Italy; countryCode: IT; stateProvince: Brescia; county: Valvestino; locality: Bollone, surroundings; verbatimElevation: 930; decimalLatitude: 45.739322; decimalLongitude: 10.577094; geodeticDatum: WGS84; **Event:** year: 2020; month: 1; day: 4; **Record Level:** collectionID: field observations by D. Scaccini
- **Type status:** Other material. **Occurrence:** recordedBy: leg. D. Scaccini; individualCount: 7; lifeStage: larva; occurrenceID: 76C620B1-B7D2-5257-83FC-22185036CE55; **Taxon:** scientificName: Platyceruscaraboides (Linnaeus, 1758); order: Coleoptera; family: Lucanidae; **Location:** country: Italy; countryCode: IT; stateProvince: Brescia; county: Valvestino; locality: Bollone, surroundings; verbatimElevation: 908; decimalLatitude: 45.738175; decimalLongitude: 10.576083; geodeticDatum: WGS84; **Event:** year: 2020; month: 1; day: 4; **Record Level:** collectionID: Coll. D. Scaccini
- **Type status:** Other material. **Occurrence:** recordedBy: leg. D. Scaccini; individualCount: 1; lifeStage: larva; occurrenceID: F2438992-2662-58FE-896A-F3DB80225013; **Taxon:** scientificName: Platyceruscaraboides (Linnaeus, 1758); order: Coleoptera; family: Lucanidae; **Location:** country: Italy; countryCode: IT; stateProvince: Brescia; county: Valvestino; locality: Bollone, surroundings; verbatimElevation: 898; decimalLatitude: 45.740078; decimalLongitude: 10.578611; geodeticDatum: WGS84; **Event:** year: 2020; month: 1; day: 4; **Record Level:** collectionID: field observations by D. Scaccini
- **Type status:** Other material. **Occurrence:** individualCount: 2; sex: male; lifeStage: adult; occurrenceID: 8D1EFABC-F9FB-5815-806E-9660A917A0C7; **Taxon:** scientificName: Platyceruscaraboides (Linnaeus, 1758); order: Coleoptera; family: Lucanidae; **Location:** country: Italy; countryCode: IT; stateProvince: Como; county: Brunate; **Event:** year: 1935; month: 5; day: 21; **Record Level:** collectionID: Coll. Museo Civico di Storia Naturale di Milano
- **Type status:** Other material. **Occurrence:** recordedBy: leg. D. Scaccini; individualCount: 1; sex: male; lifeStage: adult; occurrenceID: 529E99AD-1ABA-5494-A79C-89197B0045E6; **Taxon:** scientificName: Platyceruscaraboides (Linnaeus, 1758); order: Coleoptera; family: Lucanidae; **Location:** country: Italy; countryCode: IT; stateProvince: Lecco; county: Galbiate; locality: close to Parco Archeologico dei Piani di Barra; verbatimElevation: 600; decimalLatitude: 45.833239; decimalLongitude: 9.3661; geodeticDatum: WGS84; **Event:** year: 2023; month: 4; day: 21; **Record Level:** collectionID: Coll. D. Scaccini
- **Type status:** Other material. **Occurrence:** individualCount: 1; occurrenceID: B3332A17-2FD9-5097-A97F-B5097A930002; **Taxon:** scientificName: Platyceruscaraboides (Linnaeus, 1758); order: Coleoptera; family: Lucanidae; **Location:** country: Italy; countryCode: IT; stateProvince: Lecco; county: Galbiate; verbatimElevation: 644; decimalLatitude: 45.834121; decimalLongitude: 9.375022; geodeticDatum: WGS84; **Identification:** identificationRemarks: oviposition scars; **Event:** year: 2023; month: 4; day: 21; **Record Level:** collectionID: field observations by D. Scaccini
- **Type status:** Other material. **Occurrence:** recordedBy: leg. D. Scaccini & M. Bonelli; individualCount: 1; lifeStage: larva; occurrenceID: 59508E46-4B2C-5E91-8DEB-A0FBF3A42EC9; **Taxon:** scientificName: Platyceruscaraboides (Linnaeus, 1758); order: Coleoptera; family: Lucanidae; **Location:** country: Italy; countryCode: IT; stateProvince: Lecco; county: Galbiate; verbatimElevation: 397; decimalLatitude: 45.802117; decimalLongitude: 9.368822; geodeticDatum: WGS84; **Event:** year: 2021; month: 4; day: 24
- **Type status:** Other material. **Occurrence:** recordedBy: leg. A. Focarile; individualCount: 1; sex: male; lifeStage: adult; occurrenceID: EA7C4297-6B86-5E02-AB0A-4FDAA5919CBC; **Taxon:** scientificName: Platyceruscaraboides (Linnaeus, 1758); order: Coleoptera; family: Lucanidae; **Location:** country: Italy; countryCode: IT; stateProvince: Lecco; county: Valmadrera; locality: Corni di Canzo; **Event:** year: 1946; month: 4; day: 20; **Record Level:** collectionID: Coll. Museo Civico di Storia Naturale di Milano
- **Type status:** Other material. **Occurrence:** recordedBy: leg. G. Giovagnoli; individualCount: 2; sex: male; lifeStage: adult; occurrenceID: BA495C6E-5777-5FD5-A666-48FEDD0B0ABB; **Taxon:** scientificName: Platyceruscaraboides (Linnaeus, 1758); order: Coleoptera; family: Lucanidae; **Location:** country: Italy; countryCode: IT; stateProvince: Ancona; county: Fabriano; locality: Valdicastro; **Event:** year: 2019; month: 5; day: 11; **Record Level:** collectionID: Coll. G. Giovagnoli
- **Type status:** Other material. **Occurrence:** recordedBy: leg. G. Giovagnoli; individualCount: 1; sex: male; lifeStage: adult; occurrenceID: 0824C467-573C-5643-94DF-F43A94B39AA5; **Taxon:** scientificName: Platyceruscaraboides (Linnaeus, 1758); order: Coleoptera; family: Lucanidae; **Location:** country: Italy; countryCode: IT; stateProvince: Fermo; county: Montefortino; locality: Capotenna; **Event:** year: 2019; month: 6; day: 15; **Record Level:** collectionID: Coll. G. Giovagnoli
- **Type status:** Other material. **Occurrence:** recordedBy: leg. G. Giovagnoli; individualCount: 2; sex: 1 male, 1 female; lifeStage: adult; occurrenceID: 5172C09C-E31F-5BD5-AE9B-14C0119CF92C; **Taxon:** scientificName: Platyceruscaraboides (Linnaeus, 1758); order: Coleoptera; family: Lucanidae; **Location:** country: Italy; countryCode: IT; stateProvince: Fermo; county: Montefortino; **Event:** year: 2011; month: 5; day: 28; **Record Level:** collectionID: Coll. F. Tomasi
- **Type status:** Other material. **Occurrence:** recordedBy: leg. G. Giovagnoli; individualCount: 2; sex: 1 male, 1 female; lifeStage: adult; occurrenceID: 56FCCA63-776D-5D57-BDF1-A3C31B63C96A; **Taxon:** scientificName: Platyceruscaraboides (Linnaeus, 1758); order: Coleoptera; family: Lucanidae; **Location:** country: Italy; countryCode: IT; stateProvince: Fermo; county: Montefortino; **Event:** year: 2011; month: 5; day: 28
- **Type status:** Other material. **Occurrence:** recordedBy: leg. G. Giovagnoli; individualCount: 1; sex: female; lifeStage: adult; occurrenceID: D839D0C7-F682-558F-B725-7E44A93E711E; **Taxon:** scientificName: Platyceruscaraboides (Linnaeus, 1758); order: Coleoptera; family: Lucanidae; **Location:** country: Italy; countryCode: IT; stateProvince: Fermo; county: Valleremita; **Event:** year: 2020; month: 5; day: 14; **Record Level:** collectionID: Coll. G. Giovagnoli
- **Type status:** Other material. **Occurrence:** recordedBy: leg. G. Giovagnoli; individualCount: 1; sex: male; lifeStage: adult; occurrenceID: 636D656F-7DC2-537C-AC8B-2F7B47E07308; **Taxon:** scientificName: Platyceruscaraboides (Linnaeus, 1758); order: Coleoptera; family: Lucanidae; **Location:** country: Italy; countryCode: IT; stateProvince: Macerata; county: Bolognola; locality: Fargno; **Event:** year: 2020; month: 6; day: 6; **Record Level:** collectionID: Coll. G. Giovagnoli
- **Type status:** Other material. **Occurrence:** recordedBy: leg. G. Giovagnoli; individualCount: 1; sex: female; lifeStage: adult; occurrenceID: BD942FE3-4E61-5D28-97E4-EA82E62C666A; **Taxon:** scientificName: Platyceruscaraboides (Linnaeus, 1758); order: Coleoptera; family: Lucanidae; **Location:** country: Italy; countryCode: IT; stateProvince: Macerata; county: Bolognola; locality: Pintura di Bolognola; **Event:** year: 2017; month: 6; day: 18; **Record Level:** collectionID: Coll. G. Giovagnoli
- **Type status:** Other material. **Occurrence:** recordedBy: leg. G. Giovagnoli; individualCount: 1; sex: male; lifeStage: adult; occurrenceID: F267D7A4-08A7-56EA-913A-280933158FF2; **Taxon:** scientificName: Platyceruscaraboides (Linnaeus, 1758); order: Coleoptera; family: Lucanidae; **Location:** country: Italy; countryCode: IT; stateProvince: Macerata; county: Esanatoglia; **Event:** year: 2015; month: 6; day: 13; **Record Level:** collectionID: Coll. G. Giovagnoli
- **Type status:** Other material. **Occurrence:** recordedBy: leg. G. Giovagnoli; individualCount: 2; sex: female; lifeStage: adult; occurrenceID: 87102AB2-FE56-55AB-B54E-FE2240C6D3F8; **Taxon:** scientificName: Platyceruscaraboides (Linnaeus, 1758); order: Coleoptera; family: Lucanidae; **Location:** country: Italy; countryCode: IT; stateProvince: Macerata; county: Sefro; locality: Val Scurosa; **Event:** year: 2019; month: 6; day: 1; **Record Level:** collectionID: Coll. G. Giovagnoli
- **Type status:** Other material. **Occurrence:** recordedBy: leg. Callegari; individualCount: 1; sex: female; lifeStage: adult; occurrenceID: 12BC163E-E207-52A3-9399-532740DB477E; **Taxon:** scientificName: Platyceruscaraboides (Linnaeus, 1758); order: Coleoptera; family: Lucanidae; **Location:** country: Italy; countryCode: IT; stateProvince: Pesaro-Urbino; county: -; locality: Monte Catria; verbatimElevation: 1000; **Event:** year: 1991; month: 6; day: 10; **Record Level:** collectionID: Coll. Museo di Storia Naturale di Venezia
- **Type status:** Other material. **Occurrence:** recordedBy: leg. Callegari; individualCount: 1; sex: male; lifeStage: adult; occurrenceID: 47A2E707-C4D2-5904-BF6E-03C41529A21D; **Taxon:** scientificName: Platyceruscaraboides (Linnaeus, 1758); order: Coleoptera; family: Lucanidae; **Location:** country: Italy; countryCode: IT; stateProvince: Pesaro-Urbino; county: -; locality: Monte Nerone; verbatimElevation: 1000; **Event:** year: 1991; month: 6; day: 1; **Record Level:** collectionID: Coll. Museo di Storia Naturale di Venezia
- **Type status:** Other material. **Occurrence:** recordedBy: leg. W. Pagliacci; individualCount: 3; sex: 2 males, 1 female; lifeStage: adult; occurrenceID: F5596813-C3CB-5433-8419-556B7D460F64; **Taxon:** scientificName: Platyceruscaraboides (Linnaeus, 1758); order: Coleoptera; family: Lucanidae; **Location:** country: Italy; countryCode: IT; stateProvince: Pesaro-Urbino; county: Cantiano; locality: Monte Catria; verbatimElevation: 1200; **Event:** year: 1988; month: 4; day: 17
- **Type status:** Other material. **Occurrence:** recordedBy: leg. L. Galbiati; individualCount: 1; sex: male; lifeStage: adult; occurrenceID: 57C6DDAA-84ED-599A-8703-20AF79AF42DE; **Taxon:** scientificName: Platyceruscaraboides (Linnaeus, 1758); order: Coleoptera; family: Lucanidae; **Location:** country: Italy; countryCode: IT; stateProvince: Alessandria; county: Caldirola; **Event:** year: 1983; month: 1; day: 29; **Record Level:** collectionID: Coll. Museo Civico di Storia Naturale di Milano
- **Type status:** Other material. **Occurrence:** recordedBy: leg. G. Curletti; individualCount: 1; lifeStage: adult; occurrenceID: 78963B4A-CC15-581A-A59D-511EC5C3F78C; **Taxon:** scientificName: Platyceruscaraboides (Linnaeus, 1758); order: Coleoptera; family: Lucanidae; **Location:** country: Italy; countryCode: IT; stateProvince: Biella; county: -; locality: Vallée Sessera; **Event:** year: 1995; month: 6; day: 25
- **Type status:** Other material. **Occurrence:** recordedBy: leg. G. Curletti; individualCount: 1; lifeStage: adult; occurrenceID: 8A79295D-9631-5BFD-ABDE-7A083312816E; **Taxon:** scientificName: Platyceruscaraboides (Linnaeus, 1758); order: Coleoptera; family: Lucanidae; **Location:** country: Italy; countryCode: IT; stateProvince: Biella; county: Casa del Bosco, Sostegno; **Event:** year: 1984; month: 6; day: 20
- **Type status:** Other material. **Occurrence:** recordedBy: leg. G. Curletti; individualCount: 1; lifeStage: adult; occurrenceID: CA3BAF67-57C2-58C3-B56B-A91875FAA44A; **Taxon:** scientificName: Platyceruscaraboides (Linnaeus, 1758); order: Coleoptera; family: Lucanidae; **Location:** country: Italy; countryCode: IT; stateProvince: Biella; county: Casa del Bosco, Sostegno; **Event:** year: 1994; month: 6; day: 13
- **Type status:** Other material. **Occurrence:** recordedBy: leg. M. Dutto; individualCount: 2; sex: 1 male, 1 female; lifeStage: adult; occurrenceID: D8D9664F-2D9A-5D93-8E38-14662D05060D; **Taxon:** scientificName: Platyceruscaraboides (Linnaeus, 1758); order: Coleoptera; family: Lucanidae; **Location:** country: Italy; countryCode: IT; stateProvince: Imola; county: -; locality: Breyl, F. Valle Roya; **Event:** year: 2002; month: 7; day: 15; **Record Level:** collectionID: Coll. Museo Civico di Storia Naturale di Carmagnola
- **Type status:** Other material. **Occurrence:** recordedBy: leg. Rochat; individualCount: 1; sex: female; lifeStage: adult; occurrenceID: 323E15A1-FF22-544C-929E-1125B1652386; **Taxon:** scientificName: Platyceruscaraboides (Linnaeus, 1758); order: Coleoptera; family: Lucanidae; **Location:** country: Italy; countryCode: IT; stateProvince: Turin; county: Prali; locality: Val Germanasca; **Event:** year: 1974; month: 7; **Record Level:** collectionID: Coll. Museo Civico di Storia Naturale di Milano
- **Type status:** Other material. **Occurrence:** recordedBy: leg. M. Zammataro; individualCount: 1; sex: female; lifeStage: adult; occurrenceID: F521CD06-1522-5394-A5EA-FA8DFAF5BC59; **Taxon:** scientificName: Platyceruscaraboides (Linnaeus, 1758); order: Coleoptera; family: Lucanidae; **Location:** country: Italy; countryCode: IT; stateProvince: Turin; county: Varisella; **Event:** year: 2018; month: 5; day: 9; **Record Level:** collectionID: Coll. G. Giovagnoli
- **Type status:** Other material. **Occurrence:** recordedBy: leg. Pescarolo; individualCount: 1; sex: male; lifeStage: adult; occurrenceID: 5075AB83-6510-551E-AD40-7D9C2D4BEA68; **Taxon:** scientificName: Platyceruscaraboides (Linnaeus, 1758); order: Coleoptera; family: Lucanidae; **Location:** country: Italy; countryCode: IT; stateProvince: Vercelli; county: -; locality: Casa del Bosco; **Event:** year: 1994; month: 4; day: 13; **Record Level:** collectionID: Coll. Museo Civico di Storia Naturale di Carmagnola
- **Type status:** Other material. **Occurrence:** recordedBy: leg. R. Colombini; individualCount: 1; sex: female; lifeStage: adult; occurrenceID: CF47367A-5E14-5745-A854-BF1C3D827F2A; **Taxon:** scientificName: Platyceruscaraboides (Linnaeus, 1758); order: Coleoptera; family: Lucanidae; **Location:** country: Italy; countryCode: IT; stateProvince: Vercelli; county: Carcoforo; locality: Val Sesia; verbatimElevation: 1200; **Event:** year: 1973; month: 6; day: 24; **Record Level:** collectionID: Coll. Museo Civico di Storia Naturale di Milano
- **Type status:** Other material. **Occurrence:** recordedBy: leg. Pescarolo; individualCount: 1; sex: male; lifeStage: adult; occurrenceID: A7040024-AD6E-515B-8B4A-3F237F5A4B7D; **Taxon:** scientificName: Platyceruscaraboides (Linnaeus, 1758); order: Coleoptera; family: Lucanidae; **Location:** country: Italy; countryCode: IT; stateProvince: Vercelli; county: Scopello-Mera; locality: Val Sesia; **Event:** year: 1984; month: 6; day: 20; **Record Level:** collectionID: Coll. Museo Civico di Storia Naturale di Carmagnola
- **Type status:** Other material. **Occurrence:** individualCount: 1; sex: male; lifeStage: adult; occurrenceID: 2CAC6174-749B-50FC-B617-E94F697944B1; **Taxon:** scientificName: Platyceruscaraboides (Linnaeus, 1758); order: Coleoptera; family: Lucanidae; **Location:** country: Italy; countryCode: IT; stateProvince: Bolzano; county: Tires; verbatimElevation: 1050; **Event:** year: 2010; month: 6; day: 11; **Record Level:** collectionID: Coll. M. Zilioli
- **Type status:** Other material. **Occurrence:** recordedBy: photo by D. Debiasi; individualCount: 1; sex: male; lifeStage: adult; occurrenceID: CE6C836C-D724-50FF-8259-27025BEAFE56; **Taxon:** scientificName: Platyceruscaraboides (Linnaeus, 1758); order: Coleoptera; family: Lucanidae; **Location:** country: Italy; countryCode: IT; stateProvince: Trento; county: -; locality: close to Altopiano della Vigolana; verbatimElevation: 868; **Event:** year: 2020; month: 4; day: 30
- **Type status:** Other material. **Occurrence:** individualCount: 1; lifeStage: adult; occurrenceID: 151987BF-8D91-5063-A398-D6448EF25B75; **Taxon:** scientificName: Platyceruscaraboides (Linnaeus, 1758); order: Coleoptera; family: Lucanidae; **Location:** country: Italy; countryCode: IT; stateProvince: Trento; county: -; locality: Passo del Ballino; verbatimElevation: 850; **Event:** year: 2001; month: 6; day: 2; **Record Level:** collectionID: Coll. A. Carlin
- **Type status:** Other material. **Occurrence:** individualCount: 1; lifeStage: adult; occurrenceID: DC998BE5-DBA2-535F-91C2-1A4024D6F93B; **Taxon:** scientificName: Platyceruscaraboides (Linnaeus, 1758); order: Coleoptera; family: Lucanidae; **Location:** country: Italy; countryCode: IT; stateProvince: Trento; county: Caldonazzo; locality: Torrente Centa, close to "pineta di Caldonazzo"; verbatimElevation: 440; **Event:** year: 2013; month: 4; day: 19; **Record Level:** collectionID: Coll. A. Carlin
- **Type status:** Other material. **Occurrence:** recordedBy: photo by D. Debiasi; individualCount: 1; sex: female; lifeStage: adult; occurrenceID: CCE6B8EC-2415-5631-8547-A99DF6BCFC9B; **Taxon:** scientificName: Platyceruscaraboides (Linnaeus, 1758); order: Coleoptera; family: Lucanidae; **Location:** country: Italy; countryCode: IT; stateProvince: Trento; county: Folgaria; verbatimElevation: 848; **Event:** year: 2020; month: 5; day: 5
- **Type status:** Other material. **Occurrence:** individualCount: 1; lifeStage: adult; occurrenceID: 4D731DBA-B1E4-52AE-B9AD-A8096A46FA0E; **Taxon:** scientificName: Platyceruscaraboides (Linnaeus, 1758); order: Coleoptera; family: Lucanidae; **Location:** country: Italy; countryCode: IT; stateProvince: Trento; county: Pergine Valsugana; locality: Colle del Tegazzo; verbatimElevation: 650; **Event:** year: 2014; month: 5; day: 6; **Record Level:** collectionID: Coll. A. Carlin
- **Type status:** Other material. **Occurrence:** recordedBy: leg. F. Marangon; individualCount: 1; sex: male; lifeStage: adult; occurrenceID: F44D2FEE-3D5C-5065-BC58-929209577CAF; **Taxon:** scientificName: Platyceruscaraboides (Linnaeus, 1758); order: Coleoptera; family: Lucanidae; **Location:** country: Italy; countryCode: IT; stateProvince: Trento; county: Rovereto; locality: Ca Bianca; verbatimElevation: 473; **Event:** year: 2017; month: 4; day: 30; **Record Level:** collectionID: Coll. D. Scaccini
- **Type status:** Other material. **Occurrence:** individualCount: 1; lifeStage: adult; occurrenceID: BE10C2CC-8135-5EFC-BA62-D509F0CBEF94; **Taxon:** scientificName: Platyceruscaraboides (Linnaeus, 1758); order: Coleoptera; family: Lucanidae; **Location:** country: Italy; countryCode: IT; stateProvince: Trento; county: Sega di Ala; verbatimElevation: 1280; **Event:** year: 1999; month: 4; day: 5; **Record Level:** collectionID: Coll. A. Carlin
- **Type status:** Other material. **Occurrence:** recordedBy: leg. N. Massimo; individualCount: 1; sex: male; lifeStage: adult; occurrenceID: 3748879D-8854-5E0E-91A9-D35837E442AD; **Taxon:** scientificName: Platyceruscaraboides (Linnaeus, 1758); order: Coleoptera; family: Lucanidae; **Location:** country: Italy; countryCode: IT; stateProvince: Trento; county: Trento; locality: Povo; verbatimElevation: 500; **Event:** year: 2019; month: 4; day: 30; **Record Level:** collectionID: Coll. F. Tomasi
- **Type status:** Other material. **Occurrence:** recordedBy: photo by D. Debiasi; individualCount: 1; sex: male; lifeStage: adult; occurrenceID: 4F1967E4-AC6D-50F0-9CCC-73E7F50CB637; **Taxon:** scientificName: Platyceruscaraboides (Linnaeus, 1758); order: Coleoptera; family: Lucanidae; **Location:** country: Italy; countryCode: IT; stateProvince: Trento; county: Vigolo Vattaro; verbatimElevation: 742; **Event:** year: 2020; month: 5; day: 2
- **Type status:** Other material. **Occurrence:** recordedBy: photo by D. Debiasi; individualCount: 2; sex: male; lifeStage: adult; occurrenceID: 19C7D9C1-8593-5AD2-BBAD-C4C96F50FA43; **Taxon:** scientificName: Platyceruscaraboides (Linnaeus, 1758); order: Coleoptera; family: Lucanidae; **Location:** country: Italy; countryCode: IT; stateProvince: Trento; county: Vigolo Vattaro; verbatimElevation: 1150; **Event:** year: 2020; month: 5; day: 4
- **Type status:** Other material. **Occurrence:** recordedBy: leg. B. Cecchi; individualCount: 1; sex: female; lifeStage: adult; occurrenceID: B5B6D84F-1F74-5ABF-8D38-02E7EAD3D563; **Taxon:** scientificName: Platyceruscaraboides (Linnaeus, 1758); order: Coleoptera; family: Lucanidae; **Location:** country: Italy; countryCode: IT; stateProvince: Arezzo; county: Poppi; locality: close to Camaldoli; verbatimElevation: 700; **Event:** year: 1993; month: 5; day: 31; **Record Level:** collectionID: Coll. L. Bartolozzi
- **Type status:** Other material. **Occurrence:** recordedBy: leg. M. Lombardi; individualCount: 1; sex: male; lifeStage: adult; occurrenceID: 3F7D69B3-9D67-5E87-BDAA-73028995B949; **Taxon:** scientificName: Platyceruscaraboides (Linnaeus, 1758); order: Coleoptera; family: Lucanidae; **Location:** country: Italy; countryCode: IT; stateProvince: Florence; county: Calenzano; locality: Monte Calvana; **Event:** year: 1928; **Record Level:** collectionID: Coll. Museo Civico di Storia Naturale di Milano
- **Type status:** Other material. **Occurrence:** recordedBy: leg. L. Bartolozzi; individualCount: 1; sex: male; lifeStage: adult; occurrenceID: DC15BE3A-FA9A-5F20-B550-9EE625D197C1; **Taxon:** scientificName: Platyceruscaraboides (Linnaeus, 1758); order: Coleoptera; family: Lucanidae; **Location:** country: Italy; countryCode: IT; stateProvince: Florence; county: Figline e Incisa Valdarno; locality: close to località Cinipetta (43°34'12"N 11°25'14"E), frazione di Gaville; verbatimElevation: 445; decimalLatitude: 43.57; decimalLongitude: 11.420556; geodeticDatum: WGS84; **Event:** year: 2021; month: 5; day: 6; **Record Level:** collectionID: Coll. L. Bartolozzi
- **Type status:** Other material. **Occurrence:** recordedBy: leg. M. Lombardi; individualCount: 1; sex: female; lifeStage: adult; occurrenceID: D2334963-5254-5D8D-BD05-41F01C873C2E; **Taxon:** scientificName: Platyceruscaraboides (Linnaeus, 1758); order: Coleoptera; family: Lucanidae; **Location:** country: Italy; countryCode: IT; stateProvince: Florence; county: Florence; locality: close to Florence; **Event:** year: 1915; **Record Level:** collectionID: Coll. Museo Civico di Storia Naturale di Milano
- **Type status:** Other material. **Occurrence:** recordedBy: leg. Callegari; individualCount: 1; sex: male; lifeStage: adult; occurrenceID: B9BE6E47-0159-5946-A17A-CA4FDC80052B; **Taxon:** scientificName: Platyceruscaraboides (Linnaeus, 1758); order: Coleoptera; family: Lucanidae; **Location:** country: Italy; countryCode: IT; stateProvince: Florence; county: Marradi; locality: Passo Peschiera; **Event:** year: 1984; month: 6; day: 1; **Record Level:** collectionID: Coll. Museo di Storia Naturale di Venezia
- **Type status:** Other material. **Occurrence:** recordedBy: leg. M. Faggi; individualCount: 1; lifeStage: adult; occurrenceID: 7A83B883-728D-5033-8695-B8A4638E5A97; **Taxon:** scientificName: Platyceruscaraboides (Linnaeus, 1758); order: Coleoptera; family: Lucanidae; **Location:** country: Italy; countryCode: IT; stateProvince: Pisa; county: Santa Luce; locality: Bosco di Santa Luce; **Event:** month: 6
- **Type status:** Other material. **Occurrence:** recordedBy: leg. F. Faggi; individualCount: 1; lifeStage: adult; occurrenceID: 06E40865-1603-5659-9B4A-85E503F7B2B8; **Taxon:** scientificName: Platyceruscaraboides (Linnaeus, 1758); order: Coleoptera; family: Lucanidae; **Location:** country: Italy; countryCode: IT; stateProvince: Prato; county: Cantagallo; locality: Acquiputoli; verbatimElevation: 1000; **Event:** year: 2005
- **Type status:** Other material. **Occurrence:** recordedBy: leg. F. Fabbriciani; individualCount: 3; sex: 2 males, 1 female; lifeStage: adult; occurrenceID: 13412ACF-F025-5131-9A05-505FB99AB663; **Taxon:** scientificName: Platyceruscaraboides (Linnaeus, 1758); order: Coleoptera; family: Lucanidae; **Location:** country: Italy; countryCode: IT; stateProvince: Prato; county: Cantagallo; locality: Cascina di Spedaletto; verbatimElevation: 928; **Event:** year: 2019; month: 5; day: 23
- **Type status:** Other material. **Occurrence:** recordedBy: leg. F. Fabbriciani; individualCount: 2; sex: male; lifeStage: adult; occurrenceID: 36FAC257-6F73-5EA0-AE0E-6C8D11C2A0C6; **Taxon:** scientificName: Platyceruscaraboides (Linnaeus, 1758); order: Coleoptera; family: Lucanidae; **Location:** country: Italy; countryCode: IT; stateProvince: Prato; county: Vaiano; locality: Monti di Calvana mt. 550, presso grotta Buca del Tasso; verbatimElevation: 501; **Event:** year: 2017; month: 5; day: 10
- **Type status:** Other material. **Occurrence:** recordedBy: leg. E. Colonnelli; individualCount: 1; sex: male; lifeStage: adult; occurrenceID: 697DF48F-8B73-5E0C-A76E-5A7805087F15; **Taxon:** scientificName: Platyceruscaraboides (Linnaeus, 1758); order: Coleoptera; family: Lucanidae; **Location:** country: Italy; countryCode: IT; stateProvince: Siena; county: Chiusdino; locality: Abbazia di San Galgano; verbatimElevation: 300; **Event:** year: 2013; month: 4; day: 25; **Record Level:** collectionID: Coll. L. Bartolozzi
- **Type status:** Other material. **Occurrence:** recordedBy: leg. D. Scaccini, E. Ruzzier, A. Serafin; individualCount: 1; lifeStage: larva; occurrenceID: ABDBA104-7F86-544B-B719-7DB99610B9A7; **Taxon:** scientificName: Platyceruscaraboides (Linnaeus, 1758); order: Coleoptera; family: Lucanidae; **Location:** country: Italy; countryCode: IT; stateProvince: Belluno; county: Alano di Piave; verbatimElevation: 786; decimalLatitude: 45.883957; decimalLongitude: 11.878955; geodeticDatum: WGS84; **Event:** year: 2023; month: 5; day: 6
- **Type status:** Other material. **Occurrence:** recordedBy: leg. F. Giannone; individualCount: 1; sex: male; lifeStage: adult; occurrenceID: 60D8AB2E-C9D1-5C9E-B456-5F8483229F51; **Taxon:** scientificName: Platyceruscaraboides (Linnaeus, 1758); order: Coleoptera; family: Lucanidae; **Location:** country: Italy; countryCode: IT; stateProvince: Belluno; county: Caiada, Faè; locality: Caiada, Faè; **Event:** year: 2000; month: 12; day: 8; **Record Level:** collectionID: Coll. D. Scaccini
- **Type status:** Other material. **Occurrence:** recordedBy: leg. E. Ruzzier; individualCount: 2; lifeStage: adult; occurrenceID: DDBFBF07-EE2C-522D-B5C9-148503CED1D9; **Taxon:** scientificName: Platyceruscaraboides (Linnaeus, 1758); order: Coleoptera; family: Lucanidae; **Location:** country: Italy; countryCode: IT; stateProvince: Belluno; county: Pedavena; locality: Località Norcen; verbatimElevation: 643; decimalLatitude: 46.052179; decimalLongitude: 11.867973; geodeticDatum: WGS84; **Event:** year: 2012; month: 4; day: 23
- **Type status:** Other material. **Occurrence:** recordedBy: leg. D. Scaccini, E. Ruzzier, A. Serafin; individualCount: 1; sex: male; lifeStage: adult; occurrenceID: EDA7B8A6-67BD-5C48-BDEB-0ED9DDA53CB5; **Taxon:** scientificName: Platyceruscaraboides (Linnaeus, 1758); order: Coleoptera; family: Lucanidae; **Location:** country: Italy; countryCode: IT; stateProvince: Belluno; county: Quero; verbatimElevation: 462; decimalLatitude: 45.946296; decimalLongitude: 11.876619; geodeticDatum: WGS84; **Event:** year: 2023; month: 5; day: 6; **Record Level:** collectionID: Coll. D. Scaccini
- **Type status:** Other material. **Occurrence:** individualCount: 1; lifeStage: adult; occurrenceID: CDDCBD54-58E0-5C00-945B-C2AB82EC9241; **Taxon:** scientificName: Platyceruscaraboides (Linnaeus, 1758); order: Coleoptera; family: Lucanidae; **Location:** country: Italy; countryCode: IT; stateProvince: Padova; county: Cinto Euganeo; locality: Euganean Hills; **Event:** year: 2017; month: 11; day: 1
- **Type status:** Other material. **Occurrence:** individualCount: 1; lifeStage: larva; occurrenceID: 6DBF2A50-B63E-5AE7-B281-21CE4AB8C19E; **Taxon:** scientificName: Platyceruscaraboides (Linnaeus, 1758); order: Coleoptera; family: Lucanidae; **Location:** country: Italy; countryCode: IT; stateProvince: Padua; county: Cinto Euganeo; locality: Monte Fasolo, Euganean Hills; verbatimElevation: 201; **Event:** year: 2017; month: 11; day: 1
- **Type status:** Other material. **Occurrence:** recordedBy: leg. D. Scaccini; individualCount: 1; lifeStage: larva; occurrenceID: 519999C7-0F1A-55EF-BA6B-CBBBA791D655; **Taxon:** scientificName: Platyceruscaraboides (Linnaeus, 1758); order: Coleoptera; family: Lucanidae; **Location:** country: Italy; countryCode: IT; stateProvince: Padua; county: Cinto Euganeo; locality: Monte Fasolo, Euganean Hills; verbatimElevation: 253; decimalLatitude: 45.292103; decimalLongitude: 11.695728; geodeticDatum: WGS84; **Event:** year: 2017; month: 11; day: 1; **Record Level:** collectionID: Coll. D. Scaccini
- **Type status:** Other material. **Occurrence:** recordedBy: leg. D. Scaccini; individualCount: 1; sex: female; lifeStage: adult; occurrenceID: EB73E519-36E3-52F9-BA33-BA7AAFE9B736; **Taxon:** scientificName: Platyceruscaraboides (Linnaeus, 1758); order: Coleoptera; family: Lucanidae; **Location:** country: Italy; countryCode: IT; stateProvince: Padua; county: Cinto Euganeo; locality: Monte Fasolo, Euganean Hills; verbatimElevation: 220; decimalLatitude: 45.293119; decimalLongitude: 11.694881; geodeticDatum: WGS84; **Event:** year: 2019; month: 3; day: 9; **Record Level:** collectionID: Coll. D. Scaccini
- **Type status:** Other material. **Occurrence:** recordedBy: leg. M. Volpones; individualCount: 1; lifeStage: adult; occurrenceID: 8E3091CA-9A3D-5EC4-9142-599C11C63D2E; **Taxon:** scientificName: Platyceruscaraboides (Linnaeus, 1758); order: Coleoptera; family: Lucanidae; **Location:** country: Italy; countryCode: IT; stateProvince: Treviso; county: Col Visentin; **Event:** year: 1987; month: 5; day: 3; **Record Level:** collectionID: Coll. D. Sechi
- **Type status:** Other material. **Occurrence:** recordedBy: leg. F. Fabbriciani; individualCount: 1; sex: male; lifeStage: adult; occurrenceID: ED22EBD1-FCDA-5241-9EDB-9B8CF6A3A823; **Taxon:** scientificName: Platyceruscaraboides (Linnaeus, 1758); order: Coleoptera; family: Lucanidae; **Location:** country: Italy; countryCode: IT; stateProvince: Treviso; county: Croda Rossa; verbatimElevation: 500; **Event:** year: 2009; month: 5; day: 30
- **Type status:** Other material. **Occurrence:** recordedBy: leg. D. Scaccini; individualCount: 4; lifeStage: larva; occurrenceID: 9583551C-F2BD-5777-BB00-2139D0650C6A; **Taxon:** scientificName: Platyceruscaraboides (Linnaeus, 1758); order: Coleoptera; family: Lucanidae; **Location:** country: Italy; countryCode: IT; stateProvince: Treviso; county: Maser; locality: close to "Sentieri Collinari"; verbatimElevation: 371; decimalLatitude: 45.821786; decimalLongitude: 11.961641; geodeticDatum: WGS84; **Event:** year: 2023; month: 4; day: 29; **Record Level:** collectionID: Coll. D. Scaccini
- **Type status:** Other material. **Occurrence:** recordedBy: leg. D. Scaccini; individualCount: 1; occurrenceID: 17BC18A4-B9EF-5B79-BCB0-4B6334D3F40D; **Taxon:** scientificName: Platyceruscaraboides (Linnaeus, 1758); order: Coleoptera; family: Lucanidae; **Location:** country: Italy; countryCode: IT; stateProvince: Treviso; county: Maser; locality: close to "Sentieri Collinari"; verbatimElevation: 357; decimalLatitude: 45.820318; decimalLongitude: 11.962482; geodeticDatum: WGS84; **Identification:** identificationRemarks: oviposition scars; **Event:** year: 2023; month: 4; day: 29
- **Type status:** Other material. **Occurrence:** recordedBy: leg. D. Scaccini; individualCount: 1; lifeStage: larva; occurrenceID: E006F251-82DA-5BF1-9744-61399506F7A4; **Taxon:** scientificName: Platyceruscaraboides (Linnaeus, 1758); order: Coleoptera; family: Lucanidae; **Location:** country: Italy; countryCode: IT; stateProvince: Treviso; county: Maser; locality: close to the mount peak; verbatimElevation: 454; decimalLatitude: 45.820359; decimalLongitude: 11.960273; geodeticDatum: WGS84; **Event:** year: 2023; month: 4; day: 29; **Record Level:** collectionID: Coll. D. Scaccini
- **Type status:** Other material. **Occurrence:** recordedBy: leg. D. Scaccini & E. Ruzzier; individualCount: 1; occurrenceID: 5BC831ED-87E7-5C11-8D45-DE0E23C03F06; **Taxon:** scientificName: Platyceruscaraboides (Linnaeus, 1758); order: Coleoptera; family: Lucanidae; **Location:** country: Italy; countryCode: IT; stateProvince: Treviso; county: Pieve del Grappa; locality: Crespano del Grappa; verbatimElevation: 600; decimalLatitude: 45.849641; decimalLongitude: 11.823344; geodeticDatum: WGS84; **Identification:** identificationRemarks: oviposition scars; **Event:** year: 2023; month: 4; day: 10; **Record Level:** collectionID: field observations by D. Scaccini
- **Type status:** Other material. **Occurrence:** recordedBy: leg. D. Scaccini & E. Ruzzier; individualCount: 2; lifeStage: larva; occurrenceID: CF017720-AFFE-534C-97BC-7C83F9AAA0FD; **Taxon:** scientificName: Platyceruscaraboides (Linnaeus, 1758); order: Coleoptera; family: Lucanidae; **Location:** country: Italy; countryCode: IT; stateProvince: Treviso; county: Pieve del Grappa; locality: Crespano del Grappa; verbatimElevation: 669; decimalLatitude: 45.851703; decimalLongitude: 11.820423; geodeticDatum: WGS84; **Event:** year: 2023; month: 4; day: 10
- **Type status:** Other material. **Occurrence:** individualCount: 1; lifeStage: adult; occurrenceID: 0F34E0E1-82A3-54BC-981D-D5C0A01D02C0; **Taxon:** scientificName: Platyceruscaraboides (Linnaeus, 1758); order: Coleoptera; family: Lucanidae; **Location:** country: Italy; countryCode: IT; stateProvince: Verona; county: Brentino Veronese; locality: trail to Santuario Madonna della Corona; verbatimElevation: 500; **Event:** year: 2017; month: 5; day: 11; **Record Level:** collectionID: Coll. A. Carlin
- **Type status:** Other material. **Occurrence:** recordedBy: leg. D. Scaccini; individualCount: 1; occurrenceID: D09FD756-C8E6-5733-AE26-6DA1B416E10C; **Taxon:** scientificName: Platyceruscaraboides (Linnaeus, 1758); order: Coleoptera; family: Lucanidae; **Location:** country: Italy; countryCode: IT; stateProvince: Verona; county: Ponte di Veja; verbatimElevation: 630; decimalLatitude: 45.607275; decimalLongitude: 10.971805; geodeticDatum: WGS84; **Identification:** identificationRemarks: oviposition scars; **Event:** year: 2022; month: 10; day: 22; **Record Level:** collectionID: field observations by D. Scaccini
- **Type status:** Other material. **Occurrence:** recordedBy: leg. F. Sanna; individualCount: 1; sex: male; lifeStage: adult; occurrenceID: 9048D24B-16BB-5499-B380-123F186CEE92; **Taxon:** scientificName: Platyceruscaraboides (Linnaeus, 1758); order: Coleoptera; family: Lucanidae; **Location:** country: Italy; countryCode: IT; stateProvince: Verona; county: Rivoli Veronese; locality: close to 37010 Rivoli Veronese VR; verbatimElevation: 239; decimalLatitude: 45.557275; decimalLongitude: 10.806746; geodeticDatum: WGS84; **Event:** year: 2020; month: 5; day: 5
- **Type status:** Other material. **Occurrence:** recordedBy: leg. Rallo; individualCount: 1; sex: male; lifeStage: adult; occurrenceID: 8B0F5004-6F39-5296-9AC5-66401452A33B; **Taxon:** scientificName: Platyceruscaraboides (Linnaeus, 1758); order: Coleoptera; family: Lucanidae; **Location:** country: Italy; countryCode: IT; stateProvince: Vicenza; county: Carpané; locality: Valsugana, river Brenta; **Event:** year: 1987; month: 4; day: 27; **Record Level:** collectionID: Coll. Museo di Storia Naturale di Venezia
- **Type status:** Other material. **Occurrence:** individualCount: 1; occurrenceID: D171648A-85EE-5615-A0AF-5F50771A9671; **Taxon:** scientificName: Platyceruscaraboides (Linnaeus, 1758); order: Coleoptera; family: Lucanidae; **Location:** country: Italy; countryCode: IT; stateProvince: Vicenza; county: Crespadoro; locality: close to Graizzari di Sopra; verbatimElevation: 621; decimalLatitude: 45.649004; decimalLongitude: 11.1881; geodeticDatum: WGS84; **Identification:** identificationRemarks: oviposition scars; **Event:** year: 2022; month: 1; day: 8; **Record Level:** collectionID: field observations by D. Scaccini
- **Type status:** Other material. **Occurrence:** individualCount: 1; occurrenceID: FFC85BF8-6B72-54D7-8F90-8F2686D554BA; **Taxon:** scientificName: Platyceruscaraboides (Linnaeus, 1758); order: Coleoptera; family: Lucanidae; **Location:** country: Italy; countryCode: IT; stateProvince: Vicenza; county: Crespadoro; locality: close to Graizzari di Sotto; verbatimElevation: 570; decimalLatitude: 45.648425; decimalLongitude: 11.192186; geodeticDatum: WGS84; **Identification:** identificationRemarks: oviposition scars; **Event:** year: 2022; month: 1; day: 8; **Record Level:** collectionID: field observations by D. Scaccini
- **Type status:** Other material. **Occurrence:** individualCount: 1; occurrenceID: A34B909F-58A9-5F25-857D-F44734BF4A28; **Taxon:** scientificName: Platyceruscaraboides (Linnaeus, 1758); order: Coleoptera; family: Lucanidae; **Location:** country: Italy; countryCode: IT; stateProvince: Vicenza; county: Crespadoro; locality: close to Zanconati; verbatimElevation: 689; decimalLatitude: 45.648667; decimalLongitude: 11.196249; geodeticDatum: WGS84; **Identification:** identificationRemarks: oviposition scars; **Event:** year: 2022; month: 1; day: 8; **Record Level:** collectionID: field observations by D. Scaccini
- **Type status:** Other material. **Occurrence:** individualCount: 1; sex: male; lifeStage: adult; occurrenceID: 5EE469B0-0941-5F0A-A282-2296155EA0A4; **Taxon:** scientificName: Platyceruscaraboides (Linnaeus, 1758); order: Coleoptera; family: Lucanidae; **Location:** country: Italy; countryCode: IT; stateProvince: Vicenza; county: Longare; locality: Lumignano; **Event:** year: 1950; month: 5; day: 5; **Record Level:** collectionID: Coll. Museo Civico di Storia Naturale di Milano
- **Type status:** Other material. **Occurrence:** recordedBy: leg. D. Scaccini; individualCount: 2; lifeStage: larva; occurrenceID: F3BDA51F-77A9-5F90-8255-2595CF02B084; **Taxon:** scientificName: Platyceruscaraboides (Linnaeus, 1758); order: Coleoptera; family: Lucanidae; **Location:** country: Italy; countryCode: IT; stateProvince: Vicenza; county: Malo; locality: close to Porra; verbatimElevation: 424; decimalLatitude: 45.623497; decimalLongitude: 11.389189; geodeticDatum: WGS84; **Event:** year: 2023; month: 4; day: 8
- **Type status:** Other material. **Occurrence:** recordedBy: leg. D. Scaccini & E. Ruzzier; individualCount: 6; lifeStage: larva; occurrenceID: DADEAA6C-9A8A-5067-A614-6C0292CE3F19; **Taxon:** scientificName: Platyceruscaraboides (Linnaeus, 1758); order: Coleoptera; family: Lucanidae; **Location:** country: Italy; countryCode: IT; stateProvince: Vicenza; county: Monte di Malo; locality: giro panoramico Montepian; verbatimElevation: 447; decimalLatitude: 45.63558; decimalLongitude: 11.39334; geodeticDatum: WGS84; **Event:** year: 2023; month: 4; day: 8
- **Type status:** Other material. **Occurrence:** recordedBy: leg. D. Scaccini & E. Ruzzier; individualCount: 2; lifeStage: larva; occurrenceID: 12F7FE00-F162-5B6B-B3A7-FD3877745BA6; **Taxon:** scientificName: Platyceruscaraboides (Linnaeus, 1758); order: Coleoptera; family: Lucanidae; **Location:** country: Italy; countryCode: IT; stateProvince: Vicenza; county: Monte di Malo; locality: giro panoramico Montepian; verbatimElevation: 430; decimalLatitude: 45.63658; decimalLongitude: 11.393414; geodeticDatum: WGS84; **Event:** year: 2023; month: 4; day: 8
- **Type status:** Other material. **Occurrence:** recordedBy: leg. D. Scaccini; individualCount: 2; lifeStage: larva; occurrenceID: 90EB1301-4C15-5546-AC02-A4E5DE4A8E68; **Taxon:** scientificName: Platyceruscaraboides (Linnaeus, 1758); order: Coleoptera; family: Lucanidae; **Location:** country: Italy; countryCode: IT; stateProvince: Vicenza; county: Monte di Malo; verbatimElevation: 595; decimalLatitude: 45.650388; decimalLongitude: 11.3527; geodeticDatum: WGS84; **Event:** year: 2023; month: 4; day: 8
- **Type status:** Other material. **Occurrence:** recordedBy: leg. D. Scaccini; individualCount: 1; lifeStage: larva; occurrenceID: D4FCA37E-6429-520E-94BE-4668D7906869; **Taxon:** scientificName: Platyceruscaraboides (Linnaeus, 1758); order: Coleoptera; family: Lucanidae; **Location:** country: Italy; countryCode: IT; stateProvince: Vicenza; county: Monte di Malo; verbatimElevation: 634; decimalLatitude: 45.649275; decimalLongitude: 11.351797; geodeticDatum: WGS84; **Event:** year: 2023; month: 4; day: 8
- **Type status:** Other material. **Occurrence:** recordedBy: leg. D. Scaccini; individualCount: 1; lifeStage: larva; occurrenceID: 6A2E35DA-3A8F-548A-8572-8D327BEB7EAA; **Taxon:** scientificName: Platyceruscaraboides (Linnaeus, 1758); order: Coleoptera; family: Lucanidae; **Location:** country: Italy; countryCode: IT; stateProvince: Vicenza; county: Schio; locality: close to Costenieri, place A; verbatimElevation: 715; decimalLatitude: 45.744805; decimalLongitude: 11.311242; geodeticDatum: WGS84; **Event:** year: 2023; month: 3; day: 25; **Record Level:** collectionID: Coll. D. Scaccini
- **Type status:** Other material. **Occurrence:** recordedBy: leg. D. Scaccini; individualCount: 1; lifeStage: larva; occurrenceID: 87DDB378-BFBF-507C-BB94-7BDD3B729A79; **Taxon:** scientificName: Platyceruscaraboides (Linnaeus, 1758); order: Coleoptera; family: Lucanidae; **Location:** country: Italy; countryCode: IT; stateProvince: Vicenza; county: Schio; locality: close to Costenieri, place B; verbatimElevation: 738; decimalLatitude: 45.745295; decimalLongitude: 11.310659; geodeticDatum: WGS84; **Event:** year: 2023; month: 3; day: 25; **Record Level:** collectionID: Coll. D. Scaccini
- **Type status:** Other material. **Occurrence:** recordedBy: leg. D. Scaccini; individualCount: 5; lifeStage: larva; occurrenceID: 311523EF-C1AA-56FD-B88D-54EAB3FEAB20; **Taxon:** scientificName: Platyceruscaraboides (Linnaeus, 1758); order: Coleoptera; family: Lucanidae; **Location:** country: Italy; countryCode: IT; stateProvince: Vicenza; county: Schio; locality: Sentiero di Fiaba; verbatimElevation: 936; decimalLatitude: 45.752857; decimalLongitude: 11.320698; geodeticDatum: WGS84; **Event:** year: 2023; month: 3; day: 25; **Record Level:** collectionID: Coll. D. Scaccini
- **Type status:** Other material. **Occurrence:** recordedBy: leg. D. Zanacco; individualCount: 1; lifeStage: adult; occurrenceID: BF4C75C7-5384-552E-8B49-EC2289BB81A9; **Taxon:** scientificName: Platyceruscaraboides (Linnaeus, 1758); order: Coleoptera; family: Lucanidae; **Location:** country: Italy; countryCode: IT; stateProvince: Vicenza; county: Valli del Pasubio; locality: Prealpi Venete, Monte Baffelan; verbatimElevation: 1700; **Event:** year: 1989; month: 6; day: 11; **Record Level:** collectionID: Coll. Università degli Studi di Padova
- **Type status:** Other material. **Occurrence:** recordedBy: leg. F. Giannone; individualCount: 1; sex: male; lifeStage: adult; occurrenceID: 4703E27A-4FCA-5C1E-893F-5B7B4F93E07F; **Taxon:** scientificName: Platyceruscaraboides (Linnaeus, 1758); order: Coleoptera; family: Lucanidae; **Location:** country: Italy; countryCode: IT; stateProvince: Vicenza; county: Zovencedo; locality: Zovencedo; **Event:** year: 1989; month: 9; day: 11; **Record Level:** collectionID: Coll. D. Scaccini
- **Type status:** Other material. **Occurrence:** individualCount: 1; lifeStage: adult; occurrenceID: 73DE09C3-1903-5C66-A57B-B46F5D1A9251; **Taxon:** scientificName: Platyceruscaraboides (Linnaeus, 1758); order: Coleoptera; family: Lucanidae; **Location:** country: Italy; countryCode: IT; stateProvince: Trento; municipality: Avio; locality: loc. Piazzo, M.te Baldo; decimalLatitude: 45.754839; decimalLongitude: 10.865619; geodeticDatum: WGS84; **Identification:** identifiedBy: det. P. Cornacchia; **Event:** year: 1938; month: 5; day: 15; **Record Level:** collectionID: Coll. Museo di Storia Naturale di Verona
- **Type status:** Other material. **Occurrence:** recordedBy: leg. Zanini; individualCount: 1; lifeStage: adult; occurrenceID: 4596E7E9-9A7C-5012-8B56-06FF924ABAB6; **Taxon:** scientificName: Platyceruscaraboides (Linnaeus, 1758); order: Coleoptera; family: Lucanidae; **Location:** country: Italy; countryCode: IT; stateProvince: Treviso; municipality: Vittorio Veneto; locality: (nearby, mountain); decimalLatitude: 45.985791; decimalLongitude: 12.288398; geodeticDatum: WGS84; **Identification:** identifiedBy: det. P. Cornacchia; **Event:** year: 1925; month: 4; **Record Level:** collectionID: Coll. Museo di Storia Naturale di Verona
- **Type status:** Other material. **Occurrence:** individualCount: 1; lifeStage: adult; occurrenceID: 3B683629-7EAF-5478-AE5E-2B6535DC78E7; **Taxon:** scientificName: Platyceruscaraboides (Linnaeus, 1758); order: Coleoptera; family: Lucanidae; **Location:** country: Italy; countryCode: IT; stateProvince: Verona; municipality: Grezzana; decimalLatitude: 45.517196; decimalLongitude: 11.006179; geodeticDatum: WGS84; **Identification:** identifiedBy: det. P. Cornacchia; **Event:** year: 1946; month: 4; day: 14; **Record Level:** collectionID: Coll. Museo di Storia Naturale di Verona
- **Type status:** Other material. **Occurrence:** recordedBy: leg. G. Sama; individualCount: 1; lifeStage: adult; occurrenceID: E826BB2C-2927-5F4E-BCFE-4497454C4950; **Taxon:** scientificName: Platyceruscaraboides (Linnaeus, 1758); order: Coleoptera; family: Lucanidae; **Location:** country: Italy; countryCode: IT; stateProvince: Forlì-Cesena; municipality: Sarsina; locality: Monteriolo; decimalLatitude: 43.874539; decimalLongitude: 12.122204; geodeticDatum: WGS84; **Identification:** identifiedBy: det. P. Cornacchia; **Event:** year: 1976; month: 4; **Record Level:** collectionID: Coll. G. Sama
- **Type status:** Other material. **Occurrence:** recordedBy: leg. P. Cornacchia & G. Scaglioni; individualCount: 1; lifeStage: adult; occurrenceID: E89D7813-8A9B-5E2C-8E01-C7EBC1E0399E; **Taxon:** scientificName: Platyceruscaraboides (Linnaeus, 1758); order: Coleoptera; family: Lucanidae; **Location:** country: Italy; countryCode: IT; stateProvince: Siena; municipality: Monticiano; locality: (nearby); decimalLatitude: 43.134354; decimalLongitude: 11.189692; geodeticDatum: WGS84; **Identification:** identifiedBy: det. P. Cornacchia; **Event:** year: 2002; month: 4; day: 27
- **Type status:** Other material. **Occurrence:** recordedBy: leg. P. Cornacchia & G. Scaglioni; individualCount: 2; lifeStage: adult; occurrenceID: 6FCAC2E4-EA64-52B9-AB4B-439BD507A315; **Taxon:** scientificName: Platyceruscaraboides (Linnaeus, 1758); order: Coleoptera; family: Lucanidae; **Location:** country: Italy; countryCode: IT; stateProvince: Teramo; municipality: Rocca Santa Maria; locality: Ceppo, Bosco Martese, M.ti della Laga; decimalLatitude: 42.669129; decimalLongitude: 13.444661; geodeticDatum: WGS84; **Event:** year: 2005; month: 5; day: 16
- **Type status:** Other material. **Occurrence:** recordedBy: leg. A.B. Biscaccianti, E. Castiglione, F. Manti; individualCount: 1; lifeStage: adult; occurrenceID: 875DAD20-6409-51D7-AF67-AA46BC5BF2C4; **Taxon:** scientificName: Platyceruscaraboides (Linnaeus, 1758); order: Coleoptera; family: Lucanidae; **Location:** country: Italy; countryCode: IT; stateProvince: Reggio Calabria; municipality: Samo; locality: torrente Apo Scipo, Vallone Fedavolito; decimalLatitude: 38.117667; decimalLongitude: 15.951639; geodeticDatum: WGS84; **Event:** year: 2015; month: 5
- **Type status:** Other material. **Occurrence:** recordedBy: leg. A.B. Biscaccianti, E. Castiglione, F. Manti; individualCount: 1; lifeStage: adult; occurrenceID: D36ECF2B-F162-5295-84AA-77FEFE19057D; **Taxon:** scientificName: Platyceruscaraboides (Linnaeus, 1758); order: Coleoptera; family: Lucanidae; **Location:** country: Italy; countryCode: IT; stateProvince: Reggio Calabria; municipality: Ciminà; locality: Piano Abbruschiato; decimalLatitude: 38.257778; decimalLongitude: 16.060389; geodeticDatum: WGS84; **Event:** year: 2016; month: 5
- **Type status:** Other material. **Occurrence:** recordedBy: leg. A.B. Biscaccianti; individualCount: 1; lifeStage: adult; occurrenceID: 246C5CF9-05A3-565B-94F7-347545214EC9; **Taxon:** scientificName: Platyceruscaraboides (Linnaeus, 1758); order: Coleoptera; family: Lucanidae; **Location:** country: Italy; countryCode: IT; stateProvince: Pescara; municipality: Sant'Eufemia a Maiella; locality: Roccacaramanico, M. Le Mucchia NE, Rava del Confine; decimalLatitude: 42.10958; decimalLongitude: 13.99201; geodeticDatum: WGS84; **Event:** year: 2020; month: 5; day: 14
- **Type status:** Other material. **Occurrence:** recordedBy: leg. A.B. Biscaccianti; individualCount: 1; lifeStage: adult; occurrenceID: 33CC7F20-450E-52ED-885B-C1BA9262A313; **Taxon:** scientificName: Platyceruscaraboides (Linnaeus, 1758); order: Coleoptera; family: Lucanidae; **Location:** country: Italy; countryCode: IT; stateProvince: L'Aquila; municipality: Pratola Peligna; locality: slavina in loc. L'Angotta; **Event:** year: 2006; month: 6; day: 9
- **Type status:** Other material. **Occurrence:** recordedBy: leg. A.B. Biscaccianti; individualCount: 1; lifeStage: adult; occurrenceID: 37CADCA2-8F9D-5525-A83F-FE9A3093005E; **Taxon:** scientificName: Platyceruscaraboides (Linnaeus, 1758); order: Coleoptera; family: Lucanidae; **Location:** country: Italy; countryCode: IT; stateProvince: L'Aquila; municipality: Pescocostanzo; locality: Bosco S. Antonio, La Difesa; **Event:** year: 2001; month: 5; day: 10-11
- **Type status:** Other material. **Occurrence:** recordedBy: leg. A.B. Biscaccianti, D. Di Santo; individualCount: 1; lifeStage: adult; occurrenceID: 37C02FEF-B614-5310-B67B-5FF5817B3F90; **Taxon:** scientificName: Platyceruscaraboides (Linnaeus, 1758); order: Coleoptera; family: Lucanidae; **Location:** country: Italy; countryCode: IT; stateProvince: Teramo; municipality: Pietracamela; locality: Corno Piccolo N, Bosco Aschiero; decimalLatitude: 42.500179; decimalLongitude: 13.576296; geodeticDatum: WGS84; **Event:** year: 2011; month: 6; day: 13
- **Type status:** Other material. **Occurrence:** recordedBy: leg. A.B. Biscaccianti, E. Lorenzetti; individualCount: 1; lifeStage: adult; occurrenceID: B04D8FAE-60D9-50A9-B3A0-F034210B0D59; **Taxon:** scientificName: Platyceruscaraboides (Linnaeus, 1758); order: Coleoptera; family: Lucanidae; **Location:** country: Italy; countryCode: IT; stateProvince: Rieti; municipality: Borbona; locality: Colle Pratoguerra N; decimalLatitude: 42.469695; decimalLongitude: 13.122413; geodeticDatum: WGS84; **Event:** year: 2007; month: 8; day: 1
- **Type status:** Other material. **Occurrence:** recordedBy: leg. A.B. Biscaccianti, E. Lorenzetti; individualCount: 1; lifeStage: adult; occurrenceID: 06CD644E-D7E7-5CDC-9C12-240D99D3D5B7; **Taxon:** scientificName: Platyceruscaraboides (Linnaeus, 1758); order: Coleoptera; family: Lucanidae; **Location:** country: Italy; countryCode: IT; stateProvince: Rieti; municipality: Borbona; locality: M. Cagno S; decimalLatitude: 42.468979; decimalLongitude: 13.112278; geodeticDatum: WGS84; **Event:** year: 2007; month: 6
- **Type status:** Other material. **Occurrence:** recordedBy: leg. Marsella & Rossi; individualCount: 3; lifeStage: adult; occurrenceID: EC8A4762-069F-5240-9936-7E5613BA43F6; **Taxon:** scientificName: Platyceruscaraboides (Linnaeus, 1758); order: Coleoptera; family: Lucanidae; **Location:** country: Italy; countryCode: IT; stateProvince: Arezzo; municipality: Poppi; locality: Fiume d'Isola; decimalLatitude: 43.805191; decimalLongitude: 11.868851; geodeticDatum: WGS84; **Event:** year: 2013; month: 4-5
- **Type status:** Other material. **Occurrence:** recordedBy: leg. D. Birtele, L. Zapponi, A. D'Amen; individualCount: 1; lifeStage: adult; occurrenceID: 366C8510-5521-55F9-81F1-1881637235CA; **Taxon:** scientificName: Platyceruscaraboides (Linnaeus, 1758); order: Coleoptera; family: Lucanidae; **Location:** country: Italy; countryCode: IT; stateProvince: Florence; municipality: Reggello; locality: Vallombrosa; decimalLatitude: 43.737951; decimalLongitude: 11.569052; geodeticDatum: WGS84; **Event:** year: 2013; month: 6; day: 6-24
- **Type status:** Other material. **Occurrence:** recordedBy: leg. F. Cussigh; individualCount: 1; sex: female; lifeStage: adult; occurrenceID: F9A7BB39-AEE5-523A-87CE-4D3DC2C11561; **Taxon:** scientificName: Platyceruscaraboides (Linnaeus, 1758); order: Coleoptera; family: Lucanidae; **Location:** country: Italy; countryCode: IT; stateProvince: Vicenza; municipality: Barbarano Mossano; locality: San Giovanni in Monte, Colli Berici; **Identification:** identifiedBy: det. F. Cussigh; **Event:** year: 2001; month: 5; day: 4; **Record Level:** collectionID: Coll. Museo Naturalistico Archeologico, Vicenza (ex. Coll. F. Cussigh); collectionCode: Collection code: mnav-ent.fc-04516
- **Type status:** Other material. **Occurrence:** recordedBy: leg. F. Cussigh; individualCount: 2; sex: 1 male, 1 female; lifeStage: adult; occurrenceID: E797FB3C-DAB1-5C9D-958B-F6506CA34274; **Taxon:** scientificName: Platyceruscaraboides (Linnaeus, 1758); order: Coleoptera; family: Lucanidae; **Location:** country: Italy; countryCode: IT; stateProvince: Vicenza; municipality: Caltrano; locality: Costo (Asiago); **Identification:** identifiedBy: det. F. Cussigh; **Event:** year: 1996; month: 5; day: 5; **Record Level:** collectionID: Coll. Museo Naturalistico Archeologico, Vicenza (ex. Coll. F. Cussigh); collectionCode: Collection codes: mnav-ent.fc-09699, mnav-ent.fc-09705
- **Type status:** Other material. **Occurrence:** recordedBy: leg. F. Cussigh; individualCount: 1; sex: male; lifeStage: adult; occurrenceID: 00C7FAA2-F9DB-5E63-A530-65CA9987FED6; **Taxon:** scientificName: Platyceruscaraboides (Linnaeus, 1758); order: Coleoptera; family: Lucanidae; **Location:** country: Italy; countryCode: IT; stateProvince: Vicenza; municipality: Isola Vicentina; locality: Torreselle; **Identification:** identifiedBy: det. F. Cussigh; **Event:** year: 1989; month: 4; day: 30; **Record Level:** collectionID: Coll. Museo Naturalistico Archeologico, Vicenza (ex. Coll. F. Cussigh); collectionCode: Collection code: mnav-ent.fc-09700
- **Type status:** Other material. **Occurrence:** recordedBy: leg. F. Cussigh; individualCount: 1; sex: male; lifeStage: adult; occurrenceID: 9AED86CE-8319-5A2C-8331-76B9E21EBA75; **Taxon:** scientificName: Platyceruscaraboides (Linnaeus, 1758); order: Coleoptera; family: Lucanidae; **Location:** country: Italy; countryCode: IT; stateProvince: Vicenza; municipality: Roana; locality: Camporovere (Asiago); **Identification:** identifiedBy: det. F. Cussigh; **Event:** year: 1987; month: 5; day: 30; **Record Level:** collectionID: Coll. Museo Naturalistico Archeologico, Vicenza (ex. Coll. F. Cussigh); collectionCode: Collection code: mnav-ent.fc-09703
- **Type status:** Other material. **Occurrence:** recordedBy: leg. F. Cussigh; individualCount: 1; sex: male; lifeStage: adult; occurrenceID: B828D7A8-8EA2-5187-B32F-7CA130B8C370; **Taxon:** scientificName: Platyceruscaraboides (Linnaeus, 1758); order: Coleoptera; family: Lucanidae; **Location:** country: Italy; countryCode: IT; stateProvince: Vicenza; municipality: Valdastico; locality: Montepiano; **Identification:** identifiedBy: det. F. Cussigh; **Event:** year: 1993; month: 5; day: 2; **Record Level:** collectionID: Coll. Museo Naturalistico Archeologico, Vicenza (ex. Coll. F. Cussigh); collectionCode: Collection code: mnav-ent.fc-09698
- **Type status:** Other material. **Occurrence:** recordedBy: leg. F. Cussigh; individualCount: 2; sex: 1 male, 1 female; lifeStage: adult; occurrenceID: 9983C6DE-4FC9-5B8C-BE8B-82DB5FFA3742; **Taxon:** scientificName: Platyceruscaraboides (Linnaeus, 1758); order: Coleoptera; family: Lucanidae; **Location:** country: Italy; countryCode: IT; stateProvince: Vicenza; municipality: Valdastico; locality: Montepiano; **Identification:** identifiedBy: det. F. Cussigh; **Event:** year: 1991; month: 5; day: 21; **Record Level:** collectionID: Coll. Museo Naturalistico Archeologico, Vicenza (ex. Coll. F. Cussigh); collectionCode: Collection codes: mnav-ent.fc-09701, mnav-ent.fc-09704
- **Type status:** Other material. **Occurrence:** recordedBy: leg. F. Cussigh; individualCount: 1; sex: male; lifeStage: adult; occurrenceID: 7E002ADA-D76D-5DBF-91DB-B041592A66B9; **Taxon:** scientificName: Platyceruscaraboides (Linnaeus, 1758); order: Coleoptera; family: Lucanidae; **Location:** country: Italy; countryCode: IT; stateProvince: Vicenza; locality: Pian delle Fugazze (Valli del Pasubio); **Identification:** identifiedBy: det. F. Cussigh; **Event:** year: 1985; month: 5; day: 25; **Record Level:** collectionID: Coll. Museo Naturalistico Archeologico, Vicenza (ex. Coll. F. Cussigh); collectionCode: Collection code: mnav-ent.fc-09702
- **Type status:** Other material. **Occurrence:** recordedBy: leg. S. Biondi; individualCount: 2; sex: male; lifeStage: adult; occurrenceID: 983C6758-72D5-53CA-8EAD-2E38876CB771; **Taxon:** scientificName: Platyceruscaraboides (Linnaeus, 1758); order: Coleoptera; family: Lucanidae; **Location:** country: Italy; countryCode: IT; stateProvince: Vicenza; municipality: Arcugnano; locality: Lapio, surroundings (Colli Berici); **Identification:** identifiedBy: det. D. Scaccini; **Event:** year: 1985; month: 5; day: 5; **Record Level:** collectionID: Coll. S. Biondi
- **Type status:** Other material. **Occurrence:** recordedBy: leg. S. Biondi; individualCount: 2; sex: male; lifeStage: adult; occurrenceID: 8849BB80-64D0-57AE-89B3-9A7568EF9796; **Taxon:** scientificName: Platyceruscaraboides (Linnaeus, 1758); order: Coleoptera; family: Lucanidae; **Location:** country: Italy; countryCode: IT; stateProvince: Vicenza; locality: Monte Summano; **Identification:** identifiedBy: det. D. Scaccini; **Event:** year: 1979; month: 5; day: 19; **Record Level:** collectionID: Coll. S. Biondi
- **Type status:** Other material. **Occurrence:** recordedBy: leg. S. Biondi; individualCount: 1; sex: female; lifeStage: adult; occurrenceID: 038EA0A3-FD70-5C9B-9191-22821F8945C2; **Taxon:** scientificName: Platyceruscaraboides (Linnaeus, 1758); order: Coleoptera; family: Lucanidae; **Location:** country: Italy; countryCode: IT; stateProvince: Vicenza; municipality: Lastebasse; locality: Montepiano, surroundings; **Identification:** identifiedBy: det. D. Scaccini; **Event:** year: 1984; month: 6; day: 18; **Record Level:** collectionID: Coll. S. Biondi
- **Type status:** Other material. **Occurrence:** recordedBy: leg. S. Biondi; individualCount: 1; sex: male; lifeStage: adult; occurrenceID: 320C3670-D700-533B-B93D-E8CF3F2E0BDE; **Taxon:** scientificName: Platyceruscaraboides (Linnaeus, 1758); order: Coleoptera; family: Lucanidae; **Location:** country: Italy; countryCode: IT; stateProvince: Vicenza; municipality: Laghi; **Identification:** identifiedBy: det. D. Scaccini; **Event:** year: 1987; month: 5; day: 10; **Record Level:** collectionID: Coll. S. Biondi
- **Type status:** Other material. **Occurrence:** recordedBy: leg. S. Biondi; individualCount: 2; sex: 1 male, 1 female; lifeStage: adult; occurrenceID: 830874A0-B138-5C2F-9635-3CDC2DB852AC; **Taxon:** scientificName: Platyceruscaraboides (Linnaeus, 1758); order: Coleoptera; family: Lucanidae; **Location:** country: Italy; countryCode: IT; stateProvince: Vicenza; municipality: Cogollo del Cengio; **Identification:** identifiedBy: det. D. Scaccini; **Event:** year: 1989; month: 5; day: 6; **Record Level:** collectionID: Coll. S. Biondi
- **Type status:** Other material. **Occurrence:** recordedBy: leg. S. Biondi; individualCount: 1; sex: male; lifeStage: adult; occurrenceID: 93A22730-49B2-572E-BCD8-ECBA20FA9E0D; **Taxon:** scientificName: Platyceruscaraboides (Linnaeus, 1758); order: Coleoptera; family: Lucanidae; **Location:** country: Italy; countryCode: IT; stateProvince: Perugia; municipality: Gubbio; locality: surroundings; **Identification:** identifiedBy: det. D. Scaccini; **Event:** year: 2001; month: 4; day: 30; **Record Level:** collectionID: Coll. S. Biondi
- **Type status:** Other material. **Occurrence:** recordedBy: leg. S. Biondi; individualCount: 1; sex: male; lifeStage: adult; occurrenceID: B4441619-E67E-547F-990D-738105103315; **Taxon:** scientificName: Platyceruscaraboides (Linnaeus, 1758); order: Coleoptera; family: Lucanidae; **Location:** country: Italy; countryCode: IT; stateProvince: Trento; municipality: Primiero San Martino Castrozza; locality: Transacqua; **Identification:** identifiedBy: det. D. Scaccini; **Event:** year: 2002; month: 4; day: 1; **Record Level:** collectionID: Coll. S. Biondi
- **Type status:** Other material. **Occurrence:** recordedBy: leg. S. Biondi; individualCount: 1; sex: male; lifeStage: adult; occurrenceID: 0248EBB4-099D-5873-9446-1DC43EA08D27; **Taxon:** scientificName: Platyceruscaraboides (Linnaeus, 1758); order: Coleoptera; family: Lucanidae; **Location:** country: Italy; countryCode: IT; stateProvince: Vicenza; municipality: Arcugnano; locality: Lago di Fimon, Colli Berici; **Identification:** identifiedBy: det. D. Scaccini; **Event:** year: 2012; month: 4; day: 27; **Record Level:** collectionID: Coll. S. Biondi

#### Distribution

**Figure 6.**
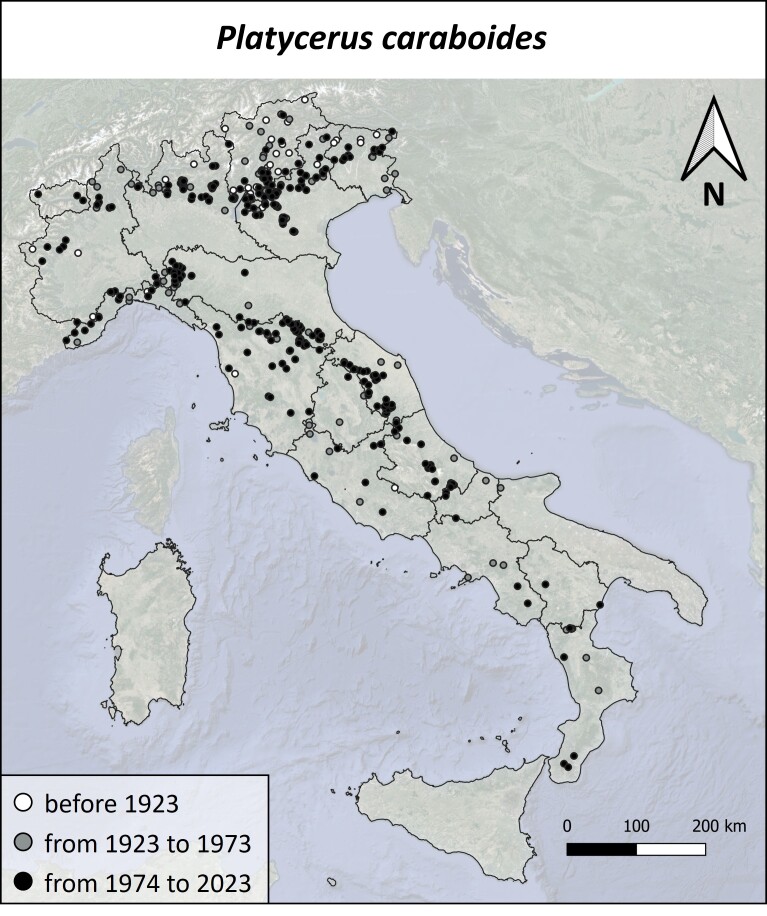
Distribution map of *Platyceruscaraboides* in Italy, featuring findings from different collection periods. Updated to 31 December 2023.

The study of the available material resulted in 187 new distributional records, an increase of more than 70% over what is known from literature. *Platyceruscaraboides* is, thus, known for 453 Italian localities, but it is absent from Sicily and Sardinia (Fig. 6). The species has been observed most frequently in the Alps and the central Apennines, while records for southern Italy are rather sporadic. Occurrences for this species are largely localised in hilly and mountainous areas, although there are a few records at lower altitudes. The temporal distribution of *P.caraboides* occurrences is as follows: 43 observations (9.5%) were collected prior to 1923, 87 (19.2%) were collected between 1923 and 1973 and 323 (71.3%) from 1973 to present (Suppl. material 1).

#### Ecology

**Figure 7.**
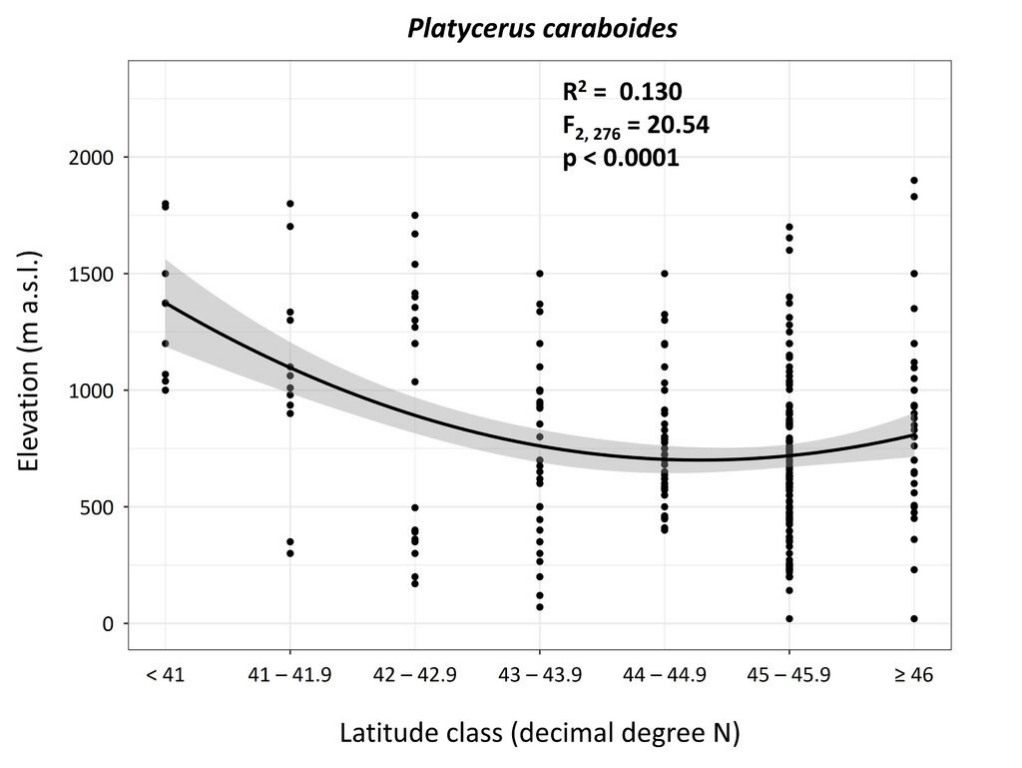
Quadratic regression on *Platyceruscaraboides* finding locations for the relationship between latitude and elevation in Italy. F statistic, R^2^ and p values are reported in the graph.

For the available sites (n = 279), the elevation was 782.65 ± 385.69 m a.s.l. (mean ± st. dev.), ranging from 20 to 1900 m a.s.l. The analysis on latitude-elevation revealed a significant relationship for both linear (F_1, 277_ = 20.01, p < 0.001) and quadratic regressions (Fig. 7). AIC scores (AIC_linear_: 4101.22; AIC_quadratic_: 4083.96) and the ANOVA test (p < 0.001) confirmed that the quadratic model best fits the data. Findings indicated a decrease in elevation with an increase in latitude, particularly at lower latitudes (Fig. 7). Similarly to what was observed for *P.caprea*, the activity of *P.caraboides* adults peaked in April–June.

## Discussion

The present research substantially increased the current knowledge of the occurrence of *P.caprea* and *P.caraboides* in Italy, providing more than 700 new locality records combined. Except for Sardinia and Sicily, *P.caprea* and *P.caraboides* are recorded in all Italian regions, despite some parts of the Italian Peninsula still being under investigation. The Apulia Region is the only one where the species were recorded in historical times and never to be found again. Despite records suggesting that the species might be present along the most important hill and mountain ranges of Italy, both taxa indicate a general preference for those parts of the Alps and the Apennines characterised by cool and sub-continental climates (Fratianni and Acquaotta 2017). The analysis revealed a significant inverse relationship between latitude and elevation concerning the occurrence of *P.caraboides*; a similar trend was noted for *P.caprea*, although it was not statistically significant, likely due to the limited available data. It should be noted that, unlike what can be observed for other European stag beetles (e.g. Romiti et al. 2017, Thomaes et al. 2021, Scaccini et al. 2023), latitude-related features are still not known for these species, emphasising the need for further studies on *Platycerus*.

*Platyceruscaraboides* appeared to be more commonly found than *P.caprea* and this could be related to their habitat requirements, such as elevation, microclimatic conditions and deadwood type and amount in the forest (e.g. Franciscolo 1997, Bartolozzi and Maggini 2007, Klausnitzer and Sprecher-Uebersax 2008, Lachat et al. 2012, Scaccini 2022). Given the fact that *Platycerus* adults are elusive and have a short flight period, the identification of oviposition scars on deadwood has proven to be a useful method for locating sites where these two species are present, as suggested in Scaccini (2016) and Scaccini (2022).

Although this contribution has attempted to update and summarise as much knowledge as possible on the distribution of these two stag beetle species, there are still important missing data locally in certain areas of the Italian Peninsula. Furthermore, in light of the new observations reported, it would be important to reconsider their conservation status in Italy; the two *Platycerus* are currently considered “Least Concern”, but this categorisation may be more the result of a lack of information at the time of the compilation of the Red List of saproxylic beetles than representing the species true conservation status. Indeed, although they are not considered amongst the most threatened species, given their bioecological characteristics and the peculiar distribution pattern on the Italian territory, a targetted assessment of the susceptibility of these two taxa to range contraction or extinction of local populations associated with habitat reduction and fragmentation and climate change becomes essential.

## Supplementary Material

XML Treatment for Platycerus caprea

XML Treatment for Platycerus caraboides caraboides

7778C3C5-5A6A-50E0-8222-9BD50AF8FA93 10.3897/BDJ.12.e127088.suppl1 Supplementary material 1 Occurrences of *Platyceruscaprea* and *Platyceruscaraboides* in Italy, based on the published material. Data type Occurrences Brief description Table reporting information on the occurrences of *Platyceruscaprea* and *Platyceruscaraboides* in Italy, based on the published material. File: oo_1030525.xlsx https://binary.pensoft.net/file/1030525 Scaccini D, Bartolozzi L, Zilioni M, Di Giulio A, Ruzzier E

## Figures and Tables

**Figure 2.**
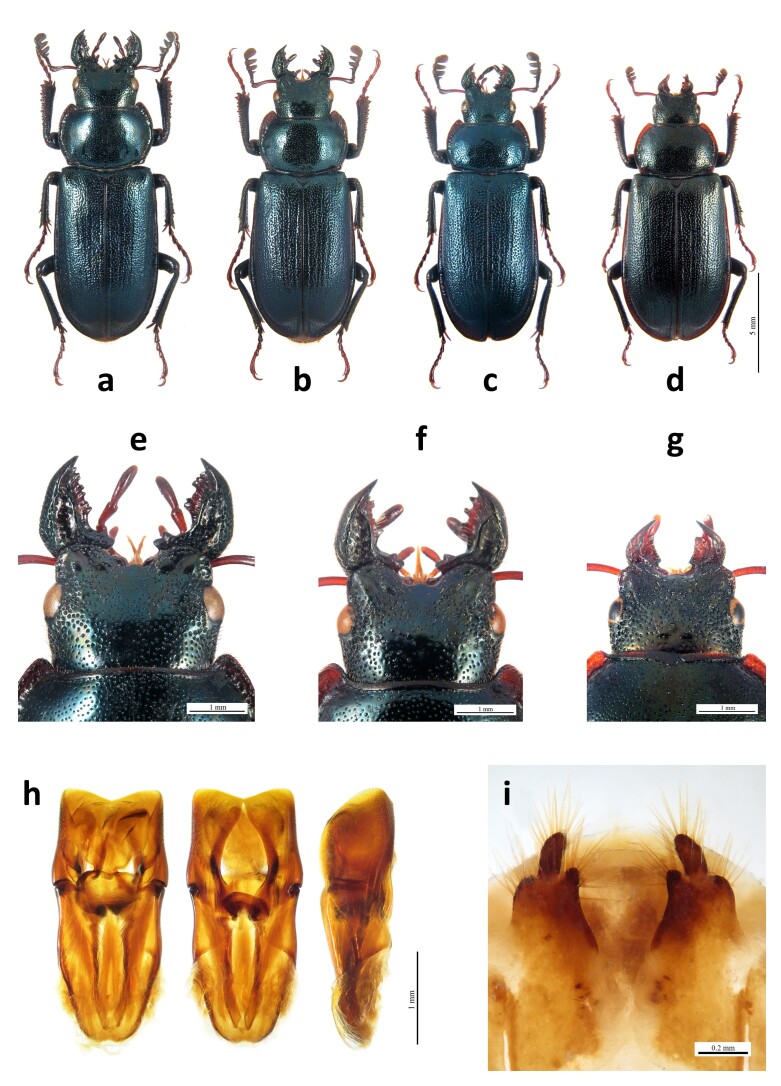
Habitus of *Platyceruscaprea* adults collected in Italy. **a** Male, Vercelli Province, Alagna Valsesia, 16.VI.2012; **b** Male, Bergamo Province, Zambla, 19.X.1969; **c** Male, Bergamo Province, Zambla, 19.X.1969; **d** Female, Bergamo Province, Zambla, 19.X.1969; **e** Magnification of the head, specimen in (a); **f** Magnification of the head, specimen in (b); **g** Magnification of the head, specimen in (d); **h** Male genitalia, specimen in (b); **i** Female styli, specimen in (d). Photo credit: M. Zilioli.

**Figure 3.**
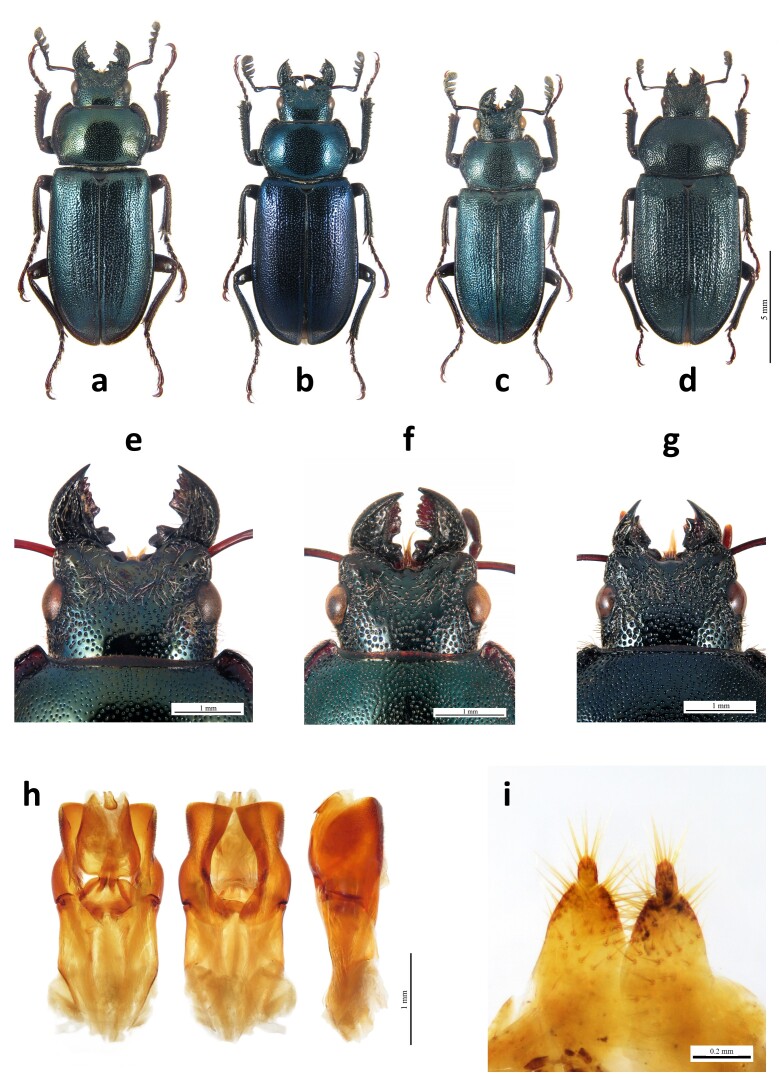
Habitus of *Platyceruscaraboides* adults collected in Italy. **a** Male, Alpi Cozie, Val di Susa (collection date is not available); **b** Male, Bolzano Province, Tires, 11.VI.2010; **c** Male, Piacenza Province, Ferriere (surroundings), 8.V.1969; **d** Female, Bergamo Province, Piazzolo, 14.VIII.2013; **e** Magnification of the head, specimen in (a); **f** Magnification of the head, specimen in (c), re-pinned; **g** Magnification of the head, specimen in (d); **h** Male genitalia, specimen in (a); **i** Female styli, specimen in (d). Photo credit: M. Zilioli.
